# Azobenzene Reduction and Derivatization and Al–H Bond Insertion with β‑Diketiminate Gallium(I) Complexes

**DOI:** 10.1021/acs.organomet.6c00019

**Published:** 2026-02-25

**Authors:** Huanhuan Dong, Connor Bourne, Aidan P. McKay, Alexandra M. Z. Slawin, David B. Cordes, Andreas Stasch

**Affiliations:** EaStCHEM School of Chemistry, 7486 University of St Andrews, North Haugh, St Andrews KY16 9ST, United Kingdom

## Abstract

Using backbone modification of popular β-diketiminate ligands, ^RDip^nacnac = HC­(RCNDip)_2_, with R = Et, *i*Pr (Dip = 2,6-*i*Pr_2_C_6_H_3_), we have prepared the new β-diketiminate gallium­(I) complexes [(^EtDip^nacnac)­Ga] **2a** and [(^*i*PrDip^nacnac)­Ga] **2b** by salt metathesis/reduction using “GaI” in moderate to good (68%, **2a**) and poor (10%, **2b**) isolated yields, respectively, highlighting the influence of the ligand backbone substitution on the reaction success. The gallium­(I) complexes were converted with azobenzene to the gallium­(III) complexes [(^RDip^nacnac)­Ga­{(C_6_H_5_)­NNPh}] **4a** (R = Et) and **4b** (R = *i*Pr) with reduced former azobenzene fragments showing an *N*,*ortho*-C­(H)-chelating coordination to the Ga centers. Complex **4a** was further converted to its C–H-activated tautomer [(^EtDip^nacnac)­Ga­{(C_6_H_4_)­N­(H)­NPh}] **5**, and reaction with DMSO and benzaldehyde afforded [(^EtDip^nacnac)­Ga­(PhNNHPh)­(CH_2_S­(O)­Me)] **6** after DMSO deprotonation and [(^EtDip^nacnac)­Ga­(PhNN­(Ph)­CH­(Ph)­O)] **7** from C–N coupling, respectively. Compound **2a** also reacted with the aluminum­(III) hydride complexes (NHC)­AlH_3_ (NHC = {MeCN­(*i*Pr)}_2_C) and (Me_3_N)­AlH_3_ to the Ga–Al-bonded complex [(^EtDip^nacnac)­Ga­(H)–Al­(H_2_)­(NHC)] **8** and [(^EtDip^nacnac)­GaH_2_] **9**, respectively. Gallium­(I) complex **2a** is a good alternative to commonly used [(^MeDip^nacnac)­Ga] (R = Me) for the study and application of low-oxidation-state gallium complexes.

## Graphical abstract

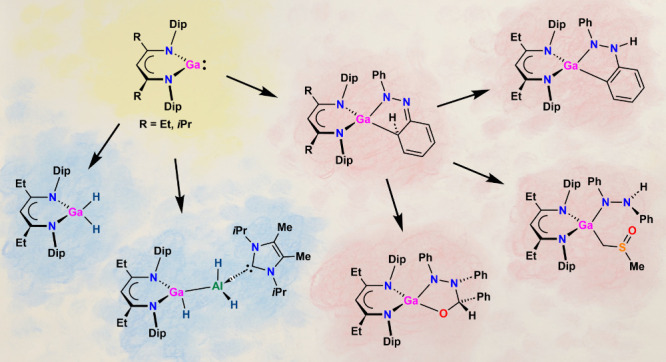

## Introduction

The reactivity of heavier group 13 and 14 element carbene analogues with unsaturated small molecules has been extensively explored over the last two decades,^1–5^ driven by the unusual and diverse reaction pathways they display, including some transition metal-like behavior.^6–8^ In group 13, the generally higher stability of low-oxidation-state gallium compounds compared to their aluminum congeners has allowed the chemistry of a wide range of readily prepared gallium species to be explored.^1–3^ Significant impetus came from a family of monomeric gallium­(I) complexes with N,N-chelating ligands and two-coordinate Ga centers based on the discoveries of diazabutadienediide (**I**)^9,10^ and β-diketiminate (**II**)^11–16^ complexes, see Figure 1, reported a quarter of a century ago. This was followed by related examples with four-,^17–19^ five-,^20,21^ and six-membered^22–25^ chelates and larger ring systems^26^ based on organic and iminophosphorane N-ligand systems.

**Figure 1:**
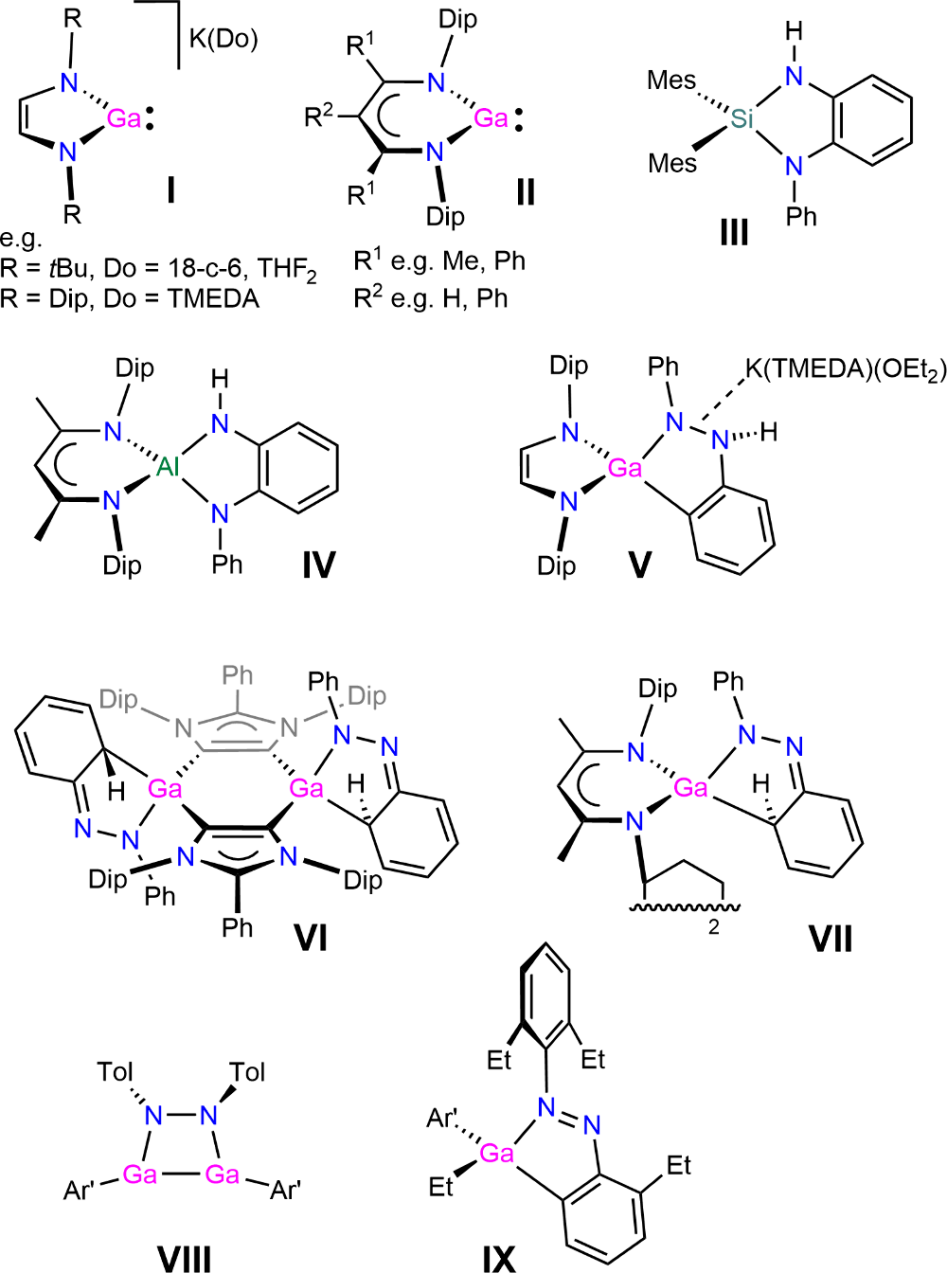
Selected N,N-chelated gallium­(I) complexes (**I** and **II**) and reaction products of selected p-block compounds with diazenes (**III–** **IX**). Note that formal charges in zwitterionic **V** and **VI** are not shown. 18-c-6 = 18-crown-6, TMEDA = *N*,*N*,*N*′,*N*′-tetramethylethane-1,2-diamine, Dip = 2,6-*i*Pr_2_C_6_H_3_, Mes = 2,4,6-Me_3_C_6_H_2_, Tol = 4-MeC_6_H_4_, and Ar′ = 2,6-Dip_2_C_6_H_3_.

Reactions of diazenes, RN=NR,^27–29^ such as azobenzene, with low-oxidation state *p*-block compounds, have been studied largely in parallel with the availability of new low-oxidation-state *p*-block compounds and have often provided unexpected products that involve N–N bond cleavage, ortho-C–H-activation, and other unusual processes. In early work, the in situ generated silylene Mes_2_Si reacted with azobenzene to the rearranged product **III**;^30^ the same class of reduction product (**IV**) that was isolated when using Roesky’s β-diketiminate aluminum­(I) complex as the main group reductant (Figure 1).^31^ Reactions of alkali metal diazabutadienediide gallium­(I) species (e.g., **I**) afforded C–H-activated products (e.g., **V**)^32,33^ with azobenzenes that likely proceed via dearomatized intermediates similar to those characterized only very recently, **VI** ^34^ and **VII**; the latter could also be converted to a rearomatized C–H-activated example related to **V**.^35^ Other product outcomes starting from terphenyl-stabilized gallium­(I) complexes are the N=N-inserted complex **VIII** and the C–C-activated product **IX** that highlight the diverse reactivity of azobenzene with low-oxidation-state gallium complexes.^36^

## Results and Discussion

We have recently introduced the backbone-modified β-diketiminate ligands ^RDip^nacnac = HC­(RCNDip)_2_, R = Et, *i*Pr,^37,38^ to low-oxidation-state and low-coordination-mode chemistry with the aim of improving the chemical robustness of this popular spectator ligand class and have also found that the steric modifications can have very significant effects on its metal complex chemistry.^39,40^ These ligand modifications have an impact on the stability and “innocence” of various parts of the β-diketiminate ligand scaffold including the multiple activation modes that have been observed involving the backbone unit.^41^ To investigate the chemistry of these ligands with low-oxidation-state gallium centers and in analogy to the synthesis of other β-diketiminate gallium­(I) complexes (**II**),^11–16^ we treated the lithium complexes of the above ligands, [(^RDip^nacnac)­Li] **1a** (R = Et) and **1b** (R = *i*Pr),^38^ with a freshly prepared suspension of “GaI” ^42,43^ in toluene at 0 °C, followed by slowly warming to room temperature and stirring overnight. Workup afforded the yellow, crystalline gallium­(I) complexes [(^EtDip^nacnac)­Ga] **2a** and [(^*i*PrDip^nacnac)­Ga] **2b**, see Scheme 1. The synthesis of **2a** provided the gallium­(III) iodide complex [(^EtDip^nacnac)­GaI_2_] **3a** as the main byproduct. Accordingly, the synthesis was repeated with an additional reduction step using substoichiometric potassium graphite addition to afford compound **2a** in 68% isolated yield. This compares favorably to the reported yield for [(^MeDip^nacnac)­Ga] (39%)^11^ using a similar route via “GaI”, although a yield of 66% can be obtained from reacting [(^MeDip^nacnac)­Na] and Cp*Ga over 4 days. This does, however, require preparation of the organometallic gallium­(I) precursor.^44^ The synthesis of [(^*i*PrDip^nacnac)­Ga] **2b**, with or without the KC_8_ reduction step, always provided significant byproduct formation of the proligand ^*i*PrDip^nacnacH alongside **2b**, and thus, **2b** was only isolated in ca. 10% yield by fractional crystallization from *n*-hexane. Using [(^*i*PrDip^nacnac)­K][Bibr ref38] as the β-diketiminate source instead of **1b** led to a product mixture and did not improve the yield of **2b**. We ascribe the low yielding formation of **2b** to the higher steric demand and increased basicity (electronic effect) of the β-diketiminate anion, which likely leads to hydrogen abstraction reactions in the reaction mixture for the ^*i*PrDip^nacnac anion. The lower solubility of some complexes and intermediates of this ligand, **b** compounds, likely also plays a role in this, for example, by slowing down desired salt metathesis reactivity. We have furthermore prepared the gallium­(III) iodide complexes [(^EtDip^nacnac)­GaI_2_] **3a** and [(^*i*PrDip^nacnac)­GaI_2_] **3b** by a related reaction of **1a** and **1b** with GaI_3_, respectively, in good yields (72% and 65%). Complex **3a** could alternatively also be reduced to gallium­(I) complex **2a** using KC_8_ in ca. 71% yield, but this approach was again not practical for the synthesis of **2b** and typically provided product mixtures containing **3b** and ^*i*PrDip^nacnacH (Scheme 1).

**Scheme 1:**
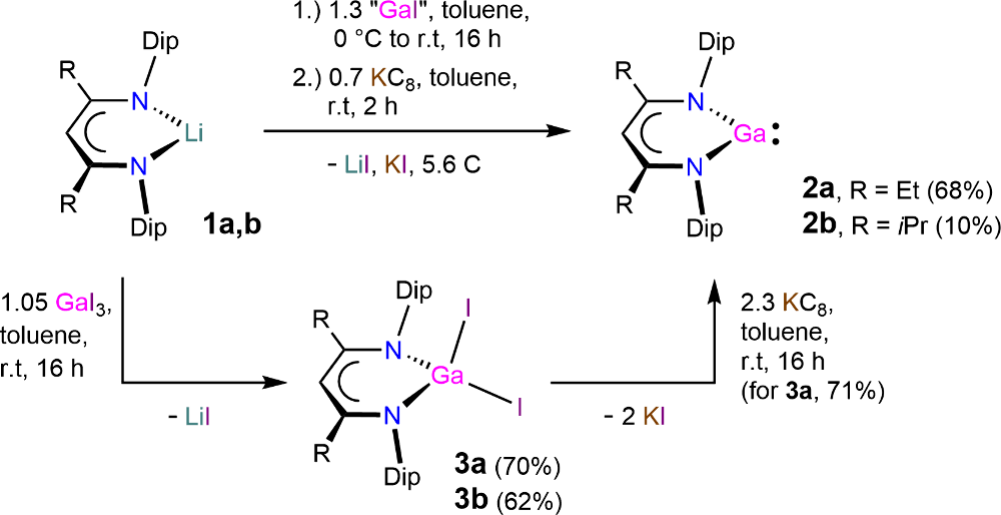
Synthesis of Compounds **2a**, **2b**, **3a**, and **3b**

The complexes [(^EtDip^nacnac)­Ga] **2a** (Figure 2), [(^*i*PrDip^nacnac)­Ga] **2b** (Figure 3), and [(^*i*PrDip^nacnac)­GaI_2_] **3b** (Figure 4) were structurally characterized. Complexes **2a** and **2b** show the expected structures with two-coordinate Ga centers that lie in the β-diketiminate plane, as in previously characterized examples.^11–16^ The molecular structure of the gallium­(III) iodide complex **3b** shows the Ga center to be located out of the ^*i*PrDip^nacnac plane by ca. 0.67 Å in a similar manner to those of related precursor complexes. The Ga center is in a distorted tetrahedral coordination environment, and the Ga1–I1 bond, which is 0.05 Å longer than the Ga1–I2 bond, is essentially orthogonal to the ^*i*PrDip^nacnac plane, whereas the I2 atom lies approximately in the ^*i*PrDip^nacnac plane.

**Figure 2:**
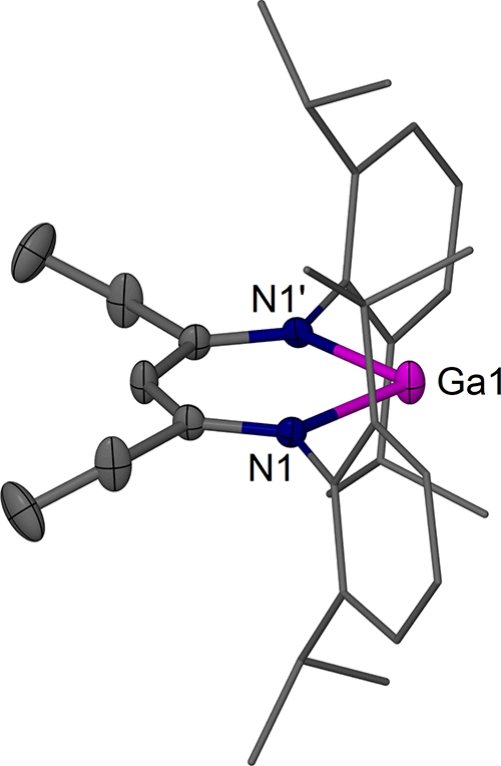
Molecular structure (30% thermal ellipsoids) of [(^EtDip^nacnac)­Ga] **2a**. Hydrogen atoms and minor components of disorder have been omitted for the sake of clarity. Dip groups are shown as wireframe. Selected bond lengths (Å) and angles (°): Ga(1)–N(1) 2.052(2), Ga(1)–N(1)′ 2.052(2); N(1)–Ga(1)–N(1)′ 87.97(13), Ga(1)–N(1)–C(4) 110.05(18).

**Figure 3:**
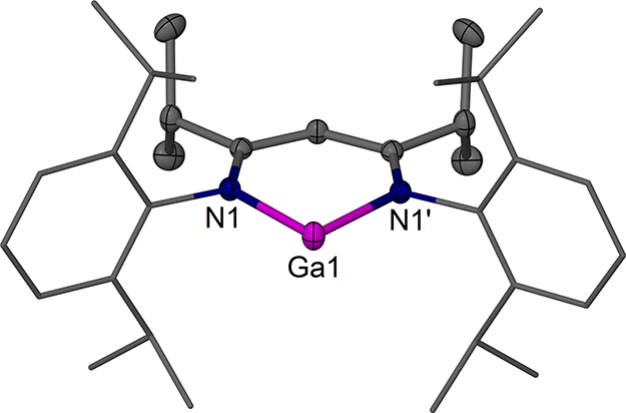
Molecular structure (30% thermal ellipsoids) of [(^*i*PrDip^nacnac)­Ga]·3 C_6_H_6_, **2b**·3 C_6_H_6_. Hydrogen atoms and solvent molecules have been omitted for clarity. Dip groups are shown as wireframe. Selected bond lengths (Å) and angles (°): Ga(1)–N(1) 2.054(2), Ga(1)–N(1)′ 2.054(2); N(1)–Ga(1)–N(1)′ 87.84(13), Ga(1)–N(1)–C(4) 110.18(16).

**Figure 4:**
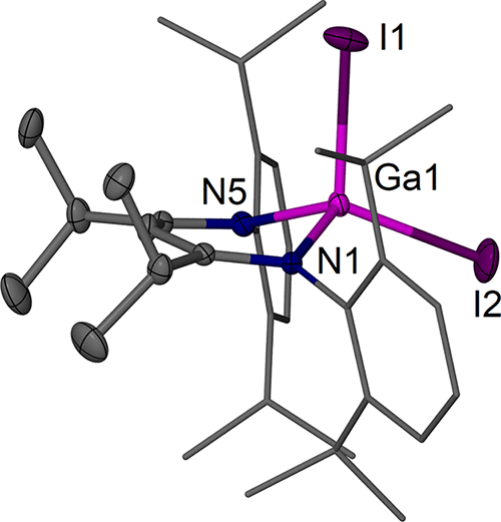
Molecular structure (30% thermal ellipsoids) of [(^*i*PrDip^nacnac)­GaI_2_] **3b**. Hydrogen atoms are omitted for clarity, and Dip groups are shown as wireframe. Selected bond lengths (Å) and angles (°): I1–Ga1 2.5418(3), I2–Ga1 2.4901(3), Ga(1)–N(1) 1.9283(18), Ga(1)–N(5) 1.9368(19); I2–Ga1–I1 111.167(13), N1–Ga1–N5 100.00(8).

The ^1^H NMR spectra of complexes **2a**, **2b**, **3a**, and **3b** show the expected predominantly sharp resonances expected for complexes with ^RDip^nacnac ligands in a symmetric environment, i.e., with one septet and two doublets for the hydrogen atoms of the Dip-*i*Pr substituents.

With [(^RDip^nacnac)­Ga] **2a** and **2b** in hand, we investigated their reaction toward azobenzene. Compound **2a** reacted with 1 equiv of azobenzene in deuterated benzene cleanly and rapidly to form a dark orange solution of a product with one β-diketiminate CH backbone singlet and further resonances that suggest the inequivalence of all Dip isopropyl hydrogen environments and upfield-shifted resonances of former aromatic groups that suggest a dearomatization reaction. The new complex [(^EtDip^nacnac)­Ga­{(C_6_H_5_)­NNPh}] **4a** was formed essentially quantitatively in deuterated benzene or could be isolated as orange block-like crystals in ca. 80% yield from a concentrated *n*-hexane solution. [(^*i*PrDip^nacnac)­Ga] **2b** was similarly converted with azobenzene to a related compound, [(^*i*PrDip^nacnac)­Ga­{(C_6_H_5_)­NNPh}] **4b**, within 1 h in ca. 90% in situ yield, see Scheme 2.

**Scheme 2:**
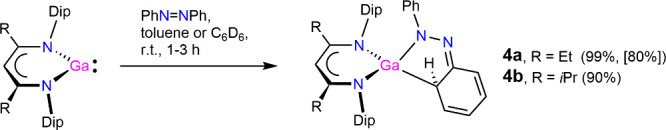
Synthesis of Compounds **4a** and **4b**

The new complexes **4a** (Figure 5) and **4b** (Figure 6) were structurally characterized and show the formation of an identical former azobenzene fragment with one dearomatized former phenyl group coordinated to a ^RDip^nacnac-coordinated gallium­(III) center resulting from an oxidative addition reaction similar to that reported for complexes **VI** and **VII**.^34,35^ Key bond lengths are given in Figure 7. Complexes **4a** and **4b** show an N,C-chelating reduced azobenzene ligand forming a five-membered ring system with the Ga center. The main plane of the new ligand lies approximately orthogonal to the β-diketiminate plane. The Ga–C coordination leads to dearomatization of one phenyl group of the former azobenzene moiety, resulting in pyramidalization of the Ga-bound ortho-CH position and deformation of the ring such that this CH is not in-plane anymore with the other five ring carbons. This position is affected by disorder, but the main positions refine well, show reliable C–C bond lengths of ca. 1.50 Å around the Ga–CH position (Figure 7), and thus suggest single bond characteristics. The rest of the bond lengths in the reduced former phenyl ring as well as the bonding to nitrogen show alternating short and long bond distances that support dearomatization and significant localization of the bonds in the unsaturated coplanar system. The N–N bond is elongated compared to an N=N double bond, and the N=C bond length at the former phenyl group shows imine character. The overall trends in bond length are similar to those in **VI** and **VII**.^34,35^ One ortho (C−)H center of the remaining phenyl group shows a short contact (2.67 Å mean for the molecular structures of **4a** and **4b**) to the electron-rich central β-diketiminate backbone carbon atom.

**Figure 5:**
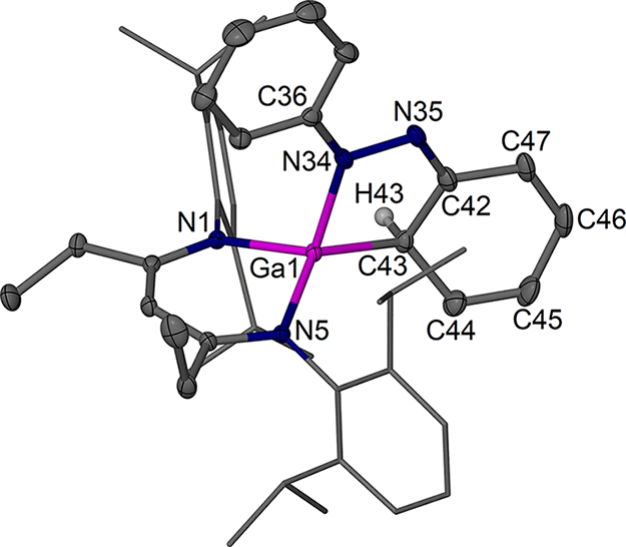
Molecular structure (30% thermal ellipsoids) of [(^EtDip^nacnac)­Ga­{(C_6_H_5_)­NNPh}] **4a**. Hydrogen atoms, except for H43, and minor components of disorder have been omitted for clarity. Dip groups are shown as wireframe. Selected bond lengths (Å) and angles (°): Ga1–N1 1.9441(12), Ga1–N5 1.9255(12), Ga1–N34 1.9214(13), Ga1–C43 1.977(2); N5–Ga1–N1 96.51(5), N1–Ga1–C43 114.69(12), N5–Ga1–C43 131.29(11), N34–Ga1–N1 110.06(5), N34–Ga1–N5 117.28(5), N34–Ga1–C43 87.10(7), C36–N34–N35 115.18(12), C42–N35–N34 113.97(13), N35–C42–C43 122.67(15). Further distances are collected in Figure 7.

**Figure 6:**
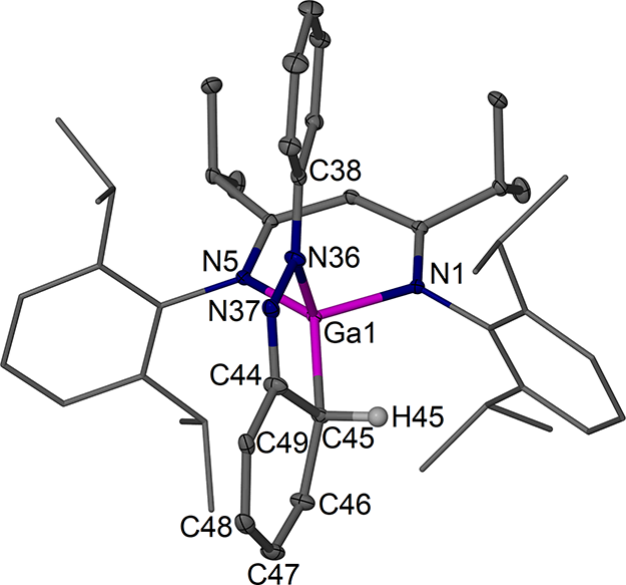
Molecular structure (30% thermal ellipsoids) of [(^*i*PrDip^nacnac)­Ga­{(C_6_H_5_)­NNPh}]·0.5 C_6_H_6_, **4b**·0.5 C_6_H_6_. Hydrogen atoms, except for H45, minor components of disorder, and the solvent molecule are omitted for clarity. Dip groups are shown as wireframe. Selected bond lengths (Å) and angles (°): Ga1–N1 1.9424(12), Ga1–N5 1.9519(12), Ga1–N36 1.9323(13), Ga1–C45 2.021(2); N1–Ga1–N5 98.69(5), N1–Ga1–C45 113.45(9), N5–Ga1–C45 136.20(10), N36–Ga1–N1 111.13(5), N36–Ga1–N5 111.22(5), N36–Ga1–C45 84.85(7), N37–N36–C38 115.76(12), C44–N37–N36 113.54(13), N37–C44–C45 121.94(15). Further distances are collected in Figure 7.

**Figure 7:**
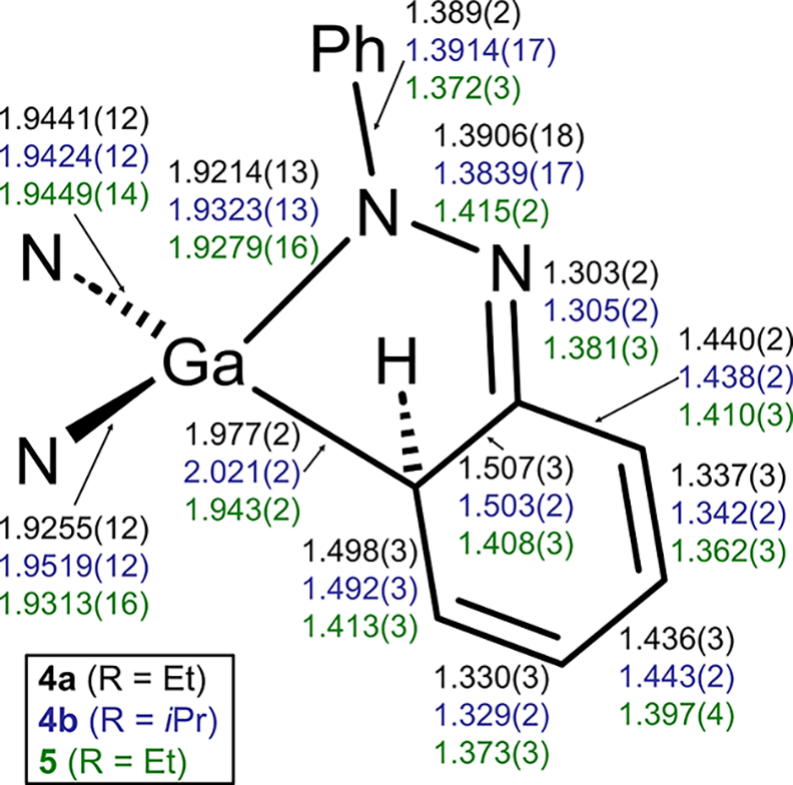
Bond length comparisons of the converted azobenzene moiety in **4a** (top values, black), **4b** (middle values, blue), and **5** (bottom values, green).

The ^1^H NMR spectrum of **4a** is shown in Figure 8 with some assignment of the resonances (numbers 1–15) and the corresponding spectrum for **4b** is highly comparable (Figure S14). The spectrum shows resonances for one ^EtDip^nacnac environment, for example, with one backbone CH singlet at 5.37 ppm (resonance 9), where all Dip isopropyl hydrogens are inequivalent, providing four septets (12) and eight partially overlapping doublets (13), highlighting that the chemical environment is different above and below the ^EtDip^nacnac plane, and near the two sides of the ligand at the Dip substituents. The latter can also be inferred from the inequivalence of the two ethyl groups (2 triplets at 11, and a multiplet from overlapping doublet of quartets at 10). Resonances for the reduced and dearomatized former phenyl group (1–5) are shifted significantly upfield, especially the hydrogen (1) on the gallium-bound carbon center. The phenyl group on the former azobenzene fragment shows five distinct, predominantly broad ^1^H NMR resonances (6–8) suggesting that its “frozen” position perpendicular to the ^EtDip^nacnac plane between the two Dip substituents is retained in solution. We tentatively assign the phenyl ortho-C–H contact to the nacnac backbone, vide supra, to the upfield position of 6, whereas the most downfield resonance (6) is assigned to the opposite ortho-C–H atom of the phenyl group near the N atom (Figure 8).

**Figure 8:**
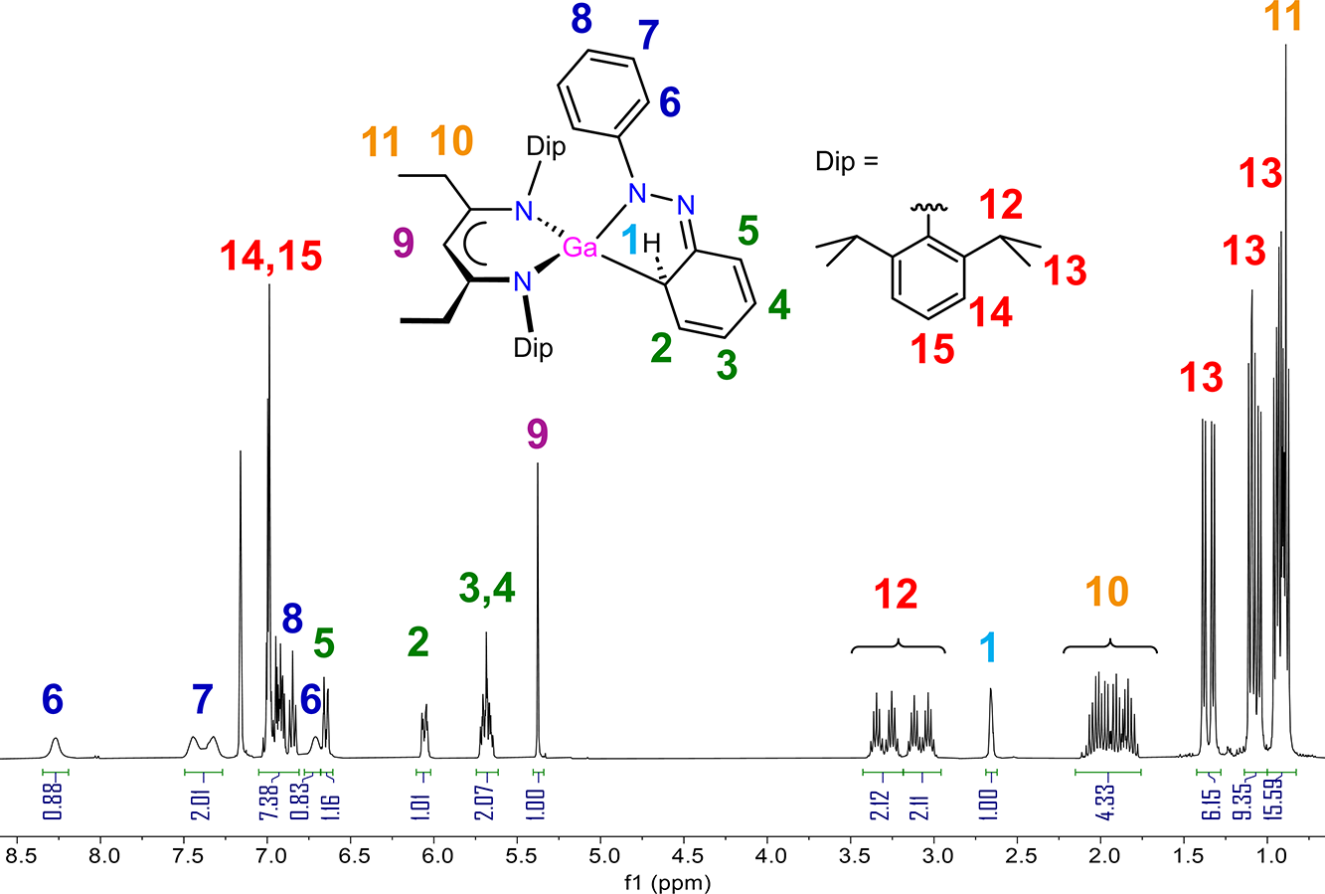
^1^H NMR spectrum (excerpt) of [(^EtDip^nacnac)­Ga­{(C_6_H_5_)­NNPh}] **4a** and partial resonance assignment.

To test the stability of the reduced azobenzene complexes, the more readily accessible [(^EtDip^nacnac)­Ga­{(C_6_H_5_)­NNPh}] **4a** was heated in deuterated benzene to 100 °C for 1 week, see Scheme 3. We anticipated that a more stable, rearomatized isomer complex **5** would form, c.f. complex **V**, but **4a** was found to be stable under these conditions. With the aim of further derivatizing the reduced azobenzene fragment, ideally building on the dearomatized aryl substituent, we treated **4a** with 1 equiv of benzophenone (Scheme 3) for 2 days at 80 °C in deuterated benzene. The changes in NMR resonances suggested the formation of a more symmetric complex without incorporation of the benzophenone moiety. Resonances for the same new complex formed when **4a** was treated with the alkenes 2,3-dimethyl-1,3-butadiene and cyclohexadiene, over the course of 2–4 days at 80 °C. Spectra of the mixtures suggested that, again, the alkene precursors were not incorporated into the product. The light brownish crystalline tautomer complex [(^EtDip^nacnac)­Ga­{(C_6_H_4_)­N­(H)­NPh}] **5**, see Scheme 3 and Figure 9, was isolated and structurally characterized and is believed to be the thermodynamic product isomer of **4a**. The heating experiment of **4a** suggests that a significant kinetic barrier for interconversion between **4a** and **5** is present. It is currently not known how the ketone- or alkene-promoted conversions proceed mechanistically, but no obvious intermediates were observed when the reactions were monitored by ^1^H NMR spectroscopy at intervals. Complex **VII** isomerized to an analogous tautomer of **5** upon addition of acetonitrile.^35^ The molecular structure of **5** shows that the reduced azobenzene unit is essentially planar, the ring system has rearomatized, and that a hydrogen atom migrated from the ortho-C–H-activated position to a nitrogen center. Key bond lengths of **5** are added to Figure 7 to allow comparison and show a range of expected bond lengths for the aryl C–C-bonds in the C–H-activated aryl group and single bond character for the N–N bond and N–C bonds. The ^1^H NMR spectrum of **5** corroborates these findings and shows resonances for aromatic groups and one ^EtDip^nacnac ligand environment with two septets and four doublets for the *i*Pr-Dip hydrogens that would be expected for a complex with a different environment above and below the β-diketiminate plane.

**Scheme 3:**
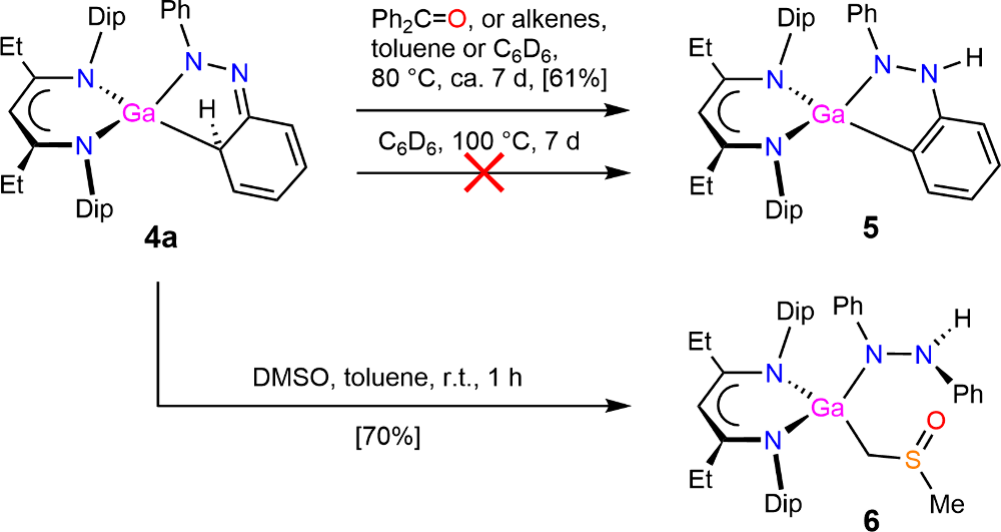
Synthesis of Compounds **5** and **6**

**Figure 9:**
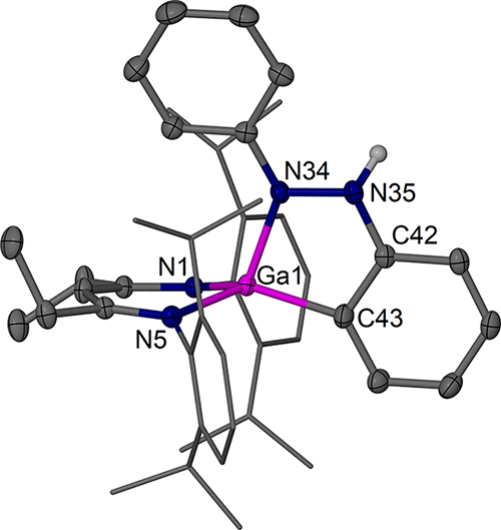
Molecular structure (30% thermal ellipsoids) of [(^EtDip^nacnac)­Ga­{(C_6_H_4_)­N­(H)­NPh}] **5**. Hydrogen atoms, except for H35, are omitted for clarity, and Dip groups are shown as wireframe. Selected bond lengths (Å) and angles (°): Ga1–N1 1.9448(14), Ga1–N5 1.9313(16), Ga1–N34 1.9279(16), Ga1–C43 1.943(2); N5–Ga1–N1 96.85(6), N5–Ga1–C43 124.30(9), N34–Ga1–N1 112.48(7), N34–Ga1–N5 113.11(7), N34–Ga1–C43 87.26(8), C43–Ga1–N1 123.34(8), C36–N34–N35 114.98(16), C42–N35–N34 114.03(16), N35–C42–C43 120.11(18). Further distances are collected in Figure 7.

Treating complex **4a** with dimethyl sulfoxide (DMSO) resulted in the formation of [(^EtDip^nacnac)­Ga­(PhNNHPh)­(CH_2_S­(O)­Me)] **6** where the reduced azobenzene moiety converted to a 1,2-diphenylhydrazide ligand, and a DMSO molecule is deprotonated and coordinates to the Ga center via a deprotonated methyl group and not via the oxygen atom, see Scheme 3 and Figure 10. To gain further insights into this reaction, a frozen deuterated benzene solution of **4a** was treated with DMSO and upon thawing and mixing, and the reaction was immediately followed by ^1^H NMR spectroscopy. At the first point of analysis after approximately 1–2 min, all **4a** had been consumed, more than 90% of **6** had formed, and no further changes were observed subsequently. The Ga-CH_2_S­(O)­Me fragment was recently observed by the Nikonov group in activation chemistry of (^MeDip^nacnac)Ga complexes,^45–47^ for example, from activation of an in situ formed gallium oxide species or by activating a gallium­(I) complex with phenyl isocyanate.^45,47^ The deprotonation of DMSO is significant, as its p*K*_a_ value was estimated to be about 35 and shows the basicity of the ortho-metalated phenyl group.^48^

**Figure 10:**
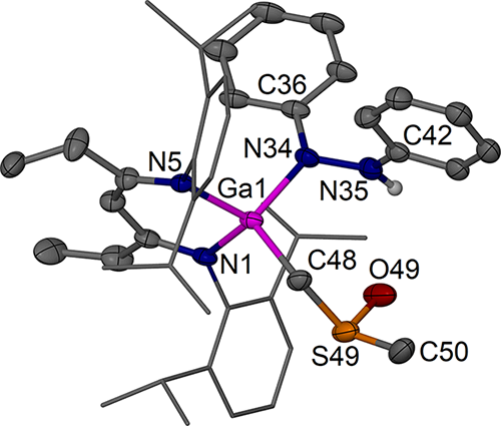
Molecular structure (30% thermal ellipsoids) of [(^EtDip^nacnac)­Ga­(PhNNHPh)­(CH_2_S­(O)­Me)]·OSMe_2_, **6**·OSMe_2_. Only one of the two independent molecules is shown. Hydrogen atoms, except for H35, solvent molecules, and minor components of disorder are omitted for clarity. Dip groups are shown as wireframe. Selected bond lengths (Å) and angles (°): Ga(1)–N(1) 1.937(5), Ga(1)–N(5) 1.980(5), Ga(1)–N(34) 1.907(5), Ga(1)–C(48) 1.983(7), O(49)–S(49) 1.496(6), S(49)–C(50) 1.759(12), N(34)–N(35) 1.418(8); N(1)–Ga(1)–N(5) 97.5(2), N(1)–Ga(1)–C(48) 113.8(3), N(1)–Ga(1)–N(34) 115.1(2), N34–Ga1–N5 109.6(2), N34–Ga1–C48 106.8(3), Ga(1)–C(48)–S(49) 115.5(4), Ga(1)–N(34)–N(35), 117.2­(4), and O(49)–S(49)–C(50) 104.1(5).

The molecular structure of complex **6** (Figure 10) generally shows multiple signs of minor disorder as well as lower than ideal bond precision, but the bond lengths in the gallium-bound hydrazide moiety and the deprotonated DMSO ligand show the expected distances and geometries in both independent molecules in the asymmetric unit. The Ga center in **6** is coordinated in a distorted tetrahedral manner, with one CH_2_S­(O)­Me ligand and a κ^1^-1,2-diphenylhydrazide ligand showing expected bond lengths. The torsion angles of the PhNNPh unit are significantly larger than 90° (ca. 108 and 114°), and the nitrogen atoms show a distorted trigonal planar geometry. An S=O····HN hydrogen bond (2.38 Å mean) locks the ligands in place, and the *N*-phenyl group sits orthogonal to the nacnac plane between the Dip substituents in a way that a short ortho-C–H to nacnac backbone C­(H) contact (2.72 Å mean) can form, cf. the structures of **4a** and **4b**. The ^1^H NMR spectrum of **6** suggests a highly asymmetric ^EtDip^nacnac environment for the resonances of the *i*Pr-Dip hydrogens (c.f. **4a** and **4b**) and the GaCH_2_S resonances, which were found as two doublets (^2^ *J*_HH_ ≈ 12 Hz) at δ 1.52 and 1.99 ppm with geminal coupling between them and the S­(O)­CH_3_ singlet at δ 1.63 ppm. These features are similar to those found in the examples by Nikonov et al.^45–47^ A sharp downfield singlet at δ 8.90 ppm is assigned to the hydrogen-bonded NH group.

To further target reactivity that employs the dearomatized phenyl group from the former azobenzene fragment, complex **4a** was treated with benzaldehyde, which converted within 3 h at 50 °C to a bright yellow solution. After removal of all volatiles and extracting the residue into *n*-hexane, large yellow crystals of [(^EtDip^nacnac)­Ga­(PhNN­(Ph)­CH­(Ph)­O)] **7** were afforded at −40 °C, see Scheme 4 and Figure 11. In the molecular structure of complex **7**, a new five-membered GaN­(Ph)­N­(Ph)­CH­(Ph)O chelate ring is formed by the nucleophilic addition of a nitrogen atom to the benzaldehyde carbonyl center again rearomatizing the formerly activated phenyl group. The N–N bond length (1.453(2) Å) is consistent with a single bond. The N­(Ph) atom (N34) that coordinates to the Ga center shows a trigonal planar geometry (sum of angles: 356.5(4)°), whereas the N­(Ph) atoms (N35) that is bonded to the former benzaldehyde carbonyl group (C36) is trigonal pyramidal (sum of angles: 330.7(4)°). This difference also translates to the N–C­(Ph) distances: that for N34 (1.383(2) Å) is significantly shorter than that for N35 (1.453(2) Å) and supports that the N atom with trigonal planar coordination delocalizes anionic charge onto the phenyl ring, which leads to bond shortening. The neighboring phenyl groups on N35 and C36 are on the same side of the ring in a cis-position relative to each other. To the best of our knowledge, there is only one reported metal complex with this chelate ring system, PhNNPCHPhO, in a Mg complex where the crystal structure only is deposited.^49^ In this species, the neighboring phenyl groups on N and C are positioned trans relative to each other, likely for steric reasons. The ^1^H NMR spectrum of **7** again shows the anticipated highly asymmetric ^EtDip^nacnac environment for the resonances of the *i*Pr-Dip hydrogens (c.f. **4a**, **4b**, and **6**) due to the chiral chelate ring. The H­(C) hydrogen of the former benzaldehyde unit gives a broad singlet at δ 5.62 ppm in the ^1^H NMR spectrum.

**Scheme 4:**
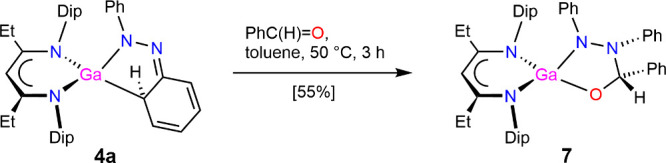
Synthesis of Compound **7**

**Figure 11:**
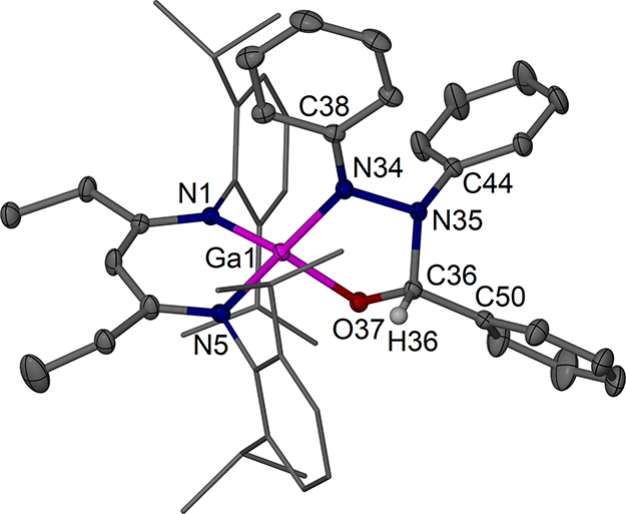
Molecular structure (30% thermal ellipsoids) of [(^EtDip^nacnac)­Ga­(PhNN­(Ph)­CH­(Ph)­O)]·0.5 C_5_H_12_, **7**·0.5 C_5_H_12_. Only one of two independent molecules is shown. Hydrogen atoms, except for H36, a second main molecule, solvent molecules, and minor components of disorder are omitted for clarity. Dip groups are shown as wireframe. Selected bond lengths (Å) and angles (°): Ga1–O37 1.8325(12), Ga1–N1 1.9114(14), Ga1–N5 1.9221(15), Ga1–N34 1.8801(15), O37–C36 1.415(2), N34–N35 1.453(2), N34–C38 1.383(2), N35–C36 1.489(2), N35–C44 1.453(2); O37–Ga1–N1 120.08(6), O37–Ga1–N5 110.64(6), O37–Ga1–N34 89.46(6), N1–Ga1–N5 98.61(6), N34–Ga1–N1 120.91(6), N34–Ga1–N5 118.29(7), C36–O37–Ga1 103.70(10), N35–N34–Ga1 109.85(11), N34–N35–C36 104.66(13), O37–C36–N35 111.59(14).

We furthermore targeted mixed-metal low-oxidation-state group 13 hydride complexes^50^ incorporating the [(^EtDip^nacnac)­Ga] system. We treated [(^EtDip^nacnac)­Ga] **2a** with aluminum­(III) hydride complexes in analogy to related reactions of β-diketiminate aluminum­(I) and gallium­(I) complexes with metal hydrides to form group 13 metal–metal-bonded hydride species.^51–57^ The reaction of (NHC)­AlH_3_ (NHC = {MeCN­(*i*Pr)}_2_C)^58^ with **2a** proceeded over 16 h at 60 °C or at room temperature for 2 days to afford yellow [(^EtDip^nacnac)­Ga­(H)–Al­(H_2_)­(NHC)] **8**, see Scheme 5 and Figure 12, in good in situ yield. Complex **8** is a formal Al–H bond insertion product from “oxidative” addition^8^ of an aluminum­(III) hydride complex to the gallium­(I) center in **2a** if one considers the Ga–Al bond electron-sharing with Ga^II^ and Al^II^ centers. Alternatively, the formal oxidation states can be considered unchanged as Ga^I^ and Al^III^, i.e, by resulting from hydride addition to **2a** plus stabilization of the resulting -ate complex with an (NHC)­AlH_2_^+^ fragment.

**Scheme 5:**
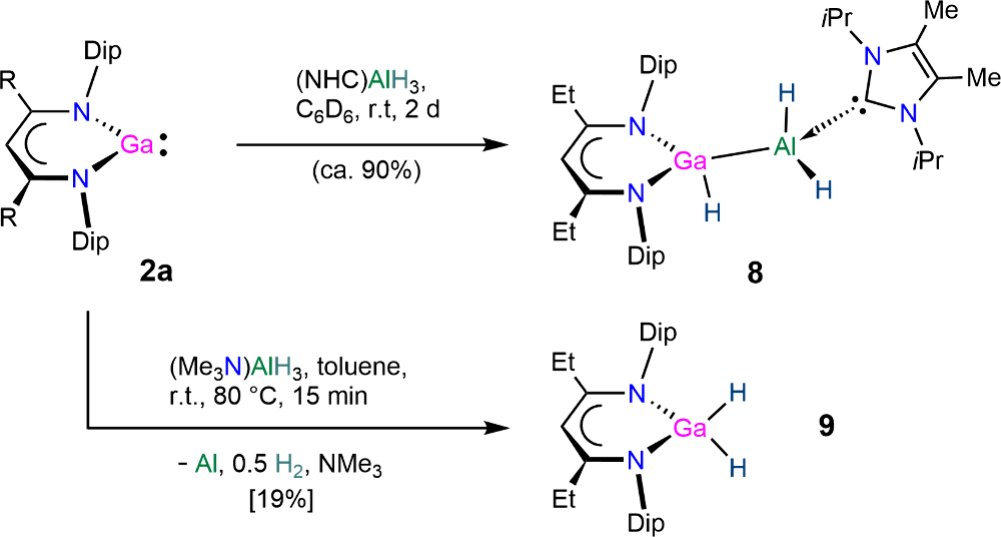
Synthesis of Compounds **8** and **9.**

**Figure 12:**
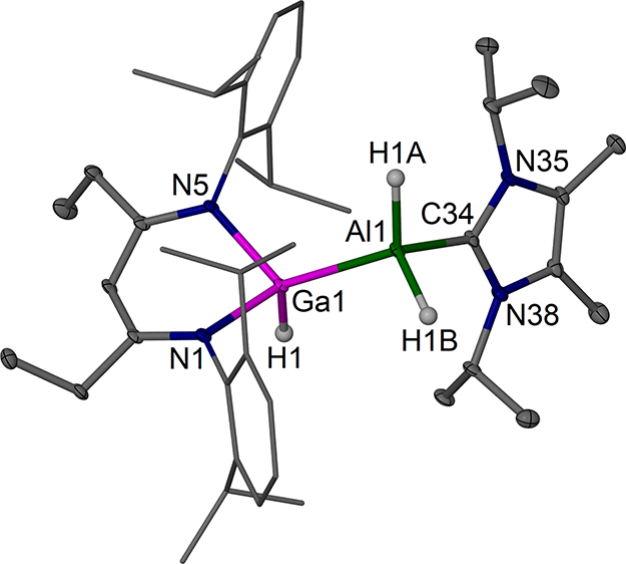
Molecular structure (30% thermal ellipsoids) of [(^EtDip^nacnac)­Ga­(H)–Al­(H_2_)­(NHC)]·0.5 C_6_H_14_, **8**·0.5 C_6_H_14_. Hydrogen atoms, except for hydride ligands, and a solvent molecule are omitted for clarity. Dip groups are shown as wireframe. Selected bond lengths (Å) and angles (°): Ga1–Al1 2.5072(8), Ga(1)–N(1) 2.013(2), Ga(1)–N(5) 2.014(2), Al(1)–C(34) 2.069(3), Ga1–H1 1.58(4), Al1–H1A 1.56(4), Al1–H1B 1.52(4); N1–Ga1–Al1 118.50(7), N5–Ga1–Al1 119.40(7), C34–Al1–Ga1 104.82(8), N1–Ga1–N5 91.88(9).

In **8**, the Ga center sits significantly above the chelating ^EtDip^nacnac plane (ca. 0.76 Å) in a distorted tetrahedral coordination environment binding to one hydride ligand and the AlH_2_(NHC) moiety. The structure is geometrically similar to that of [(^MeDip^nacnac)­Al­(H)–Al­(H_2_)­(NHC′)] (NHC′ = {MeCN­(Cy)}_2_C, Cy = cyclohexyl).^56^ The majority of characterized complexes with Ga–Al interactions are dominated by donor–acceptor-type interactions, and, to the best of our knowledge, only one complex, [(^MeDip^nacnac)­Ga­(H)–Al­(H)­(TMEDA)]^+^,^53^ with a formal electron-sharing Ga–Al bond is known, which was prepared in a similar manner from a gallium­(I) complex and an Al–H complex. The Ga–Al bond length in **8** is 2.5072(8), highly similar to that in [(^MeDip^nacnac)­Ga­(H)–Al­(H)­(TMEDA)]^+^ (2.5238(9) Å),^53^ but these Ga–Al bonds are shorter than the Al–Al bond (2.5860(7) Å) in the related [(^MeDip^nacnac)­Al­(H)–Al­(H_2_)­(NHC′)].^56^

The ^1^H NMR spectrum of **8** shows one ^EtDip^nacnac-CH backbone singlet and a broad singlet for the Ga–H unit (δ = 6.20 ppm) and resonances from ^EtDip^nacnac-*i*Pr substituents that suggest a different average environment above and below the ^EtDip^nacnacGa plane in solution. The hydride bands in the IR spectrum provide a small sharp band (1753 cm^–1^) assigned to the terminal Ga–H bond and a strong not further resolved broad band centered around 1709 cm^–1^ for the AlH_2_ unit. These “redshifted” IR bands, c.f. complex **9**, vide infra, are further support for fragments with NHC-stabilized low-oxidation-state M–H moieties.^53,56,59^

The reaction of [(^EtDip^nacnac)­Ga] **2a** with (Me_3_N)­AlH_3_ was also investigated. Here, we propose that the analogous complex to **8**, [(^EtDip^nacnac)­Ga­(H)–Al­(H_2_)­(NMe_3_)], is formed as an intermediate, but upon trying to isolate the product by crystallization or precipitation, decomposition with metal formation occurred and the gallium­(III) hydride complex [(^EtDip^nacnac)­GaH_2_] **9**, c.f. [(^MeDip^nacnac)­GaH_2_],^60^ was afforded, see Scheme 5 and Figure 13. Thus, the reaction was repeated with an additional heating step at 80 °C to deliberately ensure full decomposition of the intermediate to **9**, and the complex was isolated in 19% crystallized yield and characterized. The isolation of complex **9** furthermore supports that aluminum metal is formed during the reaction and not gallium metal. For comparison, [(^EtDip^nacnac)­AlH_2_] is known as a rare complex of this ligand and is spectroscopically different from **9**.^61^ The molecular structure of **9** is comparable to related [(^RAr^nacnac)­GaH_2_] complexes.^60,62,63^ The ^1^H NMR spectrum of **9** shows sharp resonances of symmetry comparable to that of **2a**, plus a broad resonance at δ 5.20 ppm for the GaH_2_ unit, and an IR spectrum of **9** shows two stretching bands (1863 and 1892 cm^–1^) for the Ga–H bonds.

**Figure 13:**
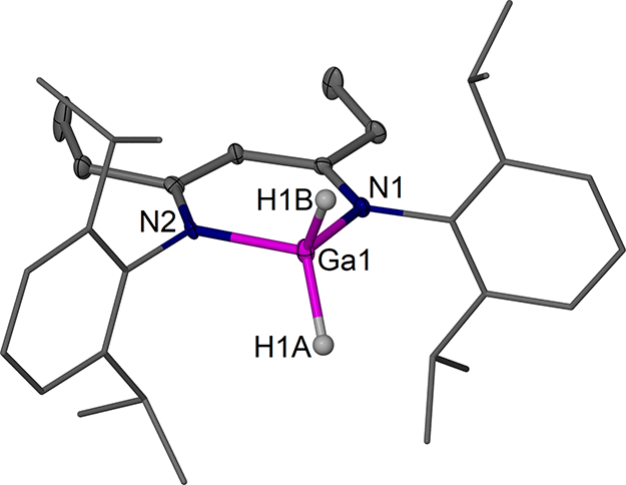
Molecular structure (30% thermal ellipsoids) of [(^EtDip^nacnac)­GaH_2_] **9**. Only one independent molecule is shown. Hydrogen atoms, except for hydride ligands, have been omitted for clarity. Dip groups are shown as wireframe. Selected bond lengths (Å) and angles (°): Ga1–N1 1.964(7), Ga1–N5 1.964(6), Ga1–H1A 1.55(8), Ga1–H1B 1.49(8); N1–Ga1–N5 95.0(3), H1A–Ga1–H1B 108(4).

## Conclusions

We have prepared the new gallium­(I) complexes [(^RDip^nacnac)­Ga], R = Et **2a**, and R = *i*Pr **2b** and found that the ligand backbone modification has a huge impact on the reaction yield. Complex **2a** is afforded in much higher yield than **2b**, which produced a lot of proligand ^iPrDip^nacnacH alongside. The high-yielding access to well-soluble **2a** and straightforward synthesis of its parent proligand^38^ make it a good alternative to commonly used [(^MeDip^nacnac)­Ga], whereas **2b** showed surprising downsides (low yield, poorer solubility). Both **2a** and **2b** could be converted with azobenzene to the partially dearomatized complexes [(^RDip^nacnac)­Ga­{(C_6_H_5_)­NNPh}] **4a** and **4b**. Complex **4a** is thermally stable in solution and only converted to the rearomatized product [(^EtDip^nacnac)­Ga­{(C_6_H_4_)­N­(H)­NPh}] **5** after treatment with benzophenone or some alkenes at elevated temperatures. These observations suggest that **5** is lower in energy than **4a**, but there is a considerable activation barrier for the conversion of **4a** to **5**. Treating **4a** with DMSO led to the synthesis of [(^EtDip^nacnac)­Ga­(PhNNHPh)­(CH_2_S­(O)­Me)] **6** featuring a Ga–C bond, and the reaction of **4a** with benzaldehyde furnished [(^EtDip^nacnac)­Ga­(PhNN­(Ph)­CH­(Ph)­O)] **7** with a newly formed N–C bond in the new chelating ring. Although the dearomatized **4a** and **4b** are readily accessible in good yield, follow-on reactivity has led to derivatization products (**5–** **7**), which did allow to build on the activated azobenzene fragment, e.g., in **7**. However, all the products afforded so far show a rearomatized aryl substituent, rather than allowing further derivatization of the activated phenyl group to more saturated heterocycle products. In addition, we have treated **2a** with the aluminum trihydride complexes (Do)­AlH_3_, Do = NHC (= {MeCN­(*i*Pr)}_2_C), NMe_3_, which afforded the Ga–Al-bonded complex [(^EtDip^nacnac)­Ga­(H)–Al­(H_2_)­(NHC)] **8** and the gallium­(III) hydride complex [(^EtDip^nacnac)­GaH_2_] **9** after decomposition of the proposed intermediate [(^EtDip^nacnac)­Ga­(H)–Al­(H_2_)­(NMe_3_)].

## Supplementary Material



## Data Availability

The research data (NMR spectroscopy) supporting this publication can be accessed at 10.17630/b5a871be-8160-4653-9280-6d65e1be7492

## Notes

Experimental section, NMR spectra, and X-ray crystallography (PDF)
