# Supplementary material for: Azobenzene Reduction and Derivatization and Al–H Bond Insertion with β‑Diketiminate Gallium(I) Complexes
Source: Organometallics. 2026 Feb 25;45(6):711–20. doi: 10.1021/acs.organomet.6c00019 (PMC13014532; doi:10.1021/acs.organomet.6c00019)
Supplement: Supplementary file 1 [file om6c00019_si_001.pdf]

Supporting Information

for

Azobenzene reduction and derivatisation, and Al–H bond insertion with β-diketiminate gallium(I) complexes

*Huanhuan Dong, Connor Bourne, Aidan P. McKay, Alexandra M. Z. Slawin, David B. Cordes and Andreas Stasch**

EaStCHEM School of Chemistry, University of St Andrews, North Haugh, St Andrews, KY16 9ST, United Kingdom. E-mail: as411@st-andrews.ac.uk.

Table of Contents

1 Experimental Section S2
2 NMR spectroscopy S12
3 X-ray crystallography S48
4 References S53

1 Experimental Section

1.1 General considerations

All manipulations were carried out using standard Schlenk and glove box techniques under an atmosphere of high purity argon or dinitrogen. Benzene, toluene, *n*-hexane and *n*-pentane were either dried and distilled under inert gas over $LiAlH_4$, sodium or potassium, or taken from an MBraun solvent purification system and degassed prior to use. ^{1}H and $^{13}C\{^{1}H\}$ NMR spectra were recorded on a Bruker AVII 400 or Bruker AV III 500 spectrometer in deuterated benzene or toluene and were referenced to the residual ^{1}H or $^{13}C\{^{1}H\}$ resonances of the solvent used. Abbreviations: s = singlet, d = doublet, t = triplet, q = quartet, sept = septet, br = broad, m = multiplet. Yields or conversions in solution were determined by integration of ^{1}H NMR spectra against an internal standard (such as residual C_6D_5H from deuterated benzene, added hexamethylbenzene etc). IR (ATR) spectra were obtained on a Shimadzu IR Affinity spectrometer. Melting points were determined in sealed glass capillaries under argon and are uncorrected. Elemental analyses were performed by the Elemental Analysis Service at London Metropolitan University. The syntheses of ^{EtDip}nacnacH, [(^{EtDip}nacnac)Li], ^{iPrDip}nacnacH, [(^{iPrDip}nacnac)K] were performed according to a reported procedures.[1] “GaI”,[2] benzylpotassium,[3] potassium graphite (KC_8),[4] (NHC)AlH_3 (NHC = {MeCN(*i*Pr)}$_2$C),[5] and (Me_3N)AlH_3[6] were prepared according to literature procedures. All other compounds were used as received from chemical suppliers.

Selected NMR spectra are collected in Section 2. Unless stated otherwise, NMR spectra were recorded in deuterated benzene. Chemical shifts are given in ppm. Further information has been provided in the Figure captions.

1.2 Syntheses

Synthesis of [(^{EtDip}nacnac)Ga] 2a

The lithium complex [(^{EtDip}nacnac)Li] **1a** was firstly prepared by adding *n*BuLi (1.6 M in *n*-hexane, 1.93 mL, 3.08 mmol, 1.1 equiv.) into a toluene (25 mL) solution of ^{EtDip}nacnacH (1.25 g, 2.80 mmol, 1.0 equiv.) at -78 °C. The reaction mixture was allowed to stir and warm to room temperate overnight to yield a pale-yellow solution. The solution of [(^{EtDip}nacnac)Li] **1a** (1.20 g, 2.65 mmol, 1.0 equiv.) in toluene (25 mL) was then added into the freshly made "GaI" (678 mg, 3.45 mmol, 1.3 equiv.) suspension in toluene (20 mL) at 0 °C. The resulting yellowish reaction mixture was then warmed to ambient temperature and vigorously stirred overnight.(*) Then, all volatiles were removed under reduced pressure and *n*-hexane (20 mL) was added, which resulted in the formation of a light-yellow precipitate. This was then settled, filtered, and dried *in vacuo* to give crude product of [(^{EtDip}nacnac)Ga] **2a** as a yellow powder. (The compound could be recrystallised from toluene) Note: The second crop of crystalline solid was obtained after storing the hexane filtrate at -40 °C overnight, which was isolated and analysed by single crystal X-ray diffraction to be [(^{EtDip}nacnac)GaI_2] **3a**.
Yield (of **2a**): 658 mg (48%). (*)With an additional KC_8 addition step: KC_8 (265 mg, 1.96 mmol, 0.7 equiv.) was added via a solid-addition flask to the reaction mixture, and the resulting mixture was further stirred at room temperature for 2 h, followed by the described work-up. Yield (of **2a**): 982 mg (68%). Mp.: 243-245 °C (melts). ^{1}H NMR (499.9 MHz, benzene-d_6, 298 K) δ = 0.98 (t, J_{HH} = 7.6 Hz, 6H, Et-CH_3)), 1.18 (d, J_{HH} = 6.9 Hz, 12H, Ar-*o*-CH(CH_3)$_2$), 1.33 (d, J_{HH} = 6.9 Hz, 12H, Ar-*o*-CH(CH_3)$_2$), 2.16 (q, J_{HH} = 7.6 Hz, 4H, Et-CH_2), 3.16 (sept, J_{HH} = 6.9 Hz, 4H, Ar-*o*-C*H*(CH$_3$)$_2$), 5.40 (s, 1H, NC(Et)C*H*), 7.18 (s, 6H, Ar-H). ^{13}C{^{1}H} NMR (125.7 MHz, benzene-d_6, 298 K): δ = 13.0 (Et-*C*H$_3$). 23.8 (Ar-*o*-CH(*C*H$_3$)$_2$), 26.1 (Ar-*o*-CH(*C*H$_3$)$_2$), 28.7 (Ar-*o*-*C*H(CH$_3$)$_2$), 29.3 (Et-*C*H$_2$), 94.1 (NC(Et)*C*H), 124.2 (Ar-*C*), 126.7 (Ar-*C*), 143.5 (Ar-*C*), 143.6 (Ar-*C*), 168.6 (N*C*(Et)). IR (ATR), v (cm^{-1}): 2961 (m), 2866 (m), 1528 (s), 1314 (s), 1261 (s), 1070 (m), 789 (s), 754 (s). Elemental analysis: calculated for $C_{31}H_{45}GaN_2$: C 72.20; H 8.80; N 5.43%; found: C 72.03; H 8.81; N 5.24%.
Data for [(^{EtDip}nacnac)GaI_2] **3a**: ^{1}H NMR (499.9 MHz, benzene-d_6, 298 K) δ = 0.82 (t, J_{HH} = 7.5 Hz, 6H, Et-CH_3). 1.12 (d, J_{HH} = 6.8 Hz, 12H, Ar-*o*-CH(CH_3)$_2$), 1.46 (d, J_{HH} = 6.7 Hz, 12H, Ar-*o*-CH(CH_3)$_2$), 1.96 (q, J_{HH} = 7.5 Hz, 4H, Et-CH_2), 3.58 (sept, J_{HH} = 6.7 Hz, 4H, Ar-*o*-C*H*(CH$_3$)$_2$), 5.28 (s, 1H, NC(Et)C*H*), 7.13 (s, 6H, Ar-H), ^{13}C{^{1}H} NMR (125.7 MHz, benzene-d_6, 298 K): δ = 12.2 (Et-*C*H$_3$), 24.5 (Ar-*o*-CH(*C*H$_3$)$_2$), 26.7 (Ar-*o*-CH(*C*H$_3$)$_2$), 28.9 (Ar-*o*-*C*H(CH$_3$)$_2$), 29.0 (Et-*C*H$_2$), 93.6 (NC(Et)*C*H), 124.9 (Ar-*C*), 138.7 (Ar-*C*), 145.1 (Ar-*C*), 175.8 (N*C*(Et)). IR (ATR), v (cm^{-1}): 2972 (m), 2930 (m), 2868 (m), 1587 (s), 1522 (s), 1404 (s), 1252 (s), 1007 (m), 746 (s).

[(^{EtDip}nacnac)GaI_2] 3a

Complex **3a** can also be independently synthesised: toluene (30 mL) was added to a Schlenk flask charged with [(^{EtDip}nacnac)Li] **1a** (1.00 g, 2.20 mmol, 1.0 equiv.) and GaI_3 (1.14 g, 2.52 mmol, 1.15 equiv.) at 0 °C and the reaction mixture was stirred at room temperature overnight. The resulting cloudy yellow solution was settled, filtered before all the volatiles from the filtrate were removed under vacuum and the residue extracted with *n*-hexane (20 mL). The residual solid was dried *in vacuo* and collected as crude product of **3a**. The *n*-hexane solution was stored at -40 °C overnight afforded large colourless crystals of **3a**. Yield: 680 mg (70 %). Mp.: 254-255 °C. For spectroscopic data, see above.

Synthesis of [(^{iPrDip}nacnac)Ga] 2b

Toluene (30 mL) was added to a Schlenk flask charged with ^{iPrDip}nacnacH (1.00 g, 2.10 mmol, 1.0 equiv.) and benzyl potassium (302 mg, 2.30 mmol, 1.1 equiv.). The resulting mixture was stirred at room temperature overnight (*ca.* 16 h) afforded a toluene solution of [(^{iPrDip}nacnac)K], which was transferred to an *in-situ* generated slurry of "GaI" (332 mg, 2.10 mmol, 1.0 equiv.) in toluene (20 mL) at 0 °C. The reaction mixture was slowly warmed to room temperature and stirred overnight during which time precipitation of a white solid was observed. This was then allowed to settle before being filtered. Volatiles were removed from the filtrate *in vacuo* and *n*-hexane (20 mL) was added, which resulted in the formation of a yellowish precipitate (mainly containing ^{iPrDip}nacnacH). The mixture was then filtered, the hexane filtrate was concentrated to *ca.* 10 ml under reduced pressure and stored at -40 °C for two days which yielded a yellow precipitate, which was analysed to be compound **2b** with *ca.* 10% of ^{iPrDip}nacnacH according to ^{1}H NMR spectroscopy. Large yellow crystals suitable for single crystal X-ray diffraction analysis were acquired from a concentrated benzene-d_6 solution. Yield: 110 mg (10%). ^{1}H NMR (400.3 MHz, benzene-d_6, 296 K) δ = 1.03 (d, J_{HH} = 6.8 Hz, 12H, NC(CH(CH_3)$_2$)), 1.19 (d, J_{HH} = 6.9 Hz, 12H, Ar-*o*-CH(CH_3)$_2$), 1.35 (d, J_{HH} = 6.8 Hz, 12H, Ar-*o*-CH(CH_3)$_2$), 2.66 (sept, J_{HH} = 6.5 Hz, 2H, NC(C*H*(CH$_3$)$_2$)), 3.14 (sept, J_{HH} = 6.8 Hz, 4H, Ar-*o*-C*H*(CH$_3$)$_2$), 5.35 (s, 1H, NCC*H*CN), 7.15 (s, 6H, Ar-*H*). ^{13}C{^{1}H} NMR (125.7 MHz, benzene-d_6, 298 K): δ = 21.1 (NC(CH(*C*H$_3$)$_2$)), 22.5 (Ar-*o*-CH(*C*H$_3$)$_2$), 23.2 (Ar-*o*-CH(*C*H$_3$)$_2$), 26.0 (Ar-*o*-*C*H(CH$_3$)$_2$), 27.8 (NC(*C*H(CH$_3$)$_2$)), 123.7 (NC*C*HCN), 125.3 (Ar-*C*), 128.2 (Ar-*C*), 129.0 (Ar-*C*), 143.4 (Ar-*C*), 173.0 (N*C*(CH(CH$_3$)$_2$)). No further analysis was conducted due to the relatively low purity and low yield of **2b**.

Synthesis of [(^{iPrDip}nacnac)GaI$_2$] 3b

Toluene (30 mL) was added to a Schlenk flask charged with [(^{iPrDip}nacnac)Li] **1b** (1.00 g, 2.07 mmol, 1.0 equiv.) and GaI$_3$ (1.07 g, 2.38 mmol, 1.15 equiv.) at 0 °C and the reaction mixture was stirred at room temperature overnight. The resulting solution was settled, filtered before all the volatiles from the filtrate were removed *in vacuo* and the residue extracted with *n*-hexane (20 mL). The residual solid was dried under vacuum and collected as crude product of **3b**. The *n*-hexane solution was stored at -40 °C overnight and afforded colourless crystals of **3b**, which were suitable for single crystal X-ray diffraction analysis. Yield: 710 mg (62 %). Mp.: turned brownish at around 198 °C, melts at 249-250 °C. ^{1}H NMR (499.9 MHz, benzene-d_6, 298 K) 0.93 (d, J_{HH} = 6.6 Hz, 12H, NC(CH(C*H*$_3$)$_2$)), 1.19 (d, J_{HH} = 6.8 Hz, 12H, Ar-*o*-CH(C*H*$_3$)$_2$), 1.46 (d, J_{HH} = 6.6 Hz, 12H, Ar-*o*-CH(C*H*$_3$)$_2$), 2.44 (sept, J_{HH} = 6.6 Hz, 2H, NC(C*H*(CH$_3$)$_2$)), 3.58 (br, 4H, Ar-*o*-C*H*(CH$_3$)$_2$), 5.35 (s, 1H, NCC*H*CN), δ = 7.12 (t, J_{HH} = 2.8 Hz, 6H, Ar-*H*). ^{13}C{^{1}H} NMR (125.7 MHz, benzene-d_6, 295 K): δ = 22.5 (NC(CH(*C*H$_3$)$_2$)), 24.7 (Ar-*o*-CH(*C*H$_3$)$_2$), 27.8 (Ar-*o*-CH(*C*H$_3$)$_2$), 28.6 (Ar-*o*-*C*H(CH$_3$)$_2$), 32.1 (NC(*C*H(CH$_3$)$_2$)), 91.0 (NC*C*HCN), 125.0 (Ar-*C*), 138.2 (Ar-*C*), 145.7 (Ar-*C*), 181.2 (N*C*(CH(CH$_3$)$_2$)). IR (ATR), ν (cm^{-1}): 2963 (m), 2926 (m), 2866 (m), 1541 (s), 1508 (s), 1410 (s), 1258 (m), 1078 (m), 799 (s).

Synthesis of [(^{EtDip}nacnac)Ga{(C_6H_5)NNPh}] 4a

To a Schlenk flask charged with [(^{EtDip}nacnac)Ga] **2a** (100 mg, 193 µmol, 1.0 equiv.) and azobenzene (35.2 mg, 193 µmol, 1.0 equiv.) was added toluene (20 mL) and the reaction mixture was stirred at room temperature for three hours. All volatiles were removed under reduced pressure. The resulting residue was treated with *n*-hexane (10 mL), affording an orange precipitate, which was collected by filtration and dried *in vacuo* to give crude **4a**. The *n*-hexane solution was stored at -40 °C overnight and yielded yellow crystals (of **4a**) suitable for single crystal X-ray diffraction analysis. Yield: 108 mg (80%). Mp.: 244-245 °C. ^{1}H NMR (400.3 MHz, benzene-d_6, 298 K) δ = 0.88 (d, J_{HH} = 6.7 Hz, 3H, Ar-*o*-CH(CH_3)$_2$), 0.89 (d, J_{HH} = 6.7 Hz, 3H, Ar-*o*-CH(CH_3)$_2$), 0.90 (t, J_{HH} = 2.7 Hz, 3H, Et-CH_3), 0.94 (t, J_{HH} = 2.7 Hz, 3H, Et-CH_3), 0.95 (d, J_{HH} = 6.7 Hz, 3H, Ar-*o*-CH(CH_3)$_2$), 1.04 (d, J_{HH} = 6.7 Hz, 3H, Ar-*o*-CH(CH_3)$_2$), 1.08 (d, J_{HH} = 6.7 Hz, 3H, Ar-*o*-CH(CH_3)$_2$), 1.10 (d, J_{HH} = 6.7 Hz, 3H, Ar-*o*-CH(CH_3)$_2$), 1.32 (d, J_{HH} = 7.1 Hz, 3H, Ar-*o*-CH(CH_3)$_2$), 1.37 (d, J_{HH} = 6.9 Hz, 3H, Ar-*o*-CH(CH_3)$_2$), 1.75–2.10 (m, J_{HH} = 7.8 Hz, 4H, NC(CH_2CH$_3$)), 2.65 (br s, 1H, Ga-C*H*), 3.03 (sept, J_{HH} = 6.7 Hz, 1H, ArC*H*(CH$_3$)$_2$), 3.11 (sept, J_{HH} = 6.7 Hz, 1H, ArC*H*(CH$_3$)$_2$), 3.25 (sept, J_{HH} = 6.8 Hz, 1H, ArC*H*(CH$_3$)$_2$), 3.34 (sept, J_{HH} = 6.8 Hz, 1H, ArC*H*(CH$_3$)$_2$), 5.37 (s, 1H, NCC*H*CN), 5.61–5.74 (m, 2H, C_6H_5-C3-*H*, C4-*H*), 6.00–6.09 (m, 1H, C_6H_5-C2-*H*), 6.60–6.68 (m, 1H, C_6H_5-C5-*H*), 6.70 (br s, 1H, Ph-*H*), 6.84 (tt, J_{HH} = 7.2, 1H, Ph-*H*), 6.88–6.98 (m, 2H, Ar-*H*), 6.95–7.04 (m, 4H, Ar-H), 7.32 (br s, 1H, Ph-*H*), 7.44 (br s, 1H, Ph-*H*), 8.26 (br s, 1H, Ph-*H*). ^{13}C{^{1}H} NMR (100.7 MHz, benzene-d_6, 298 K): δ = 12.4 (Et-*C*H$_3$), 12.8 (Et-*C*H$_3$), 23.9 (Ar-*o*-CH(*C*H$_3$)$_2$), 24.0 (2 × Ar-*o*-CH(*C*H$_3$)$_2$), 24.2 (Ar-*o*-CH(*C*H$_3$)$_2$), 24.4 (Ar-*o*-CH(*C*H$_3$)$_2$), 24.5 (Ar-*o*-CH(*C*H$_3$)$_2$), 24.9 (Ar-*o*-CH(C*H*$_3$)$_2$), 25.2 (Ar-*o*-CH(*C*H$_3$)$_2$), 27.4 (2 × Ar-*o*-*C*H(CH$_3$)$_2$), 27.6 (Et-*C*H$_2$), 28.2 (Et-*C*H$_2$), 29.0 (Ar-*o*-CH(*C*H$_3$)$_2$), 29.1 (Ga-*C*H), 92.2 (NC*C*HCN), 115.6 (Ph-C), 116.8 (Ar-*C*H), 122.0 (*C*$_6$H$_5$), 123.3, 123.6 (Ar-*C*H), 123.7 (*C*$_6$H$_5$), 125.0 (Ar-*C*H), 125.2 (Ar-*C*H), 127.1 (*C*$_6$H$_5$), 127.5 (Ph-*C*), 127.5 (Ar-*C*H), 127.7, 127.9, 129.8 (Ph-*C*), 130.2 (*C*$_6$H$_5$), 139.1 (Ar-*C*), 139.6 (Ar-*C*), 141.9 (Ar-*C*), 142.3 (Ar-*C*), 145.9 (Ar-*C*), 146.2 (Ar-*C*), 148.2 (Ar-*C*), 151.7 (Ar-*C*), 174.2 (Ar-*C*), 176.3 (Ar-*C*). IR (ATR), ν (cm^{-1}): 2966 (s), 1530 (w, C=N), 1404 (s), 1260 (s), 1015 (s, N-N), 797 (s). Elemental analysis: calculated for $C_{45}H_{63}GaN_4$: C 74.03; H 7.95; N 8.03%; found: C 73.80; H 8.05; N 7.76%.

Synthesis of [(^{iPrDip}nacnac)Ga{(C_6H_5)NNPh}] 4b

To a J. Young NMR tube charged with a solution of **2b** (15.0 mg, 27.5 µmol, 1.0 equiv.) in benzene-d_6 (0.5 mL) was added azobenzene (5.0 mg, 27.5 µmol, 1.0 equiv.) affording an orange solution immediately. The full conversion of **2b** and the formation of **4b** (ca. 90% *in-situ* NMR yield) were observed according to ^{1}H NMR spectroscopy. Orange crystals suitable for single crystal X-ray diffraction analysis were obtained from the concentrated benzene-d_6 solution after storage at 4 °C for two days. ^{1}H NMR (400.1 MHz, benzene-d_6, 295 K) δ = 0.88 (d, J_{HH} = 6.6 Hz, 3H, Ar-*o*-CH(C*H*$_3$)$_2$). 0.91 (d, J_{HH} = 6.7 Hz, 6H, NC(CH(C*H*$_3$)$_2$)), 0.93 (d, J_{HH} = 6.7 Hz, 12H, Ar-*o*-CH(C*H*$_3$)$_2$), 1.08 (d, J_{HH} = 6.7 Hz, 3H, Ar-*o*-CH(C*H*$_3$)$_2$), 1.12 (d, J_{HH} = 6.4 Hz, 6H, NC(CH(C*H*$_3$)$_2$)), 1.17 (d, J_{HH} = 6.4 Hz, 3H, Ar-*o*-CH(C*H*$_3$)$_2$), 1.19 (d, J_{HH} = 6.6 Hz, 3H, Ar-*o*-CH(C*H*$_3$)$_2$), 1.30 (d, J_{HH} = 6.7 Hz, 3H, Ar-*o*-CH(C*H*$_3$)$_2$)), 1.36 (d, J_{HH} = 6.7 Hz, 3H, Ar-*o*-CH(C*H*$_3$)$_2$). 2.42 (sept, J_{HH} = 6.4 Hz, 1H, NC(C*H*(CH$_3$)$_2$)), 2.48 (sept, J_{HH} = 6.5 Hz, 1H, NC(C*H*(CH$_3$)$_2$)), 2.60 (br s, 1H, Ga-C*H*), 3.06 (sept, J_{HH} = 7.1 Hz, 1H, ArC$\underline{H}$(CH$_3$)$_2$), 3.10 (sept, J_{HH} = 6.5 Hz, 1H, ArC$\underline{H}$(CH$_3$)$_2$), 3.20 (sept, J_{HH} = 7.1 Hz, 1H, ArC$\underline{H}$(CH$_3$)$_2$), 3.28 (sept, J_{HH} = 6.9 Hz, 1H, ArC$\underline{H}$(CH$_3$)$_2$), 5.41 (s, 1H, NCC*H*CN), 5.66 (m, 1H, C$_6$*H*$_5$-C4) 5.72 (m, 1H, C$_6$*H*$_5$-C3), 6.03 (m, J_{HH} = 8.9 Hz, 1H, C$_6$*H*$_5$-C2), 6.68 (m, J_{HH} = 2.1 Hz, 1H, C$_6$*H*$_5$-C5), 6.70 (m, J_{HH} = 2.0 Hz, 1H, C$_6$*H*$_5$), 6.73 (s, 1H, Ph-*H*), 6.85 (tt, J_{HH} = 7.1, 1.1 Hz, 1H, Ar-*H*), 6.89–6.94 (m, 2H, Ar-*H*), 6.96–7.02 (m, 4H, Ar-*H*), 7.27 (br s, 1H, Ph-*H*), 7.46 (br s, 1H, Ph-*H*), 8.28 (br s, 1H, Ph-*H*). ^{13}C{^{1}H} NMR (100.7 MHz, benzene-d_6, 298 K): δ = 21.7 (NC(CH(*C*H$_3$)$_2$)), 22.0 (NC(CH(*C*H$_3$)$_2$)), 22.3 (NC(CH(*C*H$_3$)$_2$)), 22.9 (Ar-*o*-CH(*C*H$_3$)$_2$), 23.3 (NC(CH(*C*H$_3$)$_2$)), 24.4 (Ar-*o*-CH(*C*H$_3$)$_2$), 24.5 (Ar-*o*-CH(*C*H$_3$)$_2$), 24.7 (Ar-*o*-CH(*C*H$_3$)$_2$), 24.9 (Ar-*o*-CH(*C*H$_3$)$_2$)), 25.1 (Ar-*o*-CH(*C*H$_3$)$_2$), 25.2 (Ar-*o*-CH(*C*H$_3$)$_2$), 25.7 ((Ar-*o*-CH(*C*H$_3$)$_2$)), 27.4 (Ar-*o*-*C*H(CH$_3$)$_2$), 27.4 (Ar-*o*-*C*H(CH$_3$)$_2$), 27.9 (Ar-*o*-*C*H(CH$_3$)$_2$), 28.1 (Ar-*o*-*C*H(CH$_3$)$_2$), 28.4 (N*C*(CH(CH$_3$)$_2$)), 30.1, 31.3, 31.8 (N*C*(CH(CH$_3$)$_2$)), 89.3 (NC*C*HCN), 117.0 (Ph-*C*), 122.3 (Ar-*C*H), 123.2 (*C*$_6$H$_5$), 123.6 (Ar-*C*H), 123.6 (Ar-*C*H), 123.9 (*C*$_6$H$_5$), 125.0 (Ar-*C*H), 125.3 (Ar-*C*H), 127.5 (*C*$_6$H$_5$), 127.6 (Ph-*C*), 127.7 (Ar-*C*H), 127.8 (Ar-C), 127.9 (Ar-C), 128.9 (Ph-*C*), 130.8 (*C*$_6$H$_5$), 138.2 (Ar-*C*), 138.9 (Ar-*C*), 142.6 (Ar-*C*), 146.4 (Ar-*C*), 146.6 (Ar-*C*), 147.8 (Ar-*C*), 151.3 (Ar-*C*), 152.9 (Ar-*C*), 179.1 (Ar-*C*), 181.5 (Ar-*C*).

Synthesis of [(^{EtDip}nacnac)Ga{(C_6H_4)N(H)NPh}] 5

Method 1: To a J. Young NMR tube charged with a solution (0.5 mL) of [^{EtDip}nacnacGa{(C_6H_5)NNPh}] **4a** (10.0 mg, 14.3 µmol, 1.0 equiv.) in benzene-d_6 was added a stoichiometric amount of benzophenone (2.87 mg, 15.7 µmol, 1.1 equiv.). The solution mixture was heated at 100 °C for seven days until the integration of a newly formed compound no longer changed from the *in-situ* ^{1}H-NMR spectra (approximately 50% of **4a** was converted during this time). Orange crystals suitable for single crystal X-ray diffraction analysis were grown from the concentrated benzene-d_6 solution after storage at 4 °C for two days.

Method 2: To a solution of [^{EtDip}nacnacGa{(C_6H_5)NNPh}] **4a** (80.0 mg, 114 µmol, 1.0 equiv.) in toluene (10 mL) was added 2,3-dimethyl-1,3-butadiene (14.3 µL, 126 µmol, 1.1 equiv.), and the resulting orange solution was stirred at 80 °C for seven days. All volatiles were removed *in vacuo* before the residue was taken up in *n*-hexane (5 mL). The dark orange precipitate was filtered off and dried under vacuum affording compound **5** with *ca.* 3% of compound **4a** according to the integration in the ^{1}H NMR spectrum (Figure S16). Storing the solution at -40 °C overnight afforded the second crop of compound **5**. Yield: 48.5 mg (61%). Mp.: 213-214 °C. ^{1}H NMR (499.9 MHz, benzene-d_6, 298 K) δ = 0.81 (d, J_{HH} = 6.6 Hz, 6H, Ar-*o*-CH(C*H*$_3$)$_2$), 0.94 (d, J_{HH} = 6.9 Hz, 6H, Ar-*o*-CH(C*H*$_3$)$_2$), 1.03 (t, J_{HH} = 7.5 Hz, 6H, Et-C*H*$_3$), 1.18 (d, J_{HH} = 6.8 Hz, 6H, Ar-*o*-CH(C*H*$_3$)$_2$), 1.49 (d, J_{HH} = 6.9 Hz, 6H, Ar-*o*-CH(C*H*$_3$)$_2$), 1.98 (m, J_{HH} = 7.2 Hz, 2H, Et-C*H*$_2$), 2.10 (m, J_{HH} = 7.9 Hz, 2H, Et-C*H*$_2$), 3.15 (sept, J_{HH} = 6.7 Hz, 2H, Ar-*o*-C*H*(CH$_3$)$_2$), 3.53 (sept, J_{HH} = 6.8 Hz, 2H, Ar-*o*-C*H*(CH$_3$)$_2$), 5.45 (s, 1H, NC(CH(CH$_3$)$_2$)C*H*), 5.50 (s, 1H, N-*H*), 6.57 (m, 3H, Ph-*H*), 6.69 (m, 1H, Ph-*H*), 6.80 (m, 1H, Ph-*H*), 6.88 (m, 4H, Ar-*H*), 6.97 (m, 2H, Ar-*H*), 7.33 (br, 3H, Ph-*H*), 7.39 (dd, 1H, Ph-*H*). ^{13}C{^{1}H} NMR (125.7 MHz, benzene-d_6, 298 K): δ = 12.6 (Et-*C*H$_3$), 12.8 (Et-*C*H$_3$), 24.4 (ArCH(*C*H$_3$)$_2$), 24.7 (ArCH(*C*H$_3$)$_2$), 24.9 (ArCH(*C*H$_3$)$_2$), 24.9 (ArCH(*C*H$_3$)$_2$), 25.1 (ArCH(*C*H$_3$)$_2$), 25.3 (ArCH(*C*H$_3$)$_2$), 28.4 (Ar*C*H(CH$_3$)$_2$), 28.6 (Ar*C*H(CH$_3$)$_2$), 28.7 (Et-*C*H$_2$), 28.8 (Et-*C*H$_2$), 29.3 (Ar*C*H(CH$_3$)$_2$), 29.4 (Ar*C*H(CH$_3$)$_2$), 91.5 (NC*C*HCN), 94.47, 113.5 (DipAr-*C*H), 115.2 (Ph-*C*H), 124.6 (DipAr-*C*H) 124.83, 125.5 (Ph-*C*H), 126.7 (Ph-*C*H), 127.4 (Ph-*C*H), 128.0 (Ph-*C*H), 128.2 (Ph-*C*H), 128.4 (Ph-*C*H), 128.6 (Ph-*C*H), 128.8 (Ph-*C*H), 139.0 (Ph-*C*H), 139.8 (Ar-*C*), 144.3 (Ar-*C*), 144.7 (Ar-*C*), 146.1 (Ph-*C*H), 152.7 (Ph-*C*H), 176.9 (Ar-*o*-*C*), 178.0 (N*C*(CH(CH$_3$)$_2$)). IR (ATR), ν (cm^{-1}): 3262 (w, N-H), 2965 (s), 2866 (w), 1408 (s), 1063 (s, C-N), 741 (s).

Synthesis of [(^{EtDip}nacnac)Ga(PhNNHPh)($CH_2S(O)Me$)] 6

Method 1: To a J. Young NMR tube containing a solution of compound **4a** (10.0 mg, 14.3 μmol) in benzene-d_6 (0.6 mL) was added DMSO-d_6 (ca. 2.00 μL, 28.5 μmol) affording a yellow solution immediately. The reaction process was monitored by ^{1}H NMR spectroscopy, which shows the formation of a new nacnac-based species. The resulting reaction mixture was concentrated and stored at -4 °C for two days and yielded yellow crystals of **6** suitable for single crystal X-ray diffraction analysis.

Method 2: To a solution of compound **4a** (100 mg, 143 μmol, 1.0 equiv.) in toluene (10 mL) was added DMSO (distilled, 20.0 μL, 285 mmol, 2.0 equiv.) at room temperature and the resulting orange solution was stirred for one hour, during which a colour chance to bright yellow was observed. Volatiles were removed under reduced pressure, and the residue was extracted with *n*-hexane (10 mL). The pale-yellow precipitate formed was dried under vacuum and analysed by NMR spectroscopy to be compound **6**. Colourless crystals were obtained from a concentrated *n*-hexane solution. Yield: 78.2 mg (70%). Mp.: 217-218 °C. ^{1}H NMR (499.9 MHz, benzene-d_6, 298 K) δ = 0.88 (d, J_{HH} = 6.7 Hz, 3H, Ar-*o*-CH(C*H*$_3$)$_2$), 0.93 (t, 3H, Et-C*H*$_3$), 0.99 (t, 3H, Et-C*H*$_3$), 1.02 (d, J_{HH} = 6.7 Hz, 3H, Ar-*o*-CH(C*H*$_3$)$_2$), 1.04 (d, J_{HH} = 4.3 Hz, 3H, Ar-*o*-CH(C*H*$_3$)$_2$), 1.05 (d, J_{HH} = 5.1 Hz, 3H, Ar-*o*-CH(C*H*$_3$)$_2$), 1.11 (d, J_{HH} = 3.9 Hz, 3H, Ar-*o*-CH(C*H*$_3$)$_2$), 1.12 (d, J_{HH} = 3.8 Hz, 3H, Ar-*o*-CH(C*H*$_3$)$_2$), 1.26 (d, J_{HH} = 6.8 Hz, 3H, Ar-*o*-CH(C*H*$_3$)$_2$), 1.46 (d, J_{HH} = 6.6 Hz, 3H, Ar-*o*-CH(C*H*$_3$)$_2$), 1.52 (d, J_{HH} = 12.1 Hz, 1H, SC*H*$_2$), 1.63 (s, 3H, SC*H*$_3$), 1.90 (m, J_{HH} = 5.0 Hz, 2H, Et-C*H*$_2$), 1.99 (d, J_{HH} ≈ 10 Hz, 1H, SC*H*$_2$), 2.08 (m, J_{HH} = 5.3 Hz, 2H, Et-C*H*$_2$), 3.12 (sept, J_{HH} = 5.0 Hz, 1H, Ar-*o*-C*H*(CH$_3$)$_2$), 3.19 (sept, J_{HH} = 5.0 Hz, 2H, Ar-*o*-C*H*(CH$_3$)$_2$), 3.41 (sept, J_{HH} = 6.5 Hz, 1H, Ar-*o*-C*H*(CH$_3$)$_2$), 5.40 (s, 1H, NCC*H*CN), 6.66 (t, J_{HH} = 7.2 Hz, 1H, Ph-*H*), 6.74–6.80 (m, 2H, Ph-*H*), 6.82 (d, J_{HH} = 1.3 Hz, 1H, Ph-*H*), 6.97 (m, 3H, Ar-*H*), 7.01 (m, 2H, Ph-*H*), 7.07 (m, J_{HH} = 7.4, 1H, Ar-*H*), 7.13 (t, J_{HH} = 8.7 Hz, 1H, Ar-*H*), 7.22 (m, 2H, Ar-*H*), 7.27 (m, 2H, Ar-*H*), 8.90 (s, 1H, N*H*). ^{13}C{^{1}H} NMR (125.7 MHz, benzene-d_6, 298 K): δ = 12.6 (Et-*C*H$_3$), 13.0 (Et-*C*H$_3$), 23.7 (Ar-*o*-CH(*C*H$_3$)$_2$), 23.8 (Ar-*o*-CH(*C*H$_3$)$_2$), 23.9 (Ar-*o*-CH(*C*H$_3$)$_2$), 24.1 (Ar-*o*-CH(*C*H$_3$)$_2$), 25.0 (Ar-*o*-CH(*C*H$_3$)$_2$), 25.1 (Ar-*o*-CH(*C*H$_3$)$_2$), 25.4 (Ar-*o*-CH(*C*H$_3$)$_2$), 25.5 (Ar-*o*-CH(*C*H$_3$)$_2$), 27.6 (Ar-*o*-*C*H(CH$_3$)$_2$),175.9 (N*C*(CH$_2$CH$_3$)), 27.9 (Ar-*o*-*C*H(CH$_3$)$_2$), 28.1 (Ar-*o*-*C*H(CH$_3$)$_2$), 28.2 (Ar-*o*-*C*H(CH$_3$)$_2$), 28.5 (Et-*C*H$_2$), 28.7 (Et-*C*H$_2$), 38.5 (S*C*H$_2$), 43.7 (S*C*H$_3$), 92.3 (NC*C*HCN), 113.4 (NH(Ph-*o*-*C*H)), 113.9 (NH(Ph-*C*)), 115.94 (NH(Ph-*p*-*C*H)), 116.9 (NH(Ph-*m*-*C*H)), 123.4 (Ar-*m*-*C*H), 123.9 (Ar-*m*-*C*H), 125.4 (Ar-*p*-*C*H), 127.6 ((Ga-N(Ph-*C*H)), 127.8 ((Ga-N(Ph-*C*H)), 128.4 ((Ga-N(Ph-*C*H)), 140.6 (Ar-*C*-CH(CH$_3$)$_2$), 142.7 (Ar-*o*-*C*-CH(CH$_3$)$_2$), 145.9 (Ar-*o*-*C*-CH(CH$_3$)$_2$), 147.1 (Ar-*o*-*C*-CH(CH$_3$)$_2$), 150.9 ((Ga-N(Ph-*C*)), 174.5 (N*C*(Et)).

Synthesis of [(^{EtDip}nacnac)Ga(PhNN(Ph)CH(Ph)O)] 7

To a solution of [^{EtDip}nacnacGa{(C_6H_5)NNPh}] **4a** (80.0 mg, 114 µmol, 1.0 equiv.) in toluene (20 mL) was added distilled benzaldehyde PhCHO (12.8 µL, 126 µmol, 1.1 equiv.) slowly and stirred at 50 °C for three hours until a bright yellow solution was observed. Volatiles were then removed under reduced pressure, and the residue was extracted with *n*-hexane (10 mL). Storing the solution at -40 °C for one day afforded **7** as star-shaped yellow crystalline solid, which was recrystallised from *n*-pentane affording large yellow crystals of **7** suitable for single crystal X-ray diffraction analysis. Yield: 50.8 mg (55%). Mp.: 240-241 °C. ^{1}H NMR (499.9 MHz, benzene-d_6, 343 K) δ = 0.93 (2t, J_{HH} = 9.9, 7.5 Hz, 6H, Et-CH_3), 1.06 (2d, J_{HH} = 6.5, 4.2 Hz, 6H, Ar-*o*-CH(CH_3)$_2$), 1.11 (2d, J_{HH} = 6.8, 2.7 Hz, 6H, Ar-*o*-CH(CH_3)$_2$), 1.20 (d, J_{HH} = 6.9 Hz, 3H, Ar-*o*-CH(CH_3)$_2$), 1.38 (d, J_{HH} = 6.7 Hz, 3H, Ar-*o*-CH(CH_3)$_2$), 1.68 (d, J_{HH} = 6.7 Hz, 3H, Ar-*o*-CH(CH_3)$_2$), 2.07 (m, 2H, Et-CH_2) 2.17 (m, 2H, Et-CH_2), 3.08 (br s, 1H, ArC*H*(CH$_3$)$_2$), 3.24 (sept, 1H, ArC*H*(CH$_3$)$_2$), 3.37 (sept, 1H, ArC*H*(CH$_3$)$_2$), 3.48 (br s, 1H, ArC*H*(CH$_3$)$_2$), 5.31 (s, 1H, NCC*H*CN), 5.62 (br, 1H, OC*H*(Ph)N), 6.57 – 6.69 (m, 6H, Ph-*H*), 6.88-6.95 (m, 5H, Ph-*H*), 7.02-7.08 (m, 2H, Ar-*H*), 7.08-7.14 (m, 4H, Ar-*H*), 7.17-7.28 (m, 4H, Ph-*H*). ^{13}C{^{1}H} NMR (125.7 MHz, benzene-d_6, 343 K): δ = 12.6 (NC(CH$_2$*C*H$_3$)), 12.8 (NC(CH$_2$*C*H$_3$)), 24.4 (ArCH(*C*H$_3$)$_2$), 24.7 (ArCH(*C*H$_3$)$_2$), 24.9 (ArCH(*C*H$_3$)$_2$), 24.9 (ArCH(*C*H$_3$)$_2$), 25.1 (ArCH(*C*H$_3$)$_2$), 25.3 (ArCH(*C*H$_3$)$_2$), 28.4 (Ar*C*H(CH$_3$)$_2$), 28.6 (Ar*C*H(CH$_3$)$_2$), 28.7 (NC(*C*H$_2$CH$_3$)), 28.8 (NC(*C*H$_2$CH$_3$)), 29.3 (Ar*C*H(CH$_3$)$_2$), 29.4 (Ar*C*H(CH$_3$)$_2$), 91.5 (NC*C*HCN), 94.5, 113.5 (DipAr-*C*H), 115.2 (Ph-*C*H), 124.6 (DipAr-*C*H), 124.83, 125.5 (Ph-*C*H), 126.7 (Ph-*C*H), 127.4 (Ph-*C*H), 128.0 (Ph-*C*H), 128.2 (Ph-*C*H), 128.4 (Ph-*C*H), 128.6 (Ph-*C*H), 128.8 (Ph-*C*H), 139.0 (Ph-*C*H), 139.8 (Ar-*C*), 144.3 (Ar-*C*), 144.7 (Ar-*C*), 146.1 (Ph-*C*H), 152.7 (Ph-*C*H), 176.9 (Ar-*o*-*C*), 178.0 (N*C*(CH(CH$_3$)$_2$)). IR (ATR), v (cm^{-1}): 2963, 2870 (s, C-H), 1593 (s), 1526 (s), 1410 (s), 1240 (s, C-O), 1059 (s, C-N), 741 (s). Elemental analysis: calculated for $C_{50}H_{61}GaN_4O$: C 74.71; H 7.65; N 6.97%; found: C 74.67; H 7.71; N 6.57%.

[^{EtDip}nacnacGa(H)-Al(H_2)(NHC)] 8

To a J. Young NMR tube charged with benzene-d_6 solutions (0.6 mL) of **2a** (30.0 mg, 0.058 mol, 1.0 equiv.) was added a stoichiometric amount of (NHC)AlH_3 (12.3 mg, 0.058 mol, 1.0 equiv.). The predominant consumption of compound **2a** and the formation of a new compound (90% estimated *in-situ* yield of **8**) were observed after keeping the reaction mixture at room temperature for two days. Yellow crystals suitable for single crystal X-ray diffraction analysis were grown from a concentrated mixed benzene-d_6 and *n*-hexane solution at 4 °C over two days. ^{1}H NMR (400.1 MHz, benzene-d_6, 294 K) δ = 1.02 (t, 6H, Et-CH_3), 1.03 (d, J_{HH} = 6.6 Hz, 6H, carbene-*i*Pr-CH_3), 1.12 (d, J_{HH} = 6.2 Hz, 6H, carbene-*i*Pr-CH_3), 1.15 (d, J_{HH} = 6.6 Hz, 6H, Ar-*o*-CH(CH_3)$_2$), 1.29 (d, J_{HH} = 6.9 Hz, 6H, Ar-*o*-CH(CH_3)$_2$), 1.32 (d, J_{HH} = 6.9 Hz, 6H, Ar-*o*-CH(CH_3)$_2$), 1.50 (s, 6H, carbene-(CH_3)$_2$C$_2$), 1.73 (d, J_{HH}

= 6.8 Hz, 6H, Ar-*o*-CH(C*H*$_3$)$_2$), 2.19–1.94 (m, J_{HH} = 7.6 Hz, 6H, Et-C*H*$_2$), 3.63 (sept, J_{HH} = 6.8 Hz, 2H, Ar-*o*-C*H*(CH$_3$)$_2$), 3.87 (sept, J_{HH} = 7.1 Hz, 2H, Ar-*o*-C*H*(CH$_3$)$_2$), 5.07 (sept, J_{HH} = 7.1 Hz, 2H, carbene-*i*Pr-C*H*), 5.16 (s, 1H, NCC*H*CN), 6.20 (s, br, 1H, Ga-*H*), 7.18-7.12 (m, 4H, Ar-*m*-C*H*), 7.25 (dd, J_{HH} = 6.6, 2.6 Hz, 2H, Ar-*p*-C*H*). ^{13}C NMR (125.7 MHz, benzene-*d*$_6$, 298 K) δ 9.4 ((*C*H$_3$)C=C(*C*H$_3$)), 9.6 (Ar-*o*-CH(*C*H$_3$)$_2$), 12.5 (carbene-*i*Pr-*C*H$_3$), 21.2 (Ar-*o*-CH(*C*H$_3$)$_2$), 21.4 (Et-*C*H$_3$), 23.5 (Ar-*o*-CH(*C*H$_3$)$_2$), 24.5 (Ar-*o*-CH(*C*H$_3$)$_2$), 24.7 (Ar-*o*-CH(*C*H$_3$)$_2$), 26.5 (Ar-*o*-CH(*C*H$_3$)$_2$), 27.4 (Ar-*o*-*C*H(CH$_3$)$_2$), 27.9 (Et-*C*H$_2$), 28.8 (Ar-*o*-*C*H(CH$_3$)$_2$), 51.9 (carbene-*i*Pr-*C*H), 52.1 (Ar-*o*-*C*H(CH$_3$)$_2$), 90.0 (NC*C*HCN), 123.2 (Ar-*m*-*C*H), 125.2 ((CH$_3$)*C*=*C*(CH$_3$)), 125.4 (Ar-*p*-*C*H), 143.2 (Ar-*o*-*C*-CH(CH$_3$)$_2$), 144.5 (Ar-*ipso*-*C*), 145.1 (Ar-*o*-*C*), 171.5 (N*C*(Et)). IR (ATR), ν (cm^{-1}): 2963 (s), 2862 (w), 1753 (s, Ga-H), 1709 (vs, br, likely contains both Al-H units), 1551 (s), 1522 (s), 1406 (s), 1260 (s), 1072 (s), 793 (s). Note, the elemental analysis provided C,H,N values approximately 0.5% (H) to 3% (C) too low.

[(^{EtDip}nacnac)GaH$_2$] 9

A 0.49 M solution of AlH$_3$(NMe$_3$) (1.05 mL, 513 µmol, 1.1 equiv.) in toluene was added to a solution of [(^{EtDip}nacnac)Ga] **2a** (240 mg, 466 µmol, 1.0 equiv.) in toluene (20 mL) at 0 °C. The resulting yellowish solution was allowed to stir at room temperature for 30 minutes before it was heated to 80 °C and stirred for another fifteen minutes, during which the formation of a metal precipitation was observed. All the volatiles were removed *in vacuo* and the residue was extracted with *n*-hexane (10 mL). The hexane solution was stored at -40 °C for three days afforded compound **9** as large colourless block crystals, which were suitable for single crystal X-ray diffraction analysis. Yield (crystals): 45.5 mg (19%). Mp.: 216-217 °C (melted). ^{1}H NMR (499.9 MHz, benzene-*d*$_6$, 298 K) δ = 0.98 (t, J_{HH} = 7.5 Hz, 6H, Et-C*H*$_3$), 1.16 (d, J_{HH} = 6.9 Hz, 12H, Ar-*o*-CH(C*H*$_3$)$_2$), 1.38 (d, J_{HH} = 6.9 Hz, 12H, Ar-*o*-CH(C*H*$_3$)$_2$), 2.00 (q, J_{HH} = 7.5 Hz, 4H, Et-C*H*$_2$), 3.43 (sept, J_{HH} = 6.9 Hz, 4H, Ar-*o*-C*H*(CH$_3$)$_2$), 4.98 (s, 1H, NC(Et)C*H*), 5.20 (s, br, 2H, Ga*H*$_2$), 7.12 (m, J_{HH} = 5.4 Hz, 6H, Ar-*H*). ^{13}C{^{1}H} NMR (125.7 MHz, benzene-*d*$_6$, 298 K): δ = 12.3 (Et-*C*H$_3$), 24.1 (Ar-*o*-CH(*C*H$_3$)$_2$), 25.4 (Ar-*o*-CH(*C*H$_3$)$_2$), 27.6 (Et-*C*H$_2$), 27.9 (Ar-*o*-*C*H(CH$_3$)$_2$), 88.8 (NC(Et)*C*H), 124.4 (Ar-*CH*), 126.7 (Ar-*CH*), 140.4 (Ar-*ipso*-*C*), 144.0 (Ar-*o*-*C*-*i*Pr), 172.7 (N*C*(Et)). IR (ATR), ν (cm^{-1}): 2965 (s), 2926 (s), 2864 (w), 1892 (s, Ga-H), 1863 (s, Ga-H), 1530 (s), 1408 (s), 1312 (s), 1072 (s, C-N), 758 (s). Elemental analysis: calculated for $C_{31}H_{45}GaN_2$: C 71.96; H 9.16; N 5.41%; found: C 71.92; H 9.26; N 5.40%.

2 NMR spectroscopy

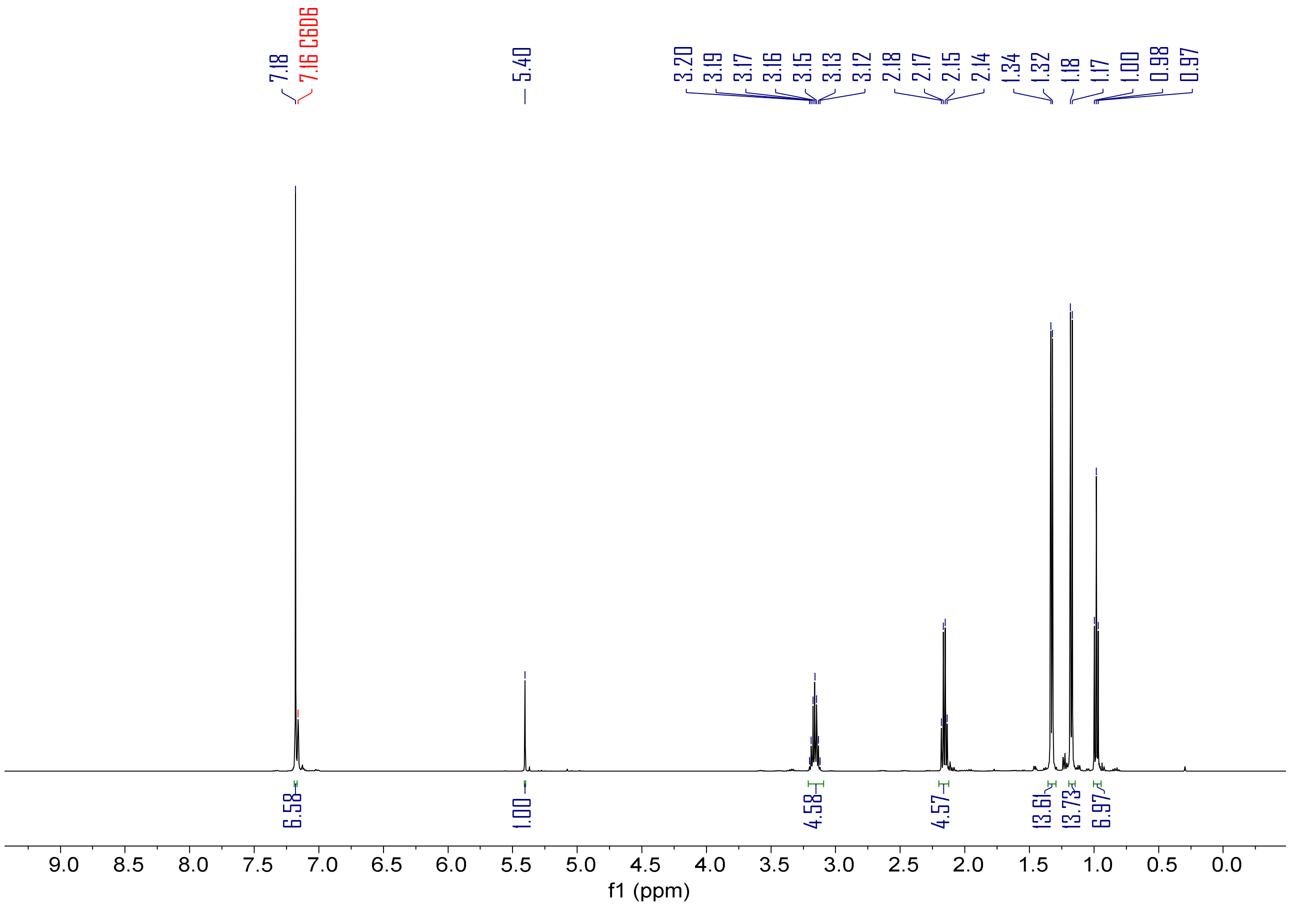

Figure S1. ^{1}H NMR spectrum (499.9 MHz, C_6D_6, 298 K) of [(^{EtDip}nacnac)Ga] **2a**.

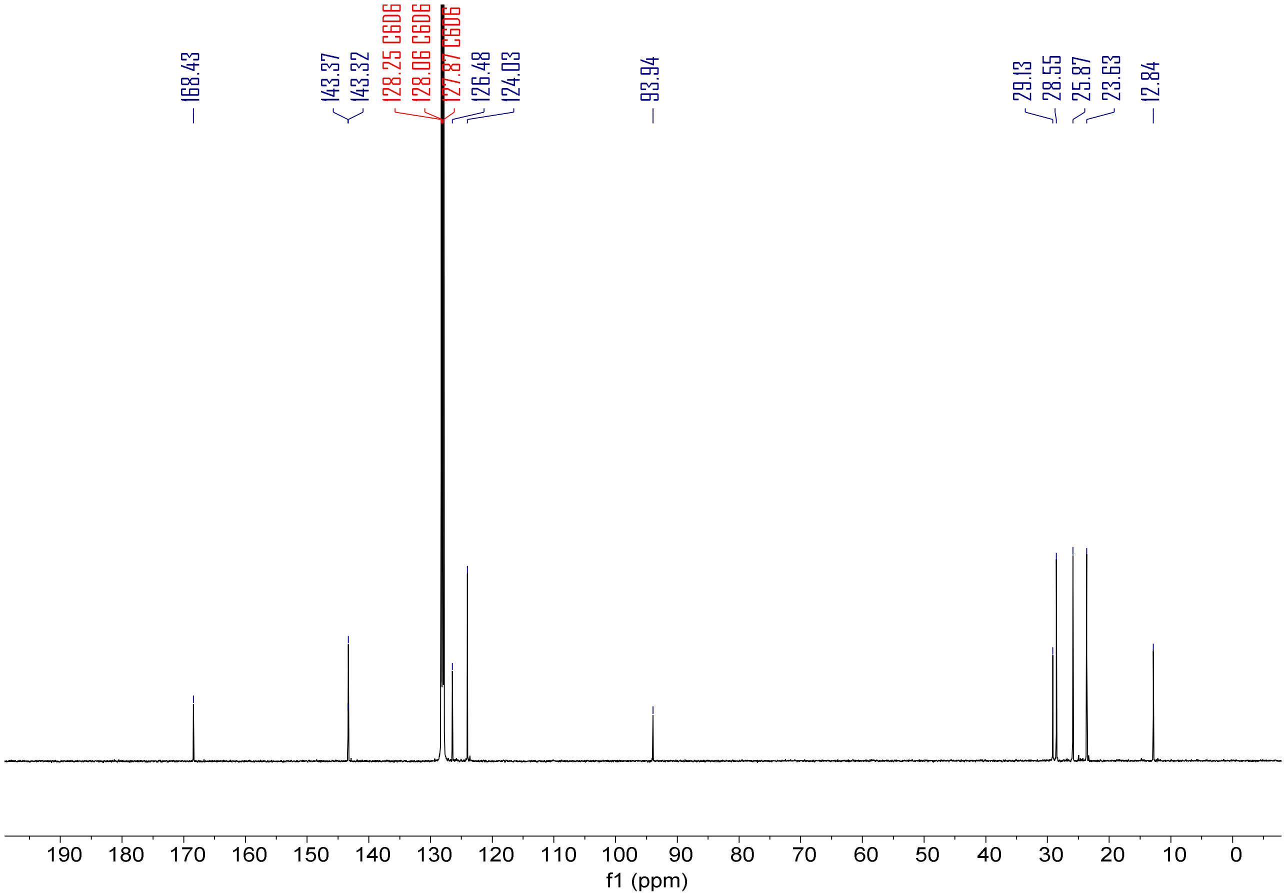

Figure S2. $^{13}C\{^1H\}$ NMR spectrum (125.7 MHz, C_6D_6, 298 K) of [(^{EtDip}nacnac)Ga] **2a**.

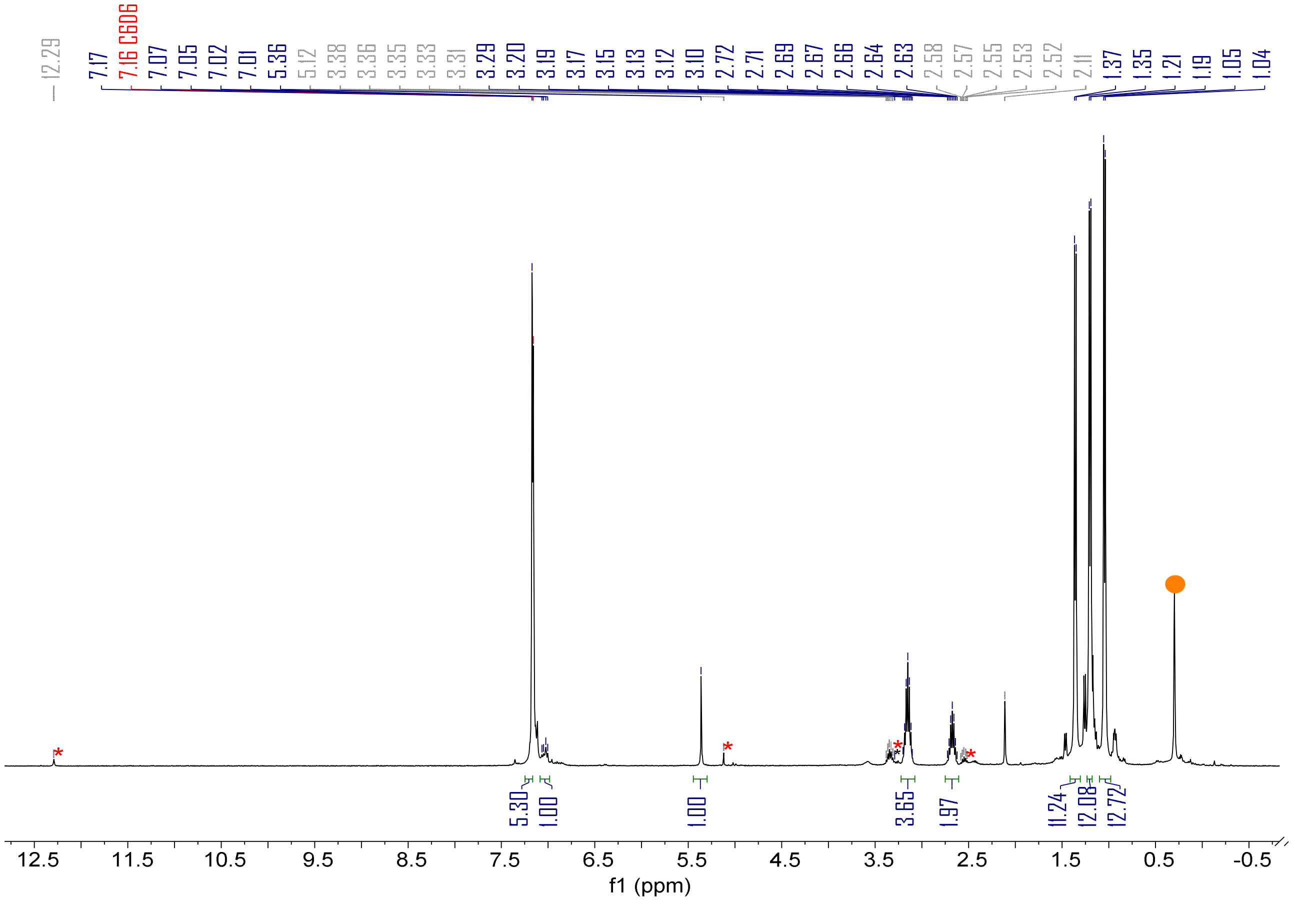

Figure S3. ^{1}H NMR spectrum (400.3 MHz, C_6D_6, 296 K) of [(^{iPrDip}nacnac)Ga] **2b**. Red asterisks denote chemical shifts of ^{iPrDip}nacnacH. The orange circle denotes the chemical resonance of silicone grease.

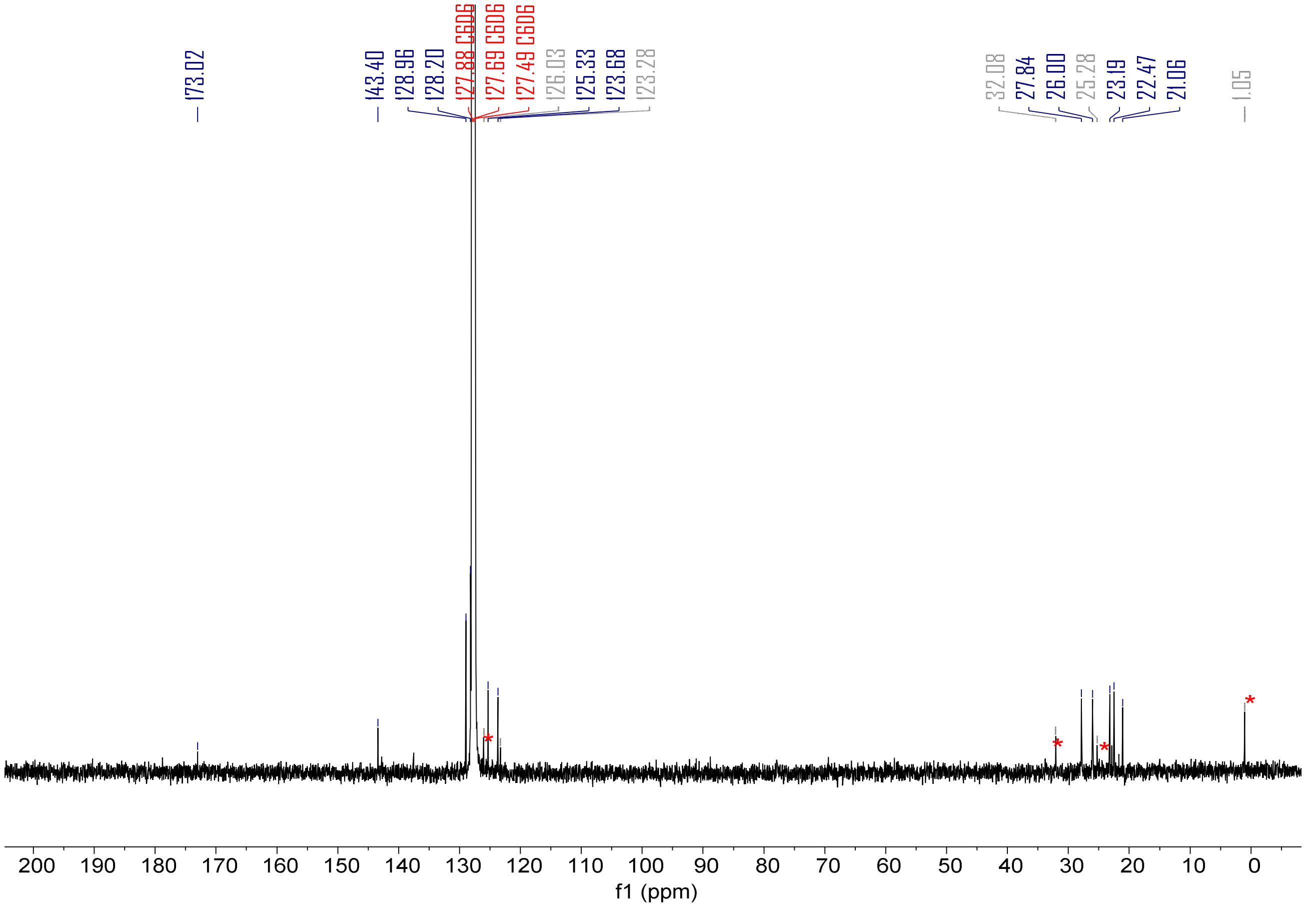

Figure S4. $^{13}C\{^{1}H\}$ NMR spectrum (125.7 MHz, C_6D_6, 298 K) of [(^{iPrDip}nacnac)Ga] **2b**. Red asterisks denote resonances of ^{iPrDip}nacnacH.

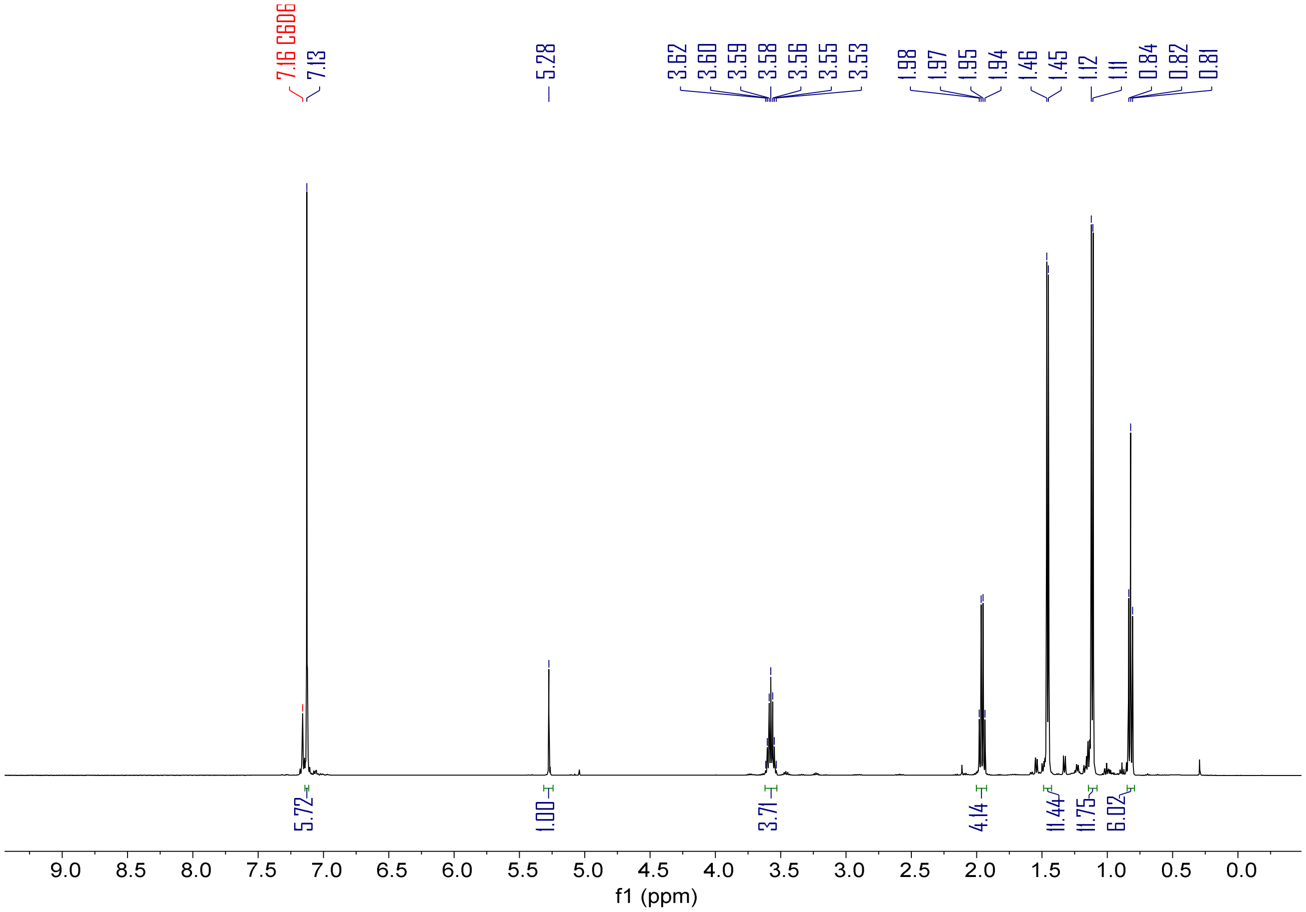

Figure S5. ^{1}H NMR spectrum (499.9 MHz, C_6D_6, 298 K) of [(^{EtDip}nacnac)GaI_2] **3a**.

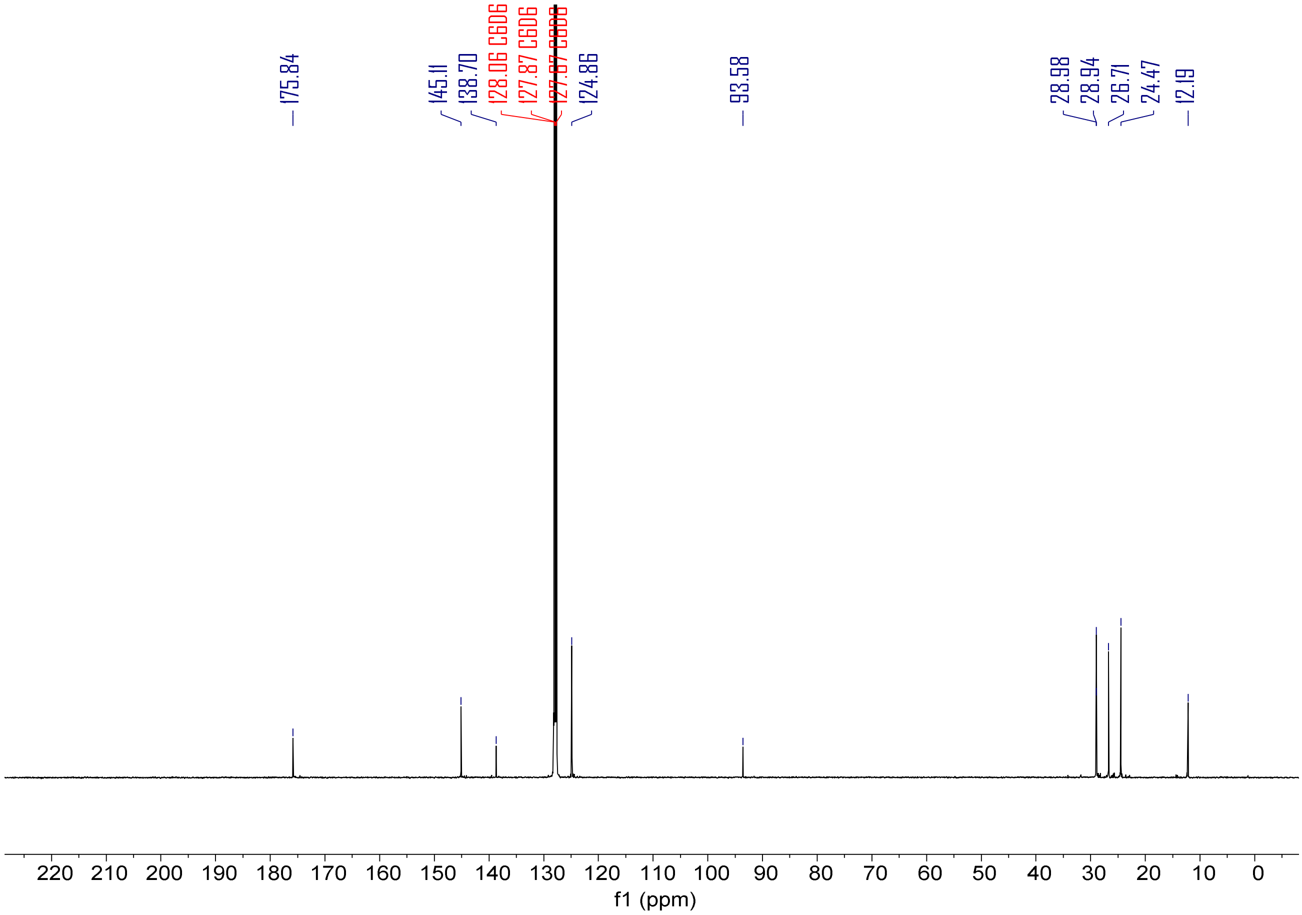

Figure S6. $^{13}C\{^{1}H\}$ NMR spectrum (125.7 MHz, C_6D_6, 298 K) of [(^{EtDip}nacnac)GaI_2] **3a**.

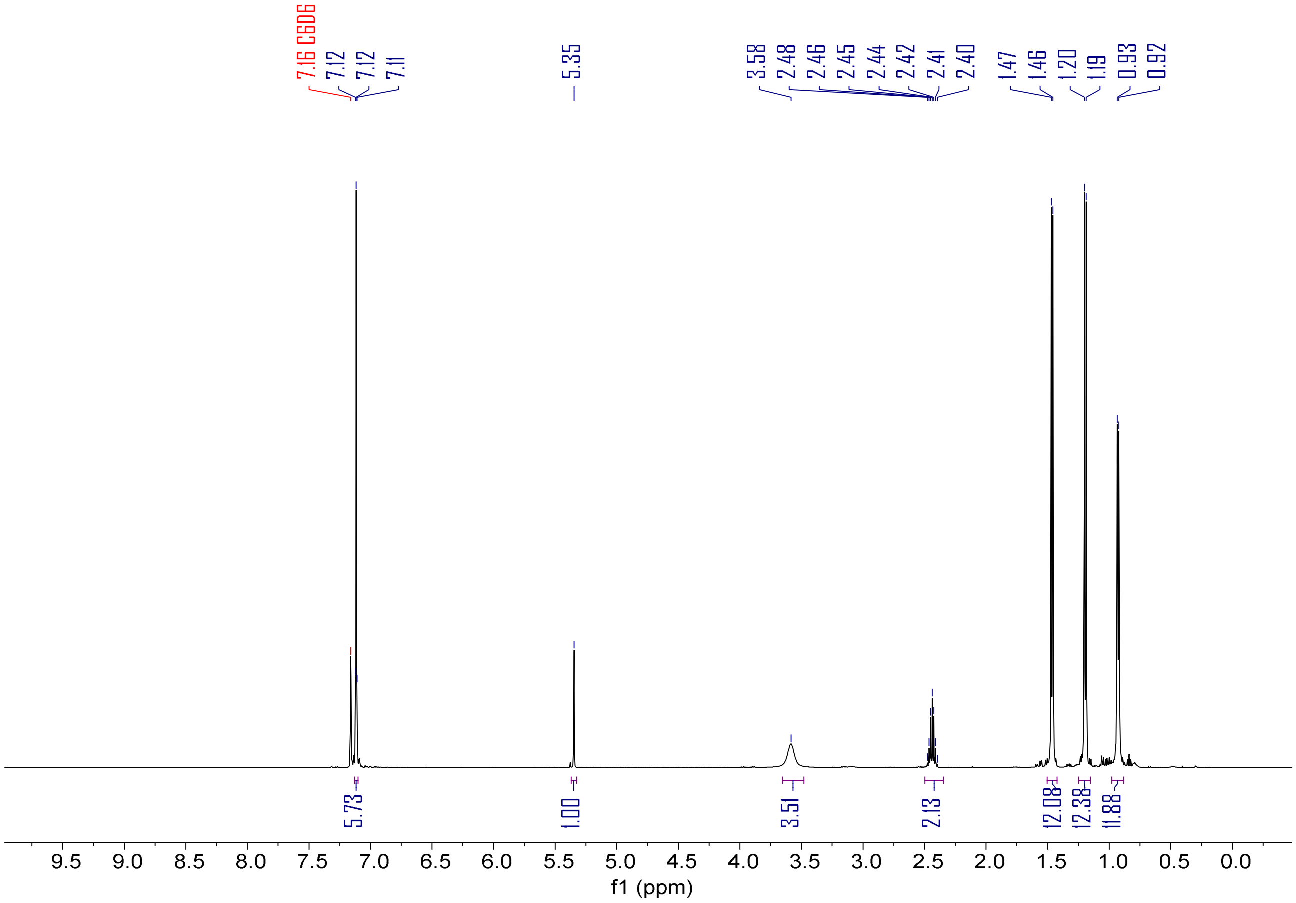

Figure S7. ^{1}H NMR spectrum (499.9 MHz, C_6D_6, 298 K) of [(^{iPrDip}nacnac)GaI_2] **3b**.

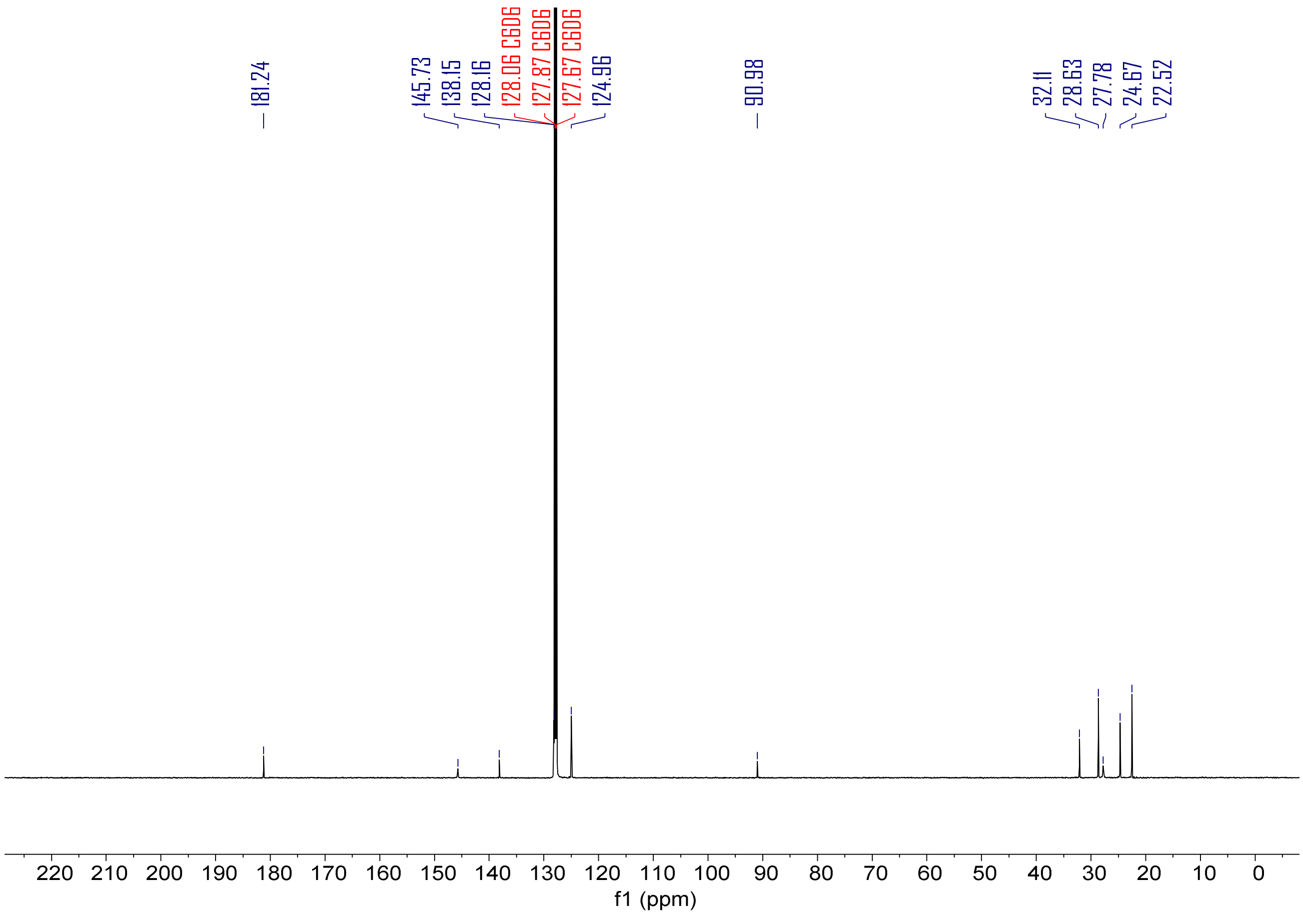

Figure S8. $^{13}C\{^{1}H\}$ NMR spectrum (125.7 MHz, C_6D_6, 295 K) of [(^{iPrDip}nacnac)GaI_2] **3b**.

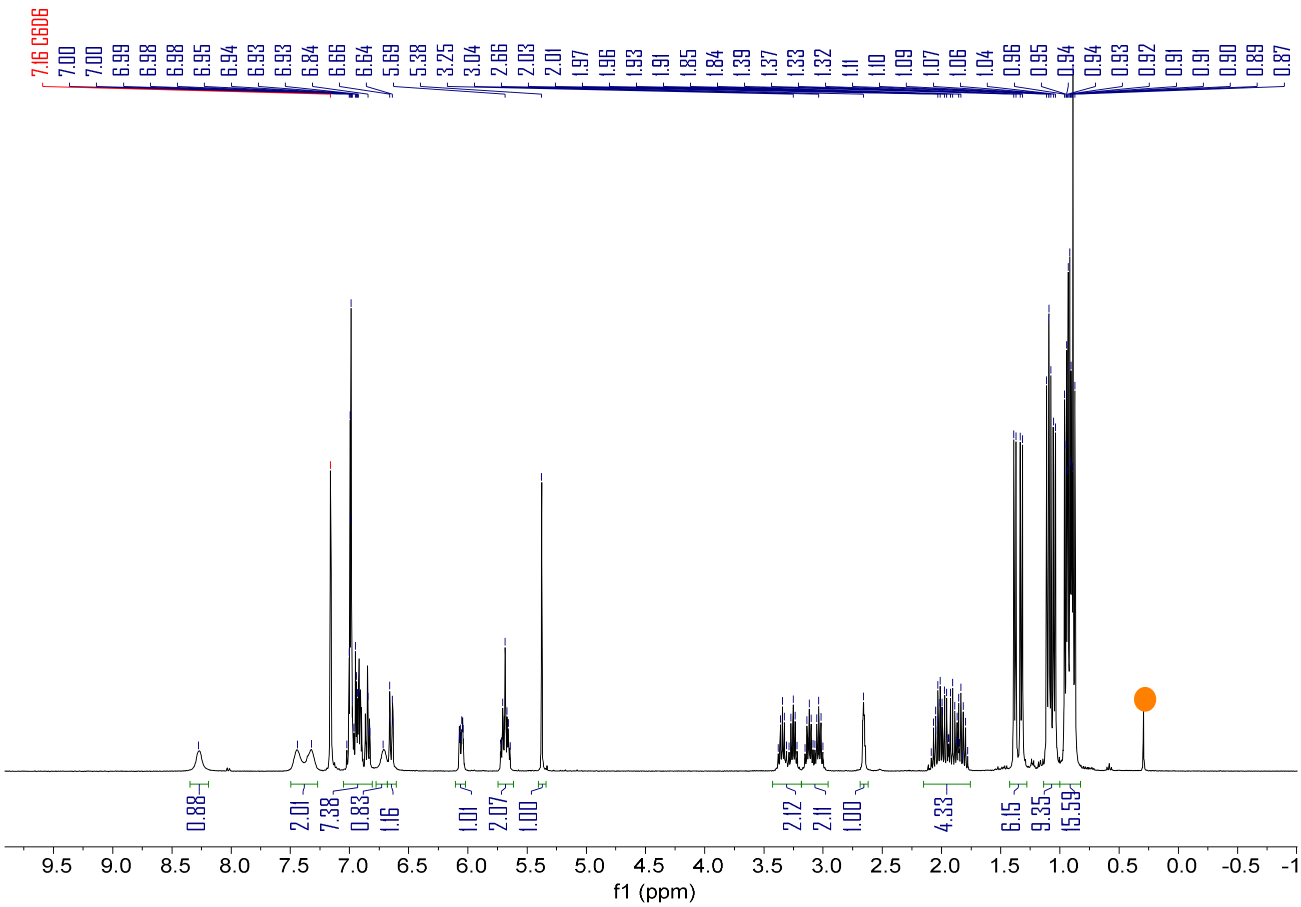

Figure S9. ^{1}H NMR spectrum (400.3 MHz, C_6D_6, 298 K) of [([EtDip]nacnac)Ga{(C_6H_5)NNPh}] **4a** from the reaction of [([EtDip]nacnac)Ga] **2a** with azobenzene. The orange circle denotes the chemical resonance of silicone grease.

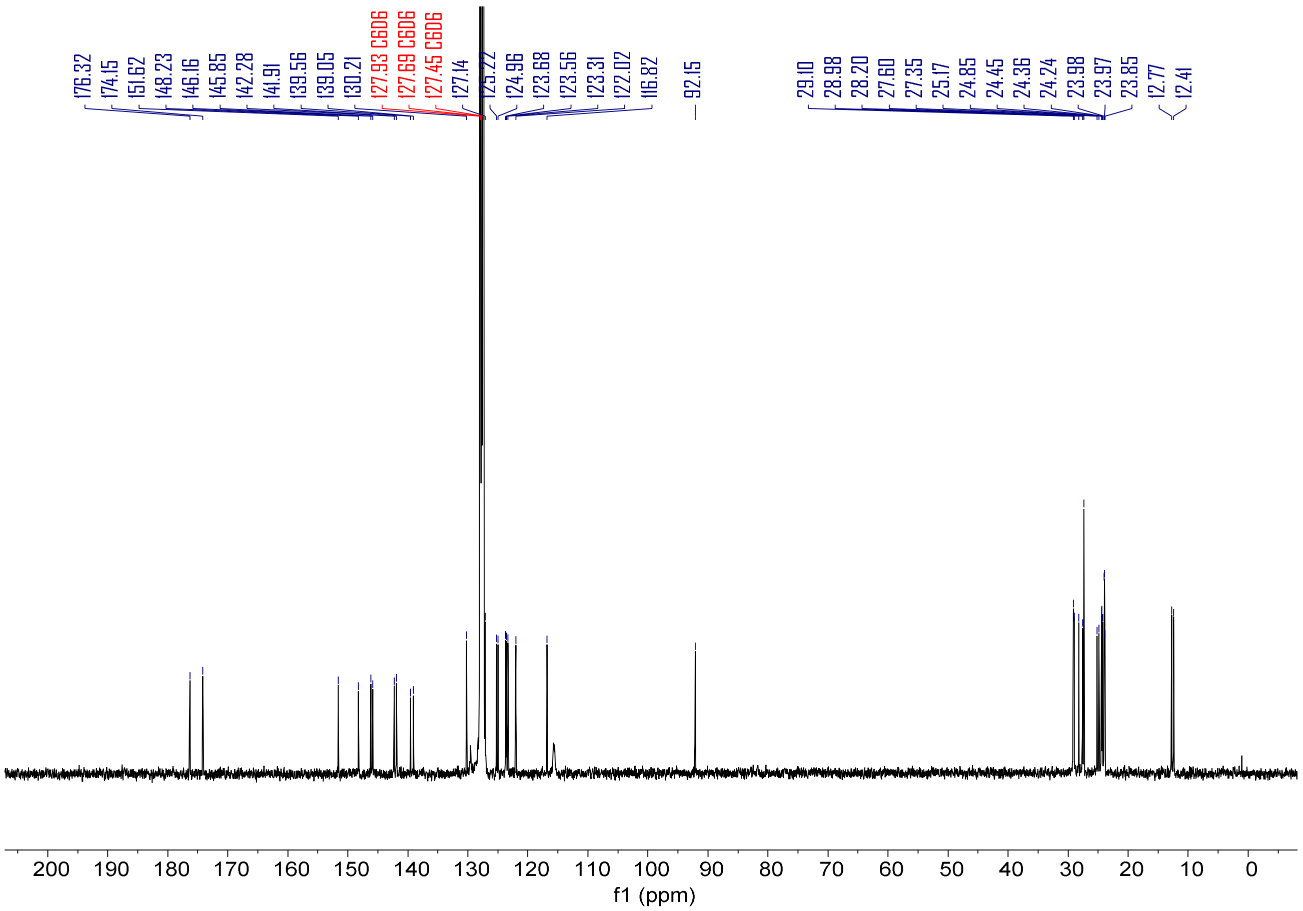

Figure S10. $^{13}C\{^{1}H\}$ NMR spectrum (100.7 MHz, C_6D_6, 298 K) of [(^{EtDip}nacnac)Ga{(C_6H_5)NNPh}] **4a**.

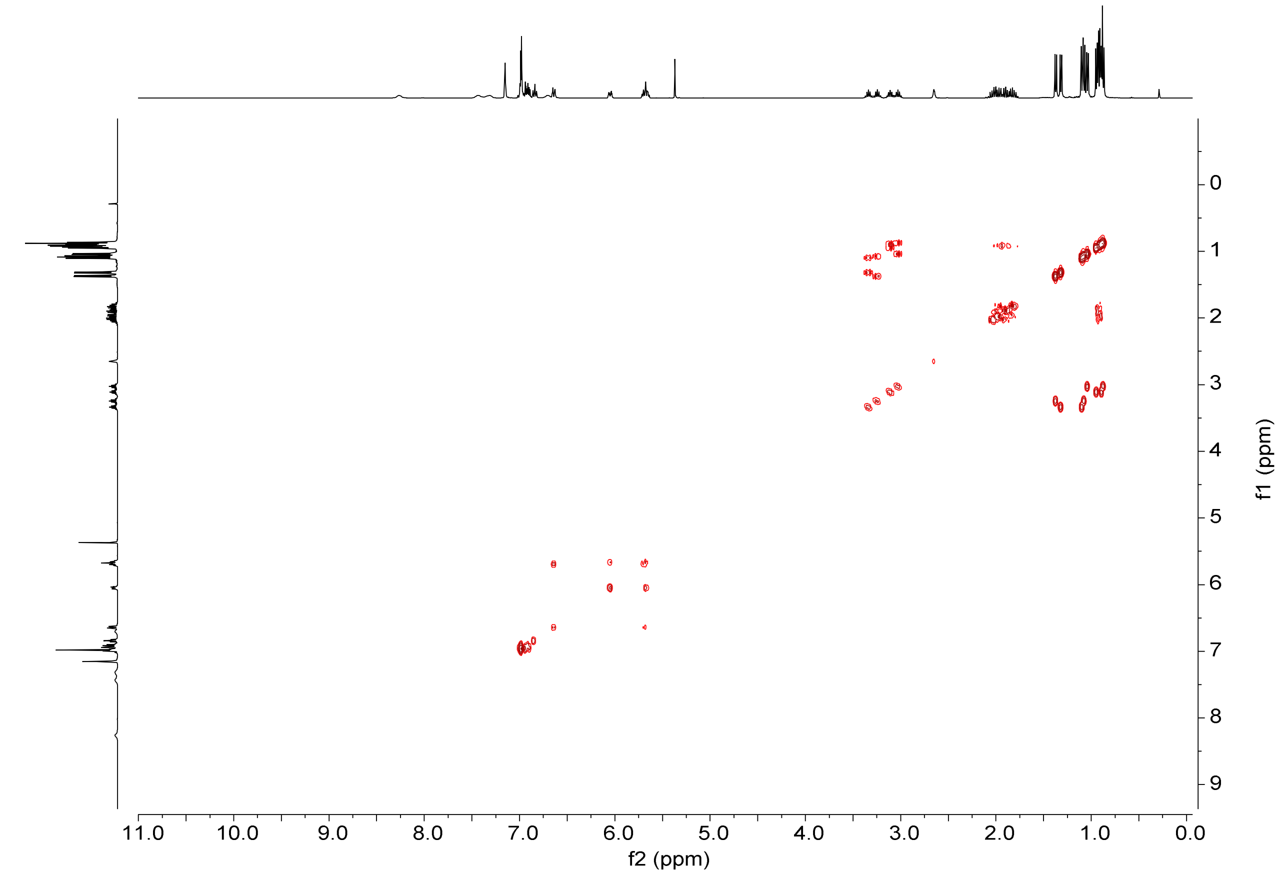

Figure S11. ^{1}H-^{1}H COSY NMR spectrum of [(^{EtDip}nacnac)Ga{(C_6H_5)NNPh}] **4a**.

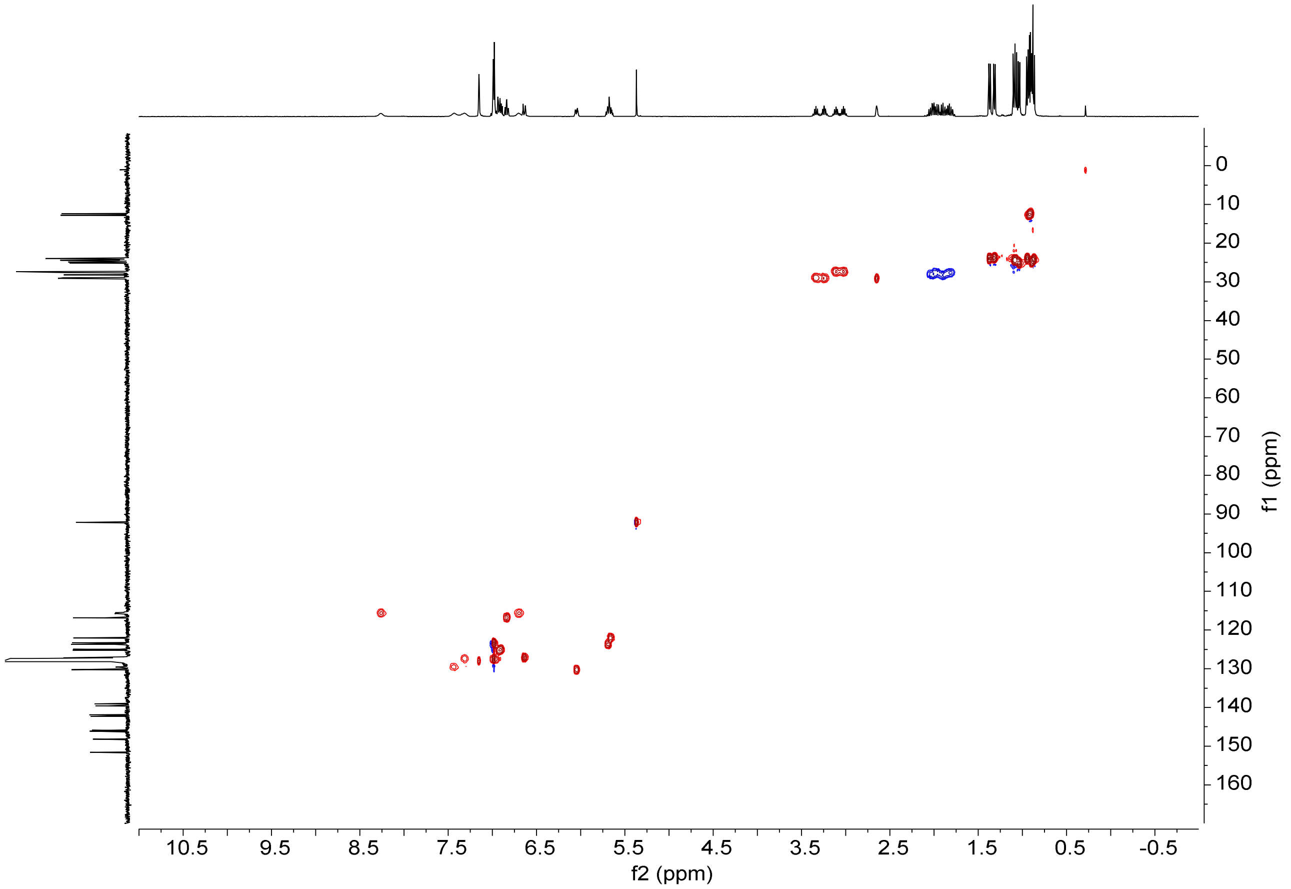

Figure S12. ^{1}H-^{13}C HSQC NMR spectrum of [(^{EtDip}nacnac)Ga{(C_6H_5)NNPh}] **4a**.

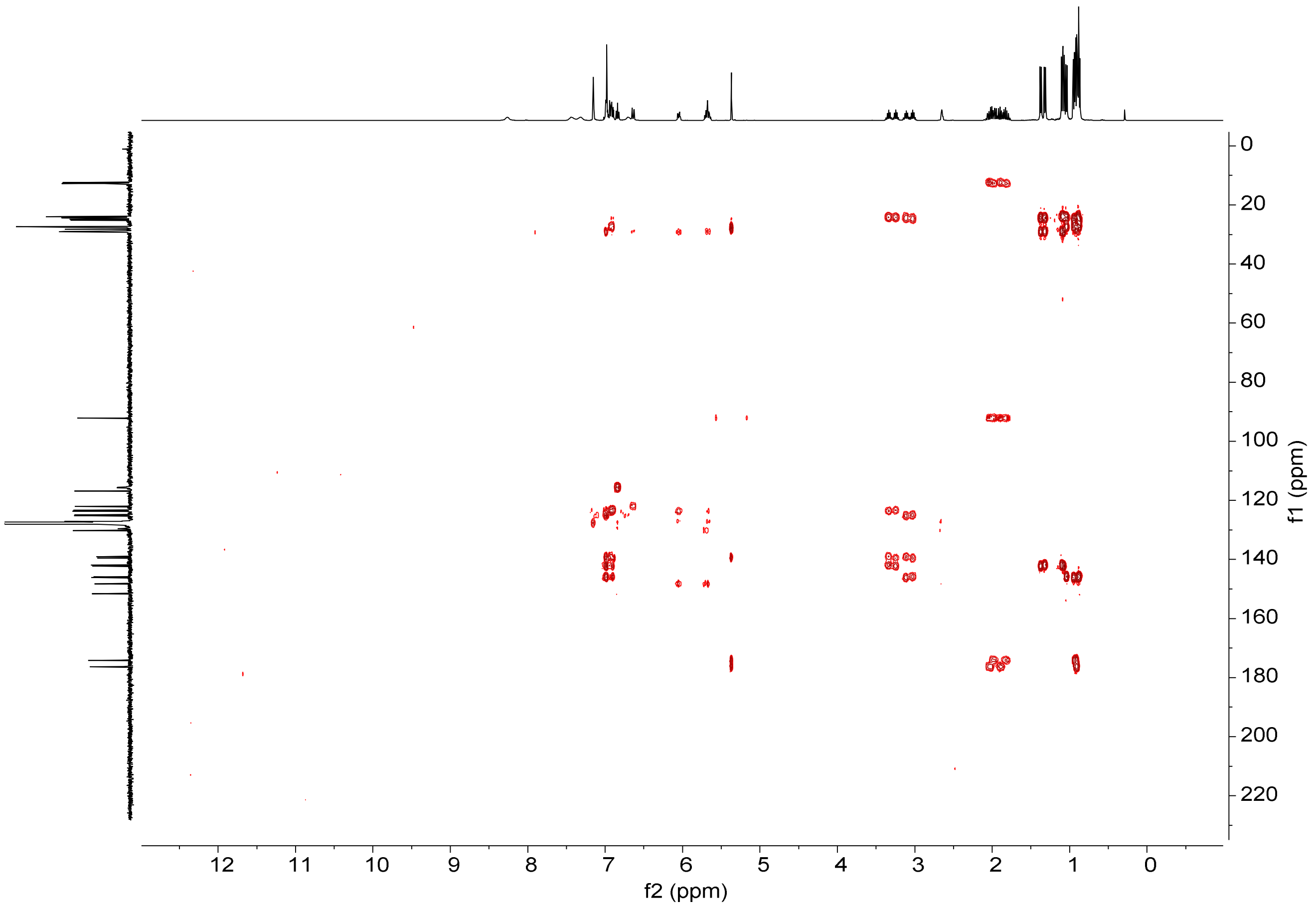

Figure S13. ^{1}H-^{13}C HMBC NMR spectrum of [(^{EtDip}nacnac)Ga{(C_6H_5)NNPh}] **4a**.

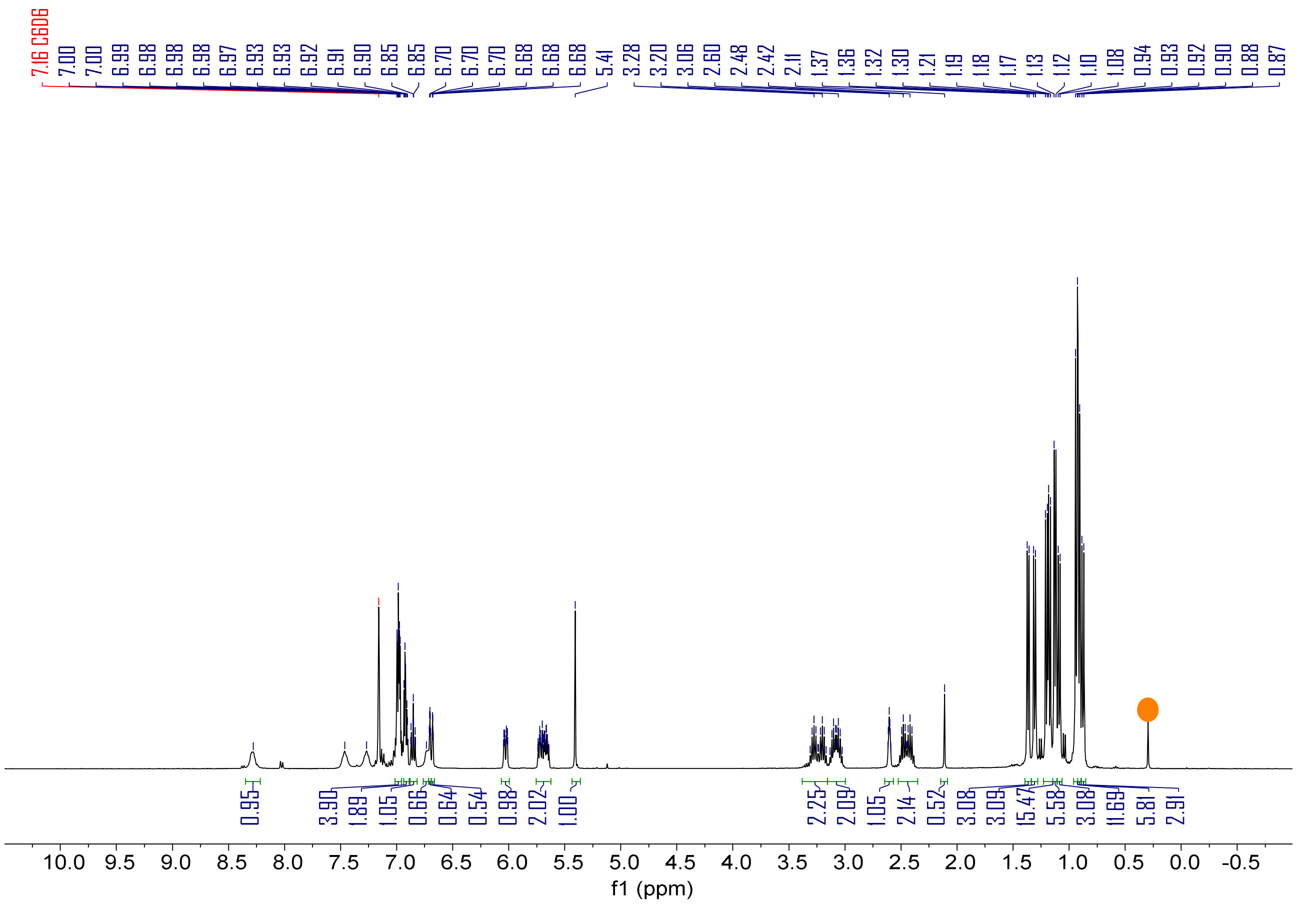

Figure S14. ^{1}H NMR spectrum (400.1 MHz, C_6D_6, 295 K) of *in-situ*-generated [(^{iPrDip}nacnac)Ga{(C_6H_5)NNPh}] **4b** from the reaction of (^{iPrDip}nacnac)Ga **2b** with azobenzene. The orange circle denotes the chemical resonance of silicone grease.

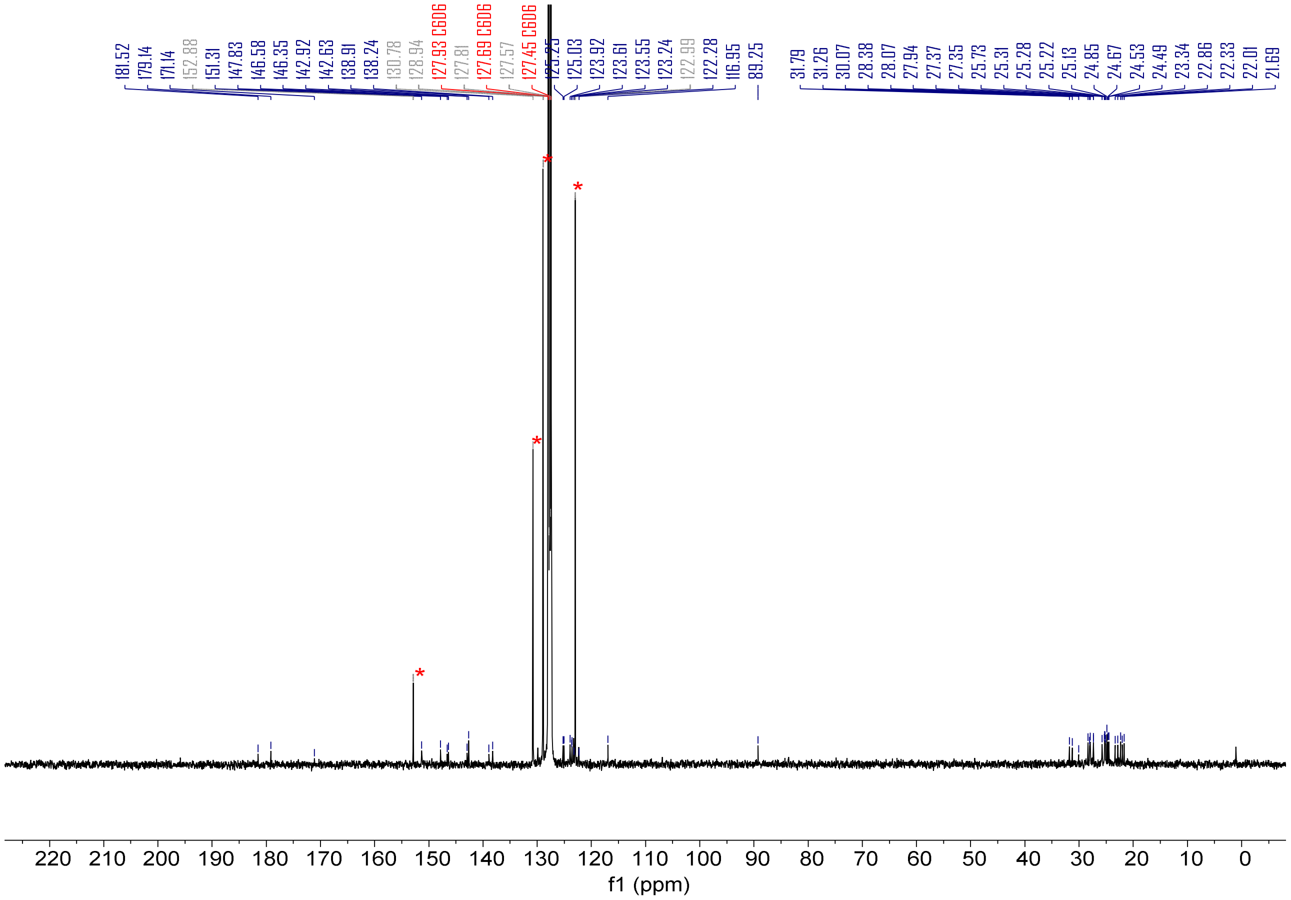

Figure S15. $^{13}C\{^{1}H\}$ NMR spectrum (100.7 MHz, C_6D_6, 298 K) of *in-situ*-generated [(^{iPrDip}nacnac)Ga{(C_6H_5)NNPh}] **4b**. The red asterisks denote resonances of excess azobenzene; the latter is difficult to mark in the ^{1}H NMR spectrum due to overlapping resonances.

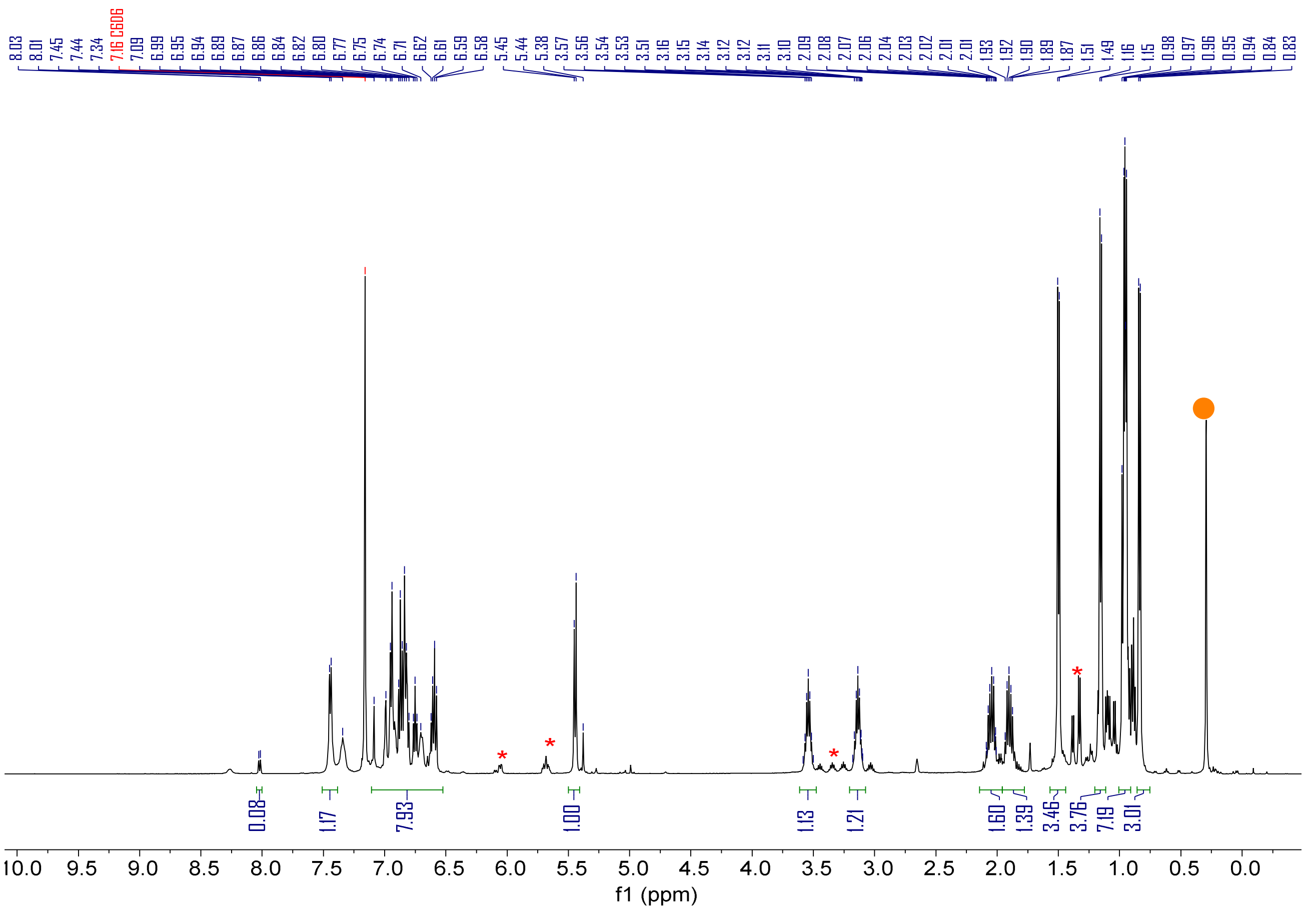

Figure S16. ^{1}H NMR spectrum (499.9 MHz, C_6D_6, 298 K) of [(^{EtDip}nacnac)Ga{(C_6H_4)N(H)NPh}] **5**. The red asterisks denote chemical shifts of residual **4a** (after precipitation via *Method 2*). The orange circle denotes the chemical resonance of silicone grease.

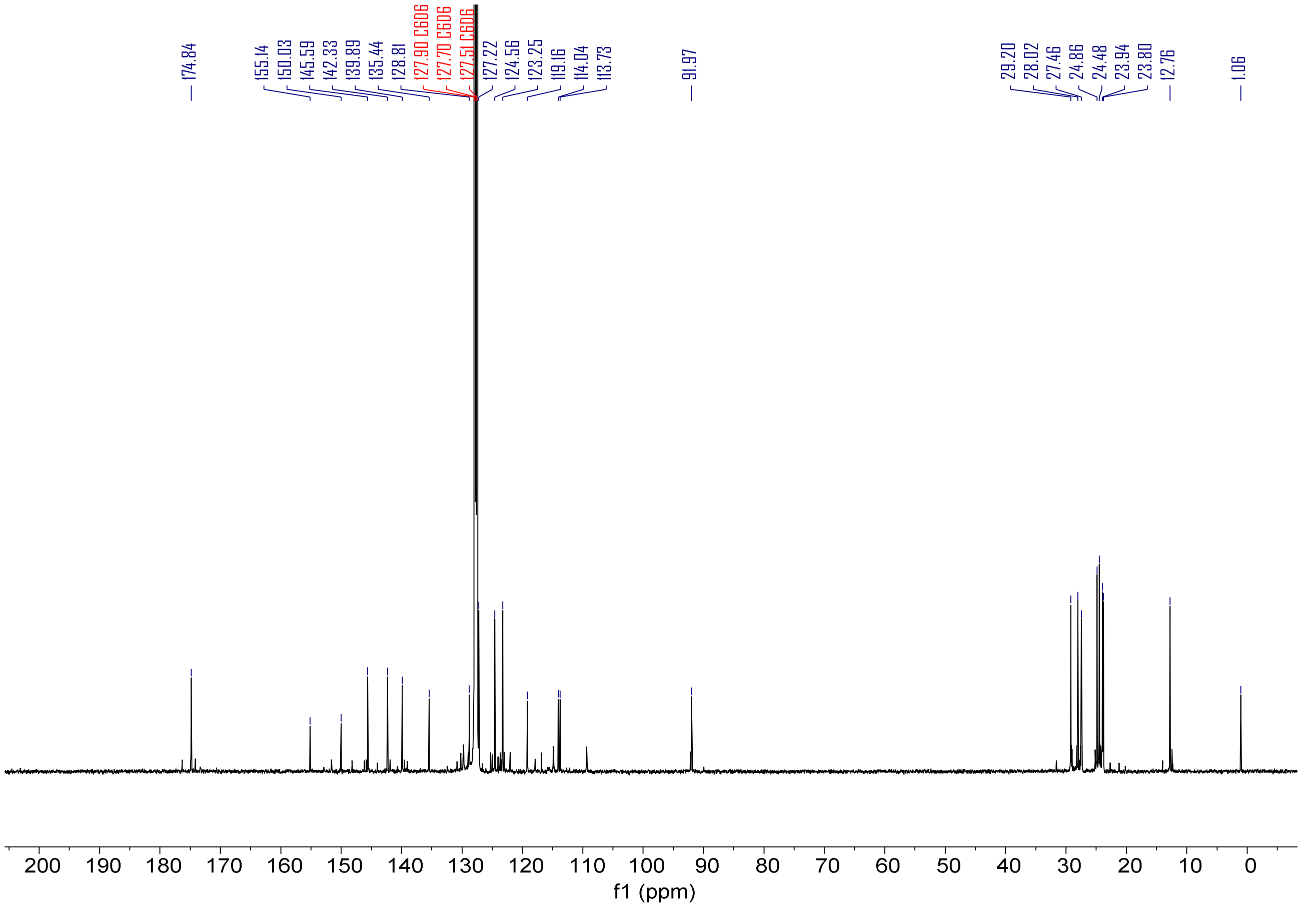

Figure S17. $^{13}C\{^1H\}$ NMR spectrum (125.7 MHz, C_6D_6, 298 K) of $[(^{EtDip}nacnac)Ga\{(C_6H_4)N(H)NPh\}]$ **5**.

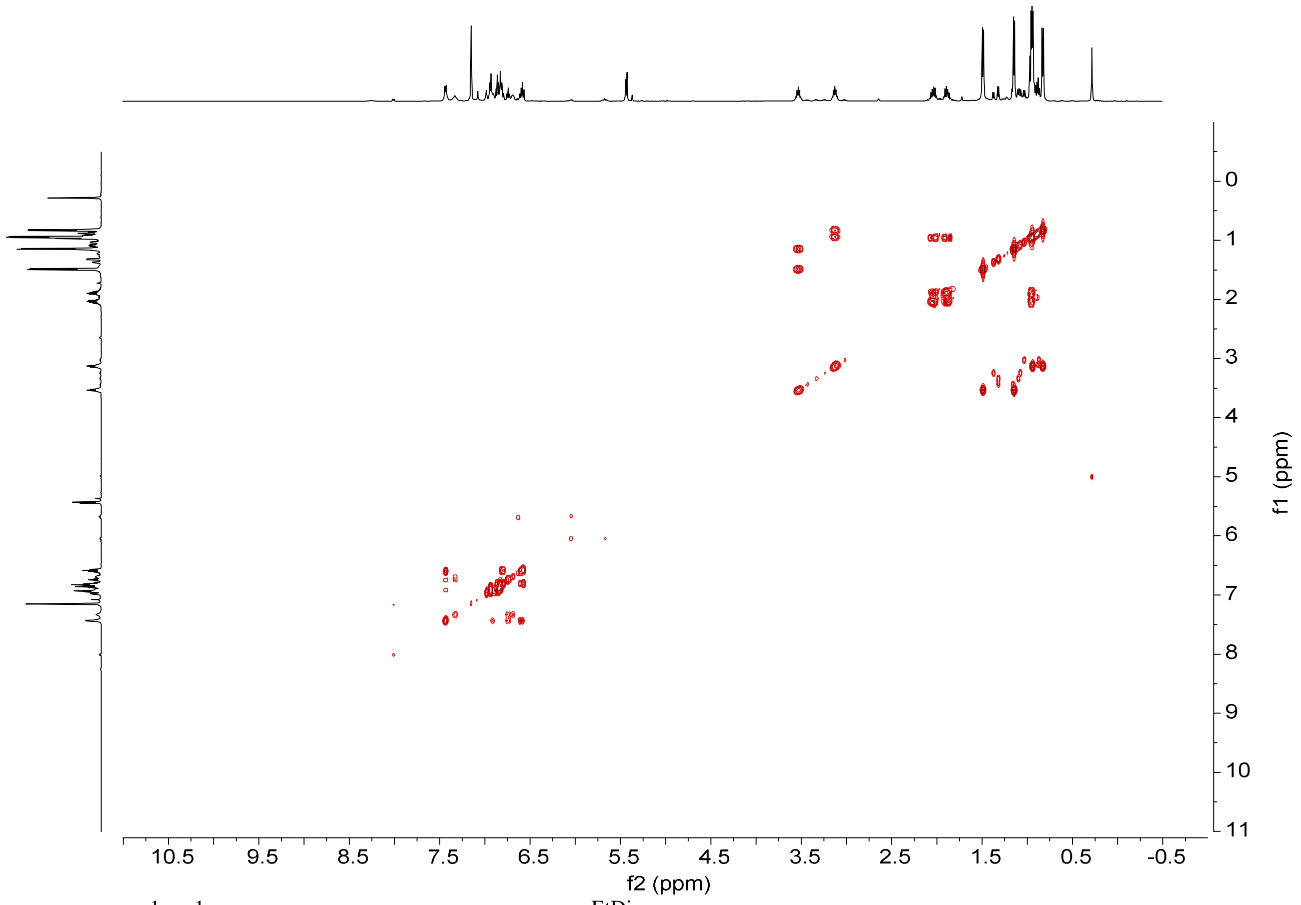

Figure S18. ^{1}H-^{1}H COSY NMR spectrum of [(^{EtDip}nacnac)Ga{(C_6H_4)N(H)NPh}] **5**.

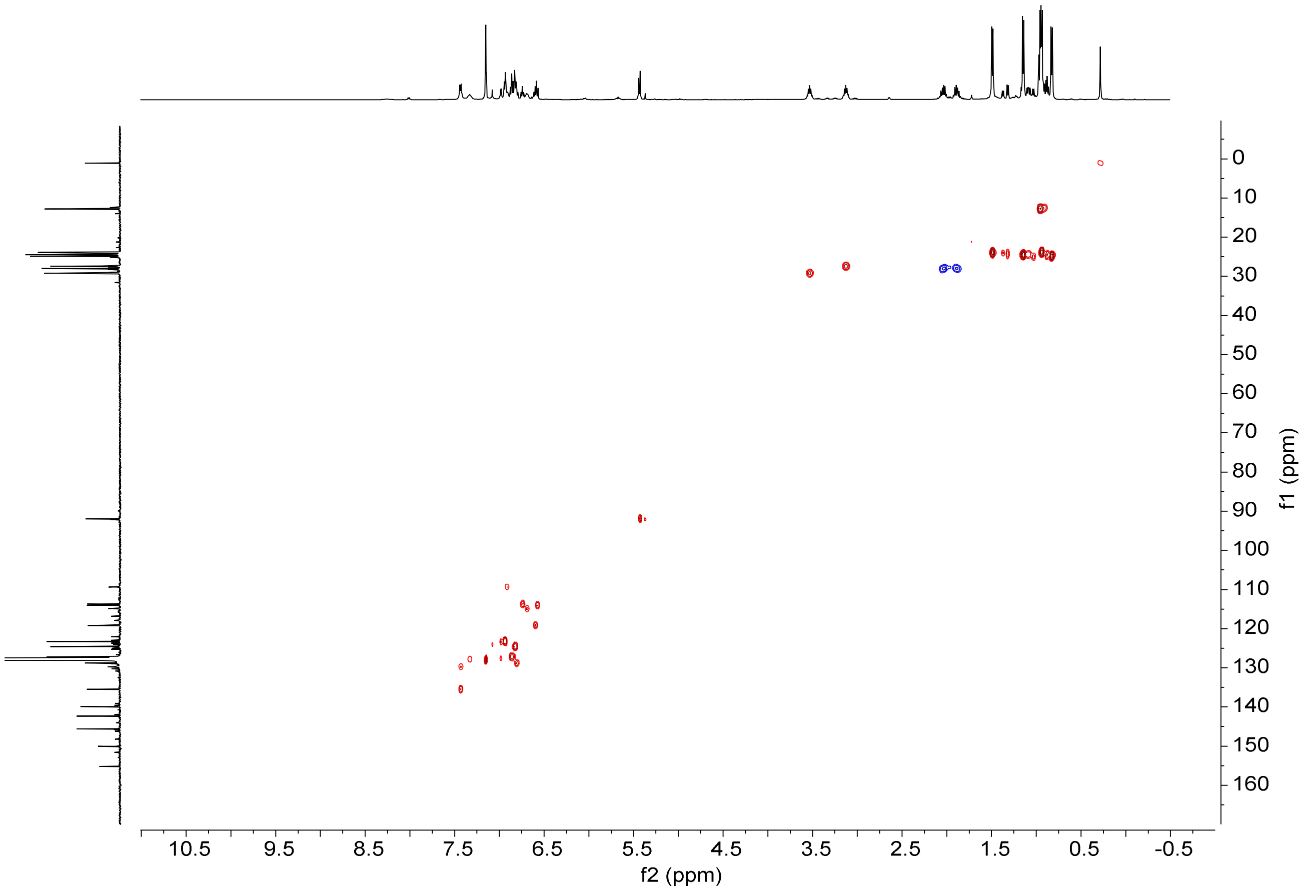

Figure S19. ^{1}H-^{13}C HSQC NMR spectrum of [(^{EtDip}nacnac)Ga{(C_6H_4)N(H)NPh}] **5**.

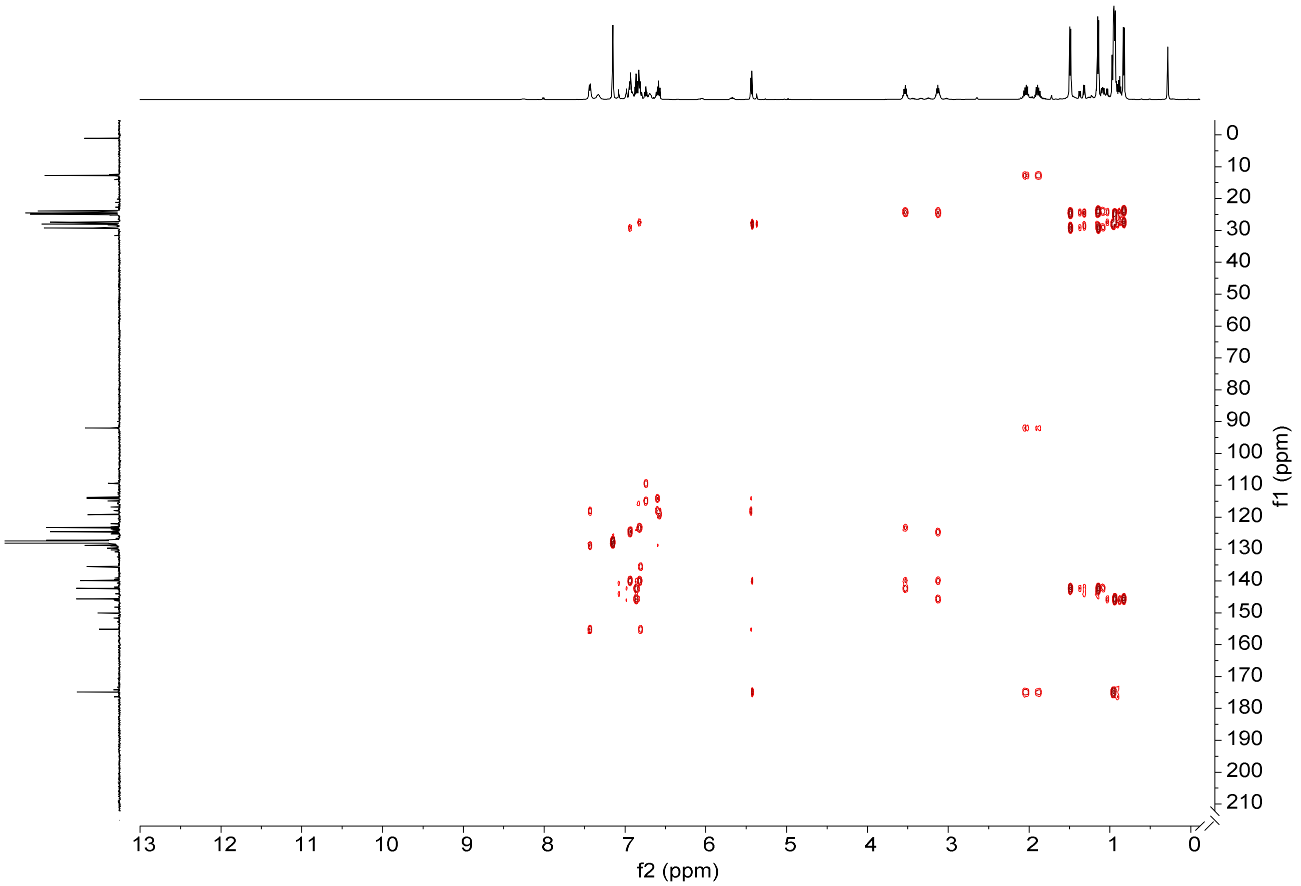

Figure S20. ^{1}H-^{13}C HMBC NMR spectrum of [(^{EtDip}nacnac)Ga{(C_6H_4)N(H)NPh}] **5**.

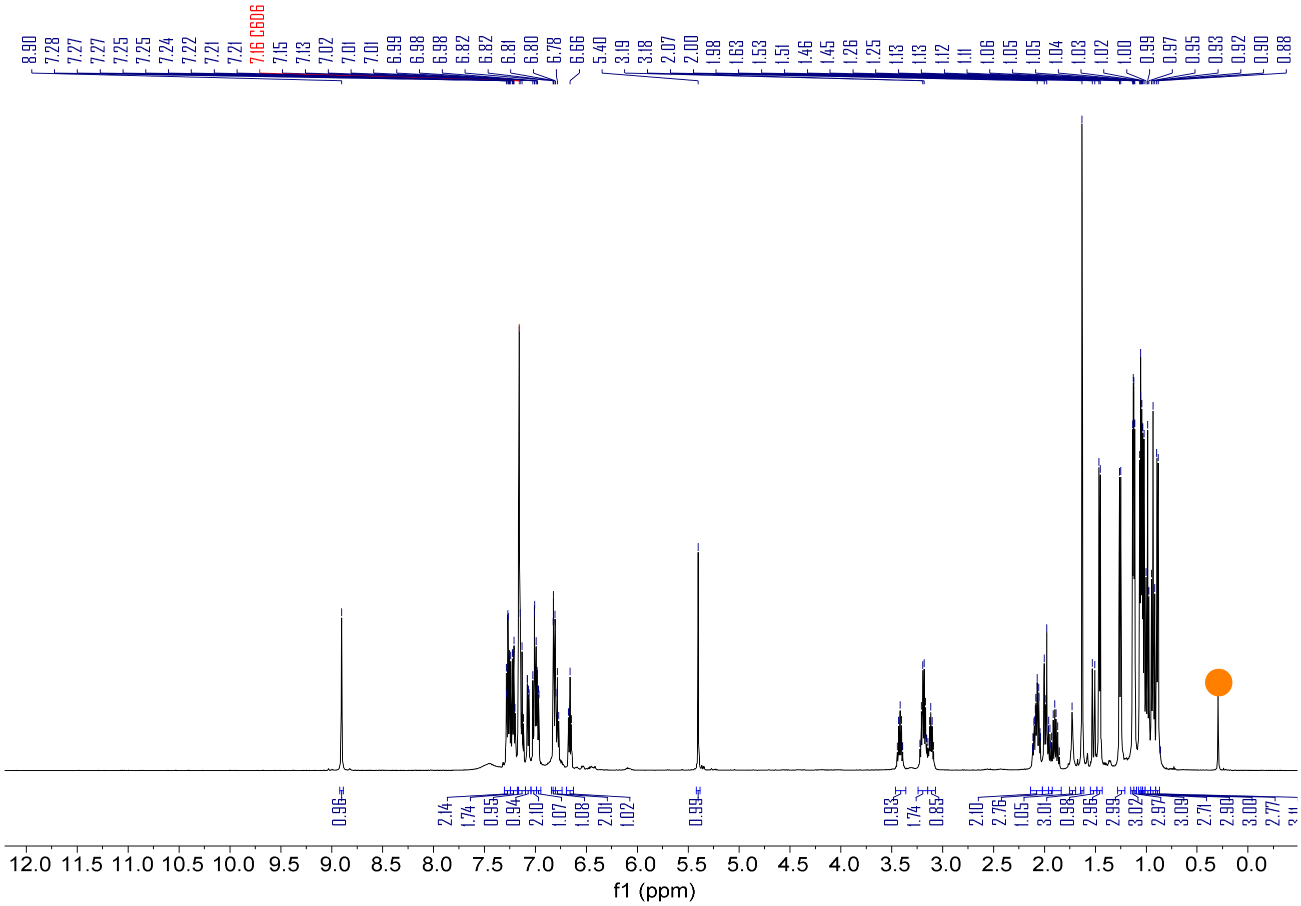

Figure S21. ^{1}H NMR spectrum (499.9 MHz, C_6D_6, 298 K) of [(^{EtDip}nacnac)Ga(PhNNHPh)($CH_2S(O)Me$)] **6**. The orange circle denotes chemical resonance of silicone grease.

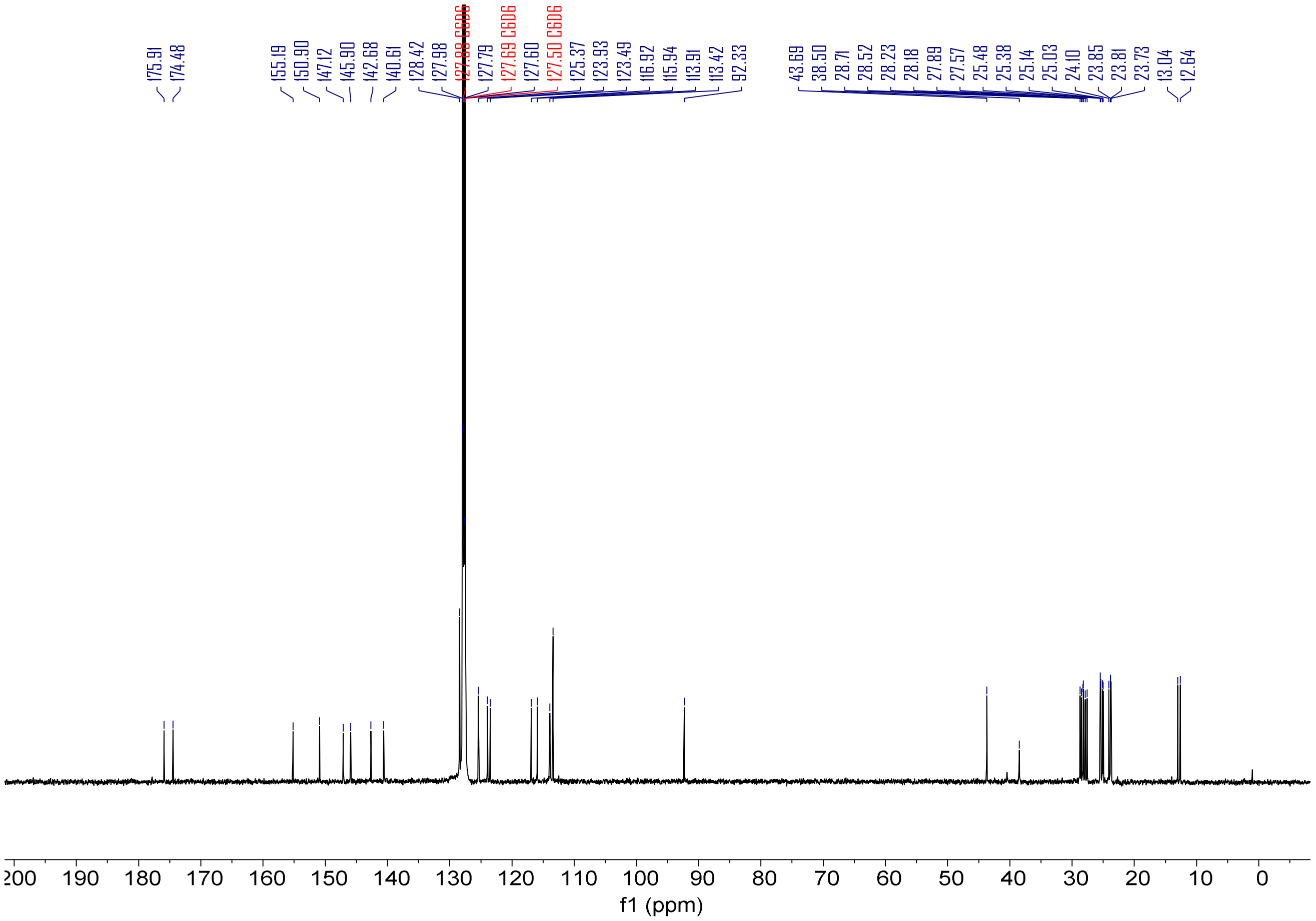

Figure S22. $^{13}C\{^{1}H\}$ NMR spectrum (125.7 MHz, C_6D_6, 298 K) of [(^{EtDip}nacnac)Ga(PhNNHPh)($CH_2S(O)Me$)] **6**.

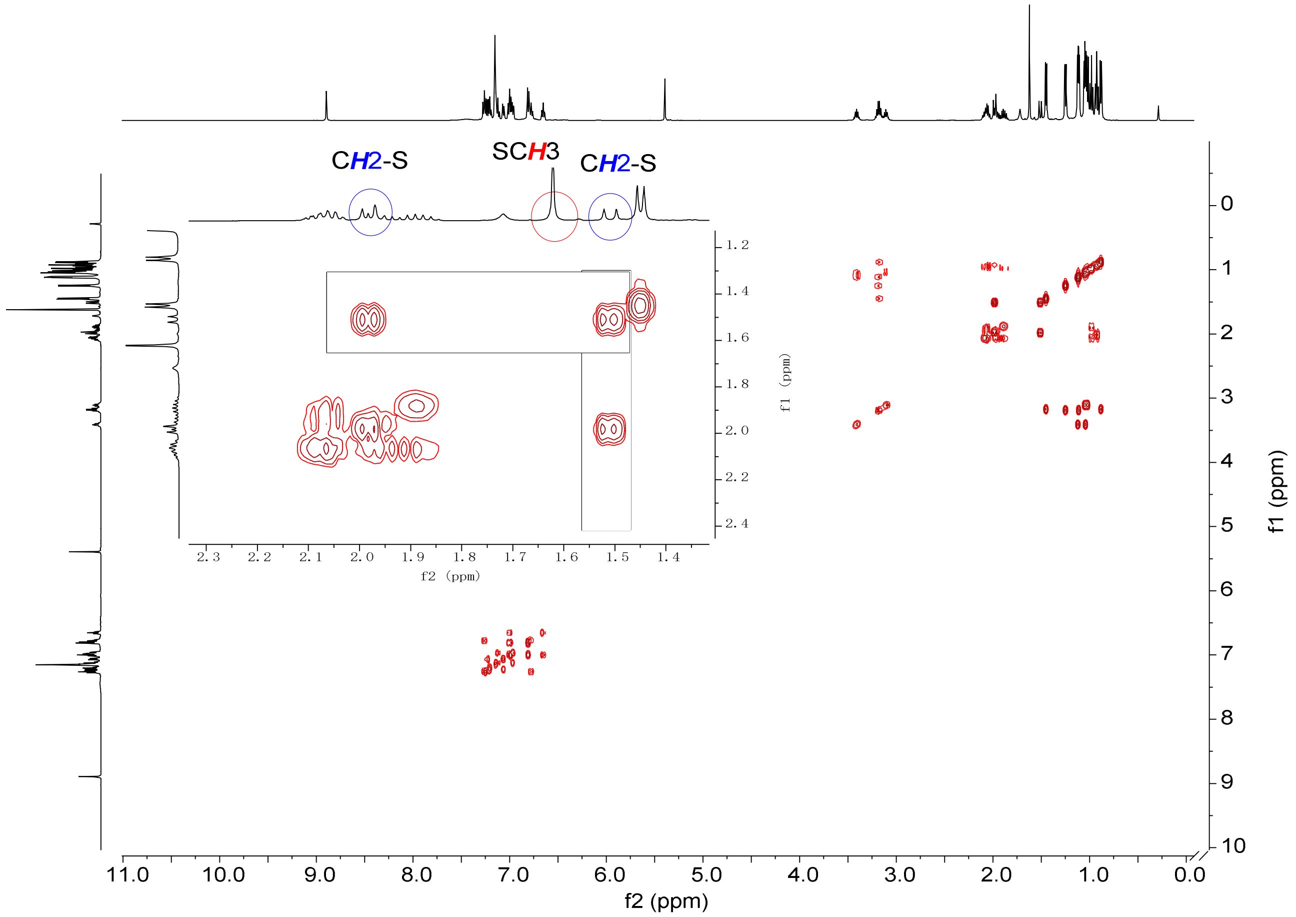

Figure S23. $^{1}H,^{1}H$ COSY NMR spectrum of [(^{EtDip}nacnac)Ga(PhNNHPh)($CH_2S(O)Me$)] **6**. The zoomed inset shows the coupling interactions (2 d) in the CH_2 unit.

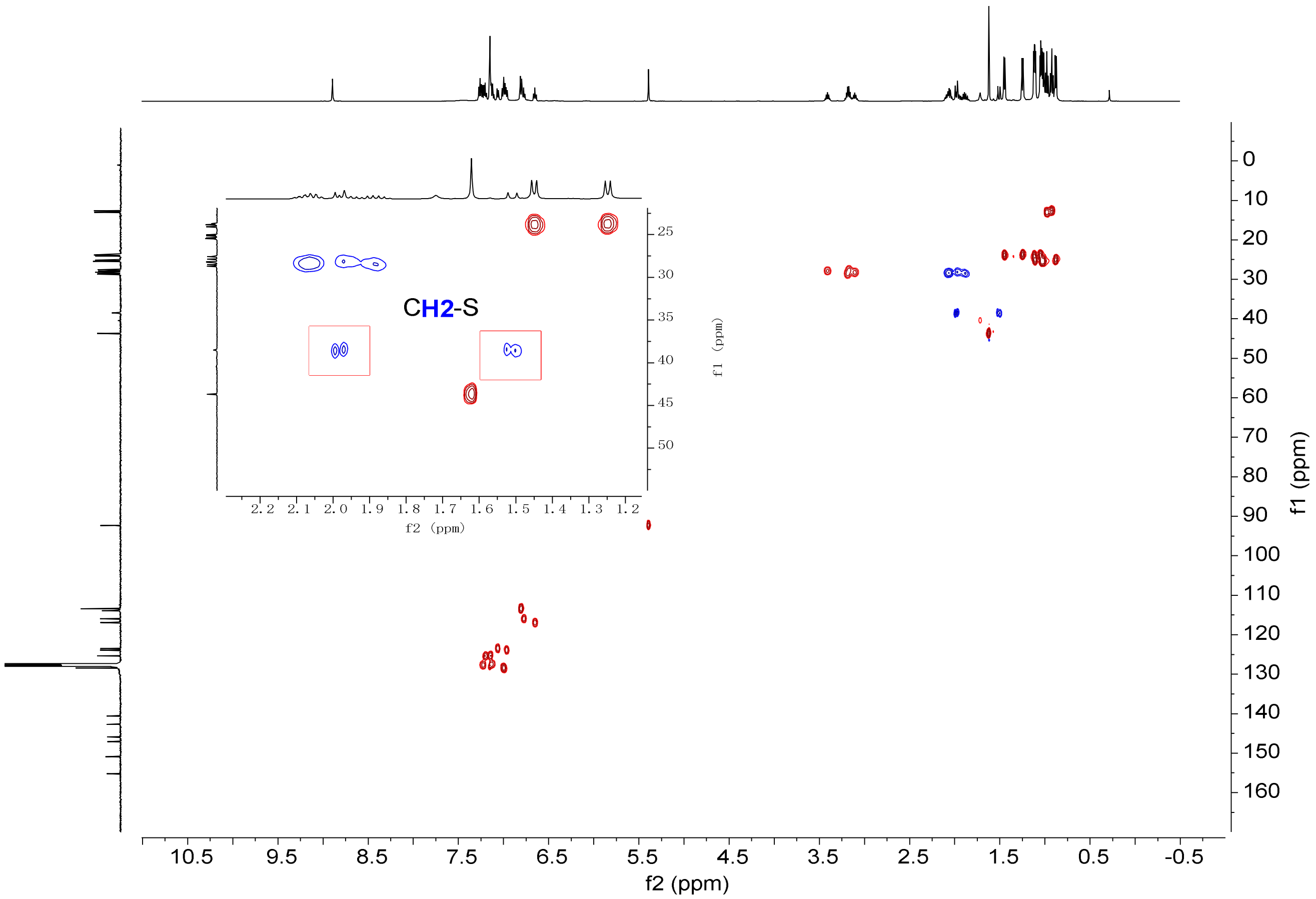

Figure S24. ^1H-^{13}C HSQC NMR spectrum of [(^{EtDip}nacnac)Ga(PhNNHPh)($CH_2S(O)Me$)] **6**. The zoomed inset shows an interactions in the *CH_2* unit and that both hydrogens bond to one carbon resonance.

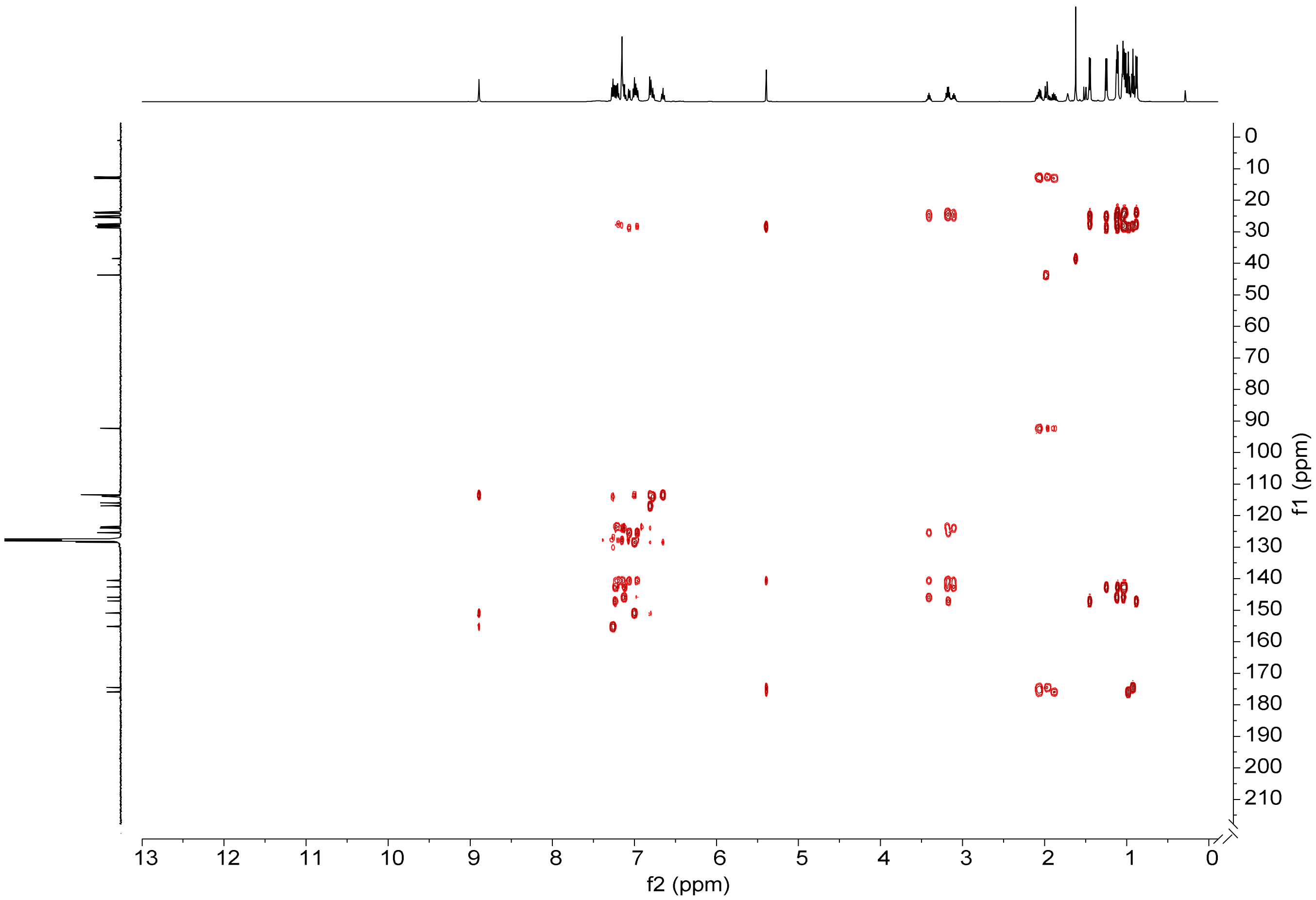

Figure S25. ^{1}H-^{13}C HMBC NMR spectrum of [(^{EtDip}nacnac)Ga(PhNNHPh)(CH_2S(O)Me)] **6**.

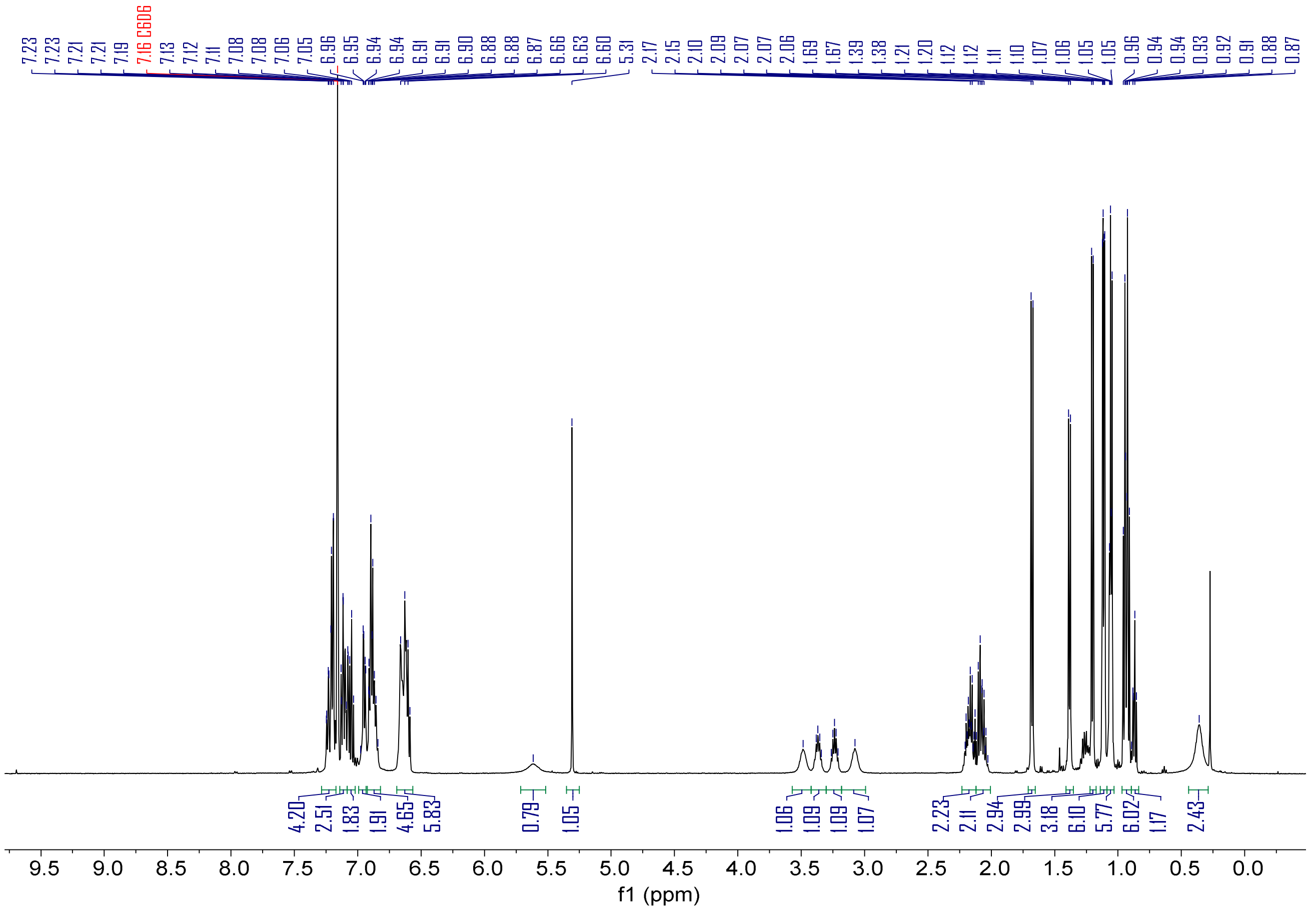

Figure S26. ^{1}H NMR spectrum (499.9 MHz, C_6D_6, 343 K) of [(^{EtDip}nacnac)Ga(PhNN(Ph)CH(Ph)O)] **7**.

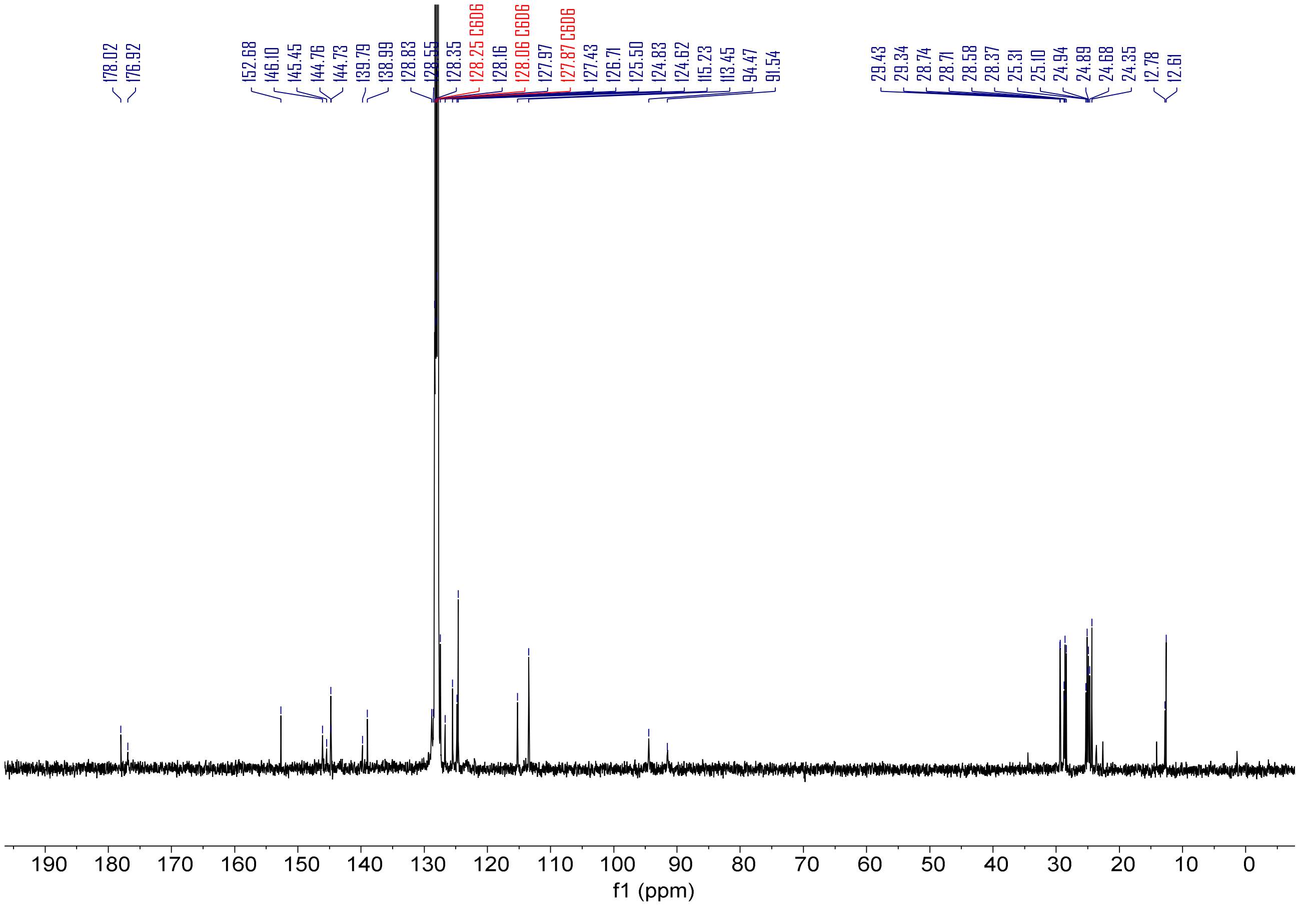

Figure S27. $^{13}C\{^{1}H\}$ NMR spectrum (125.7 MHz, C_6D_6, 343 K) of [(^{EtDip}nacnac)Ga(PhNN(Ph)CH(Ph)O)] **7**.

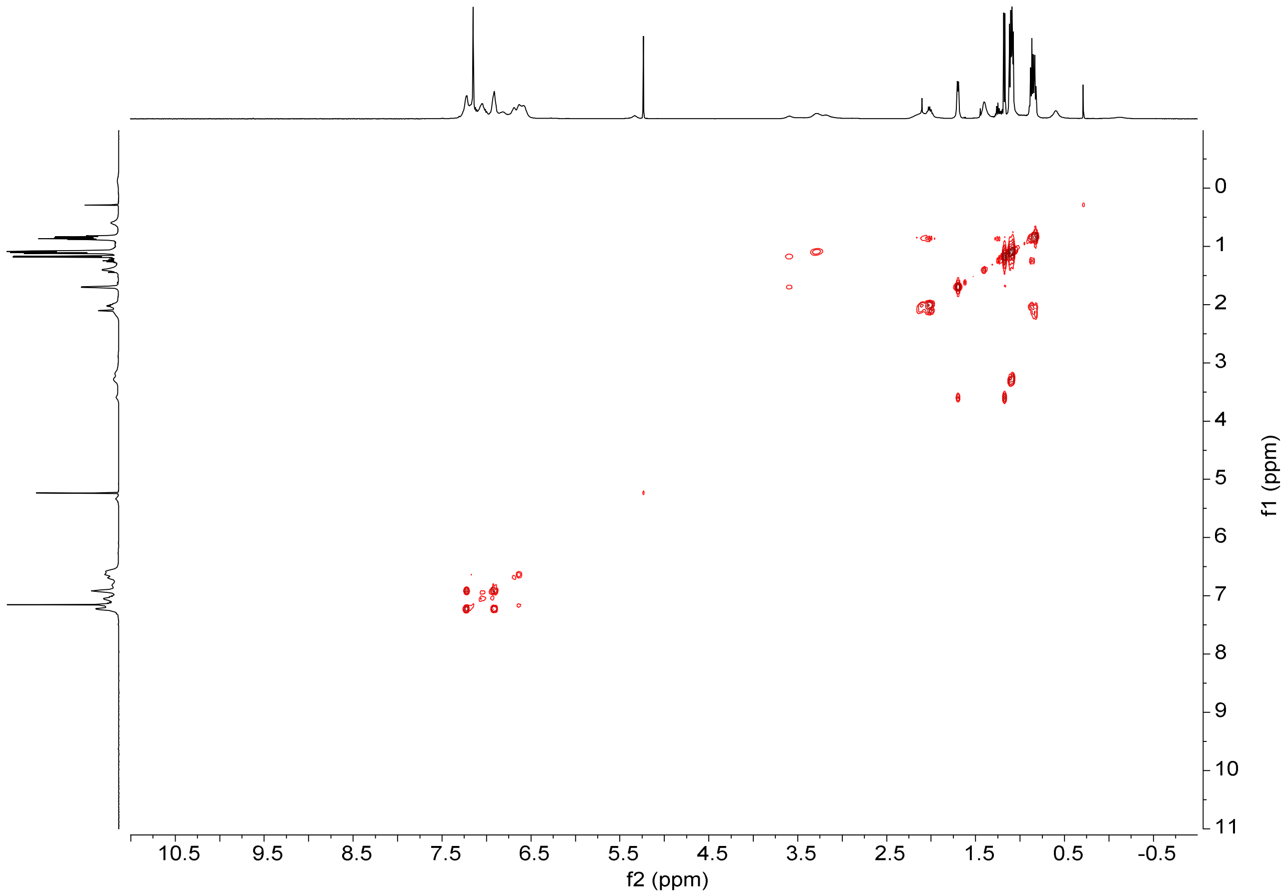

Figure S28. ^1H-^1H COSY NMR spectrum of [(^{EtDip}nacnac)Ga(PhNN(Ph)CH(Ph)O)] **7**.

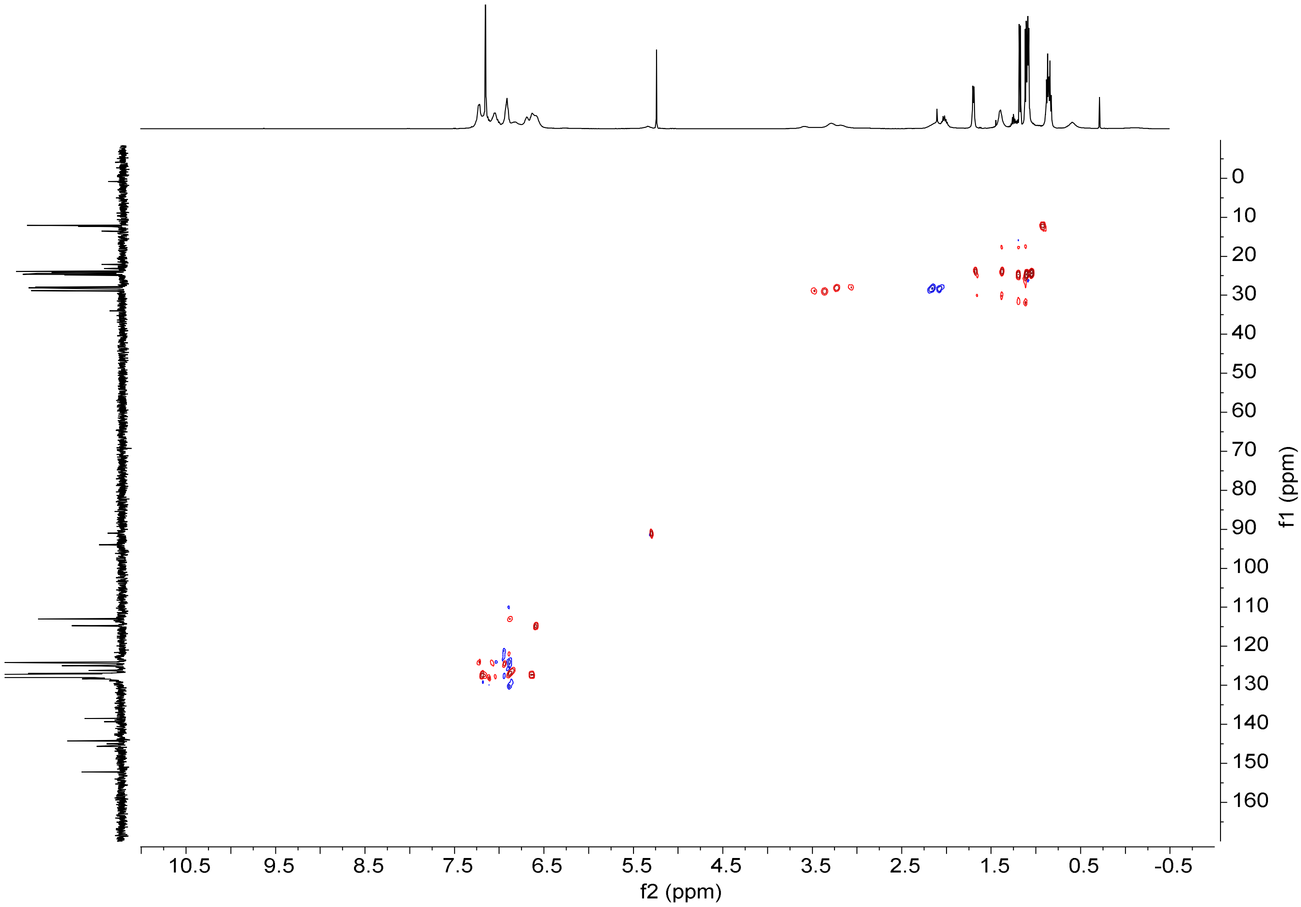

Figure S29. ^{1}H-^{13}C HSQC NMR spectrum of [(^{EtDip}nacnac)Ga(PhNN(Ph)CH(Ph)O)] **7**.

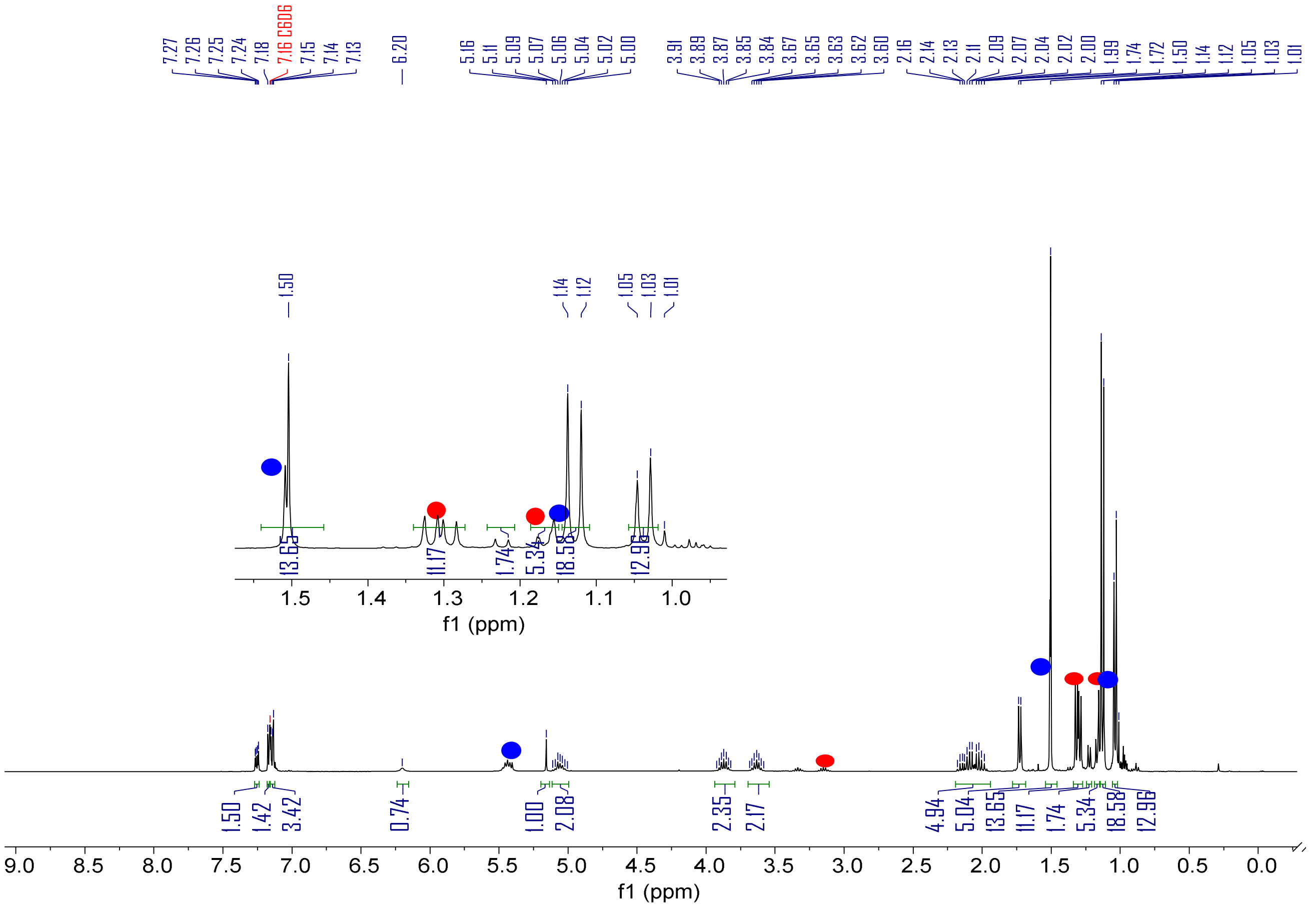

Figure S30. ^{1}H NMR spectrum (400.1 MHz, C_6D_6, 294 K) of [(^{EtDip}nacnac)Ga(H)–Al(H_2)(NHC)] **8** from the reaction of [(^{EtDip}nacnac)Ga] **2a** with (NHC)AlH_3 in deuterated benzene at room temperature for 2 days. Red circles denote the selected chemical resonances of residual **2a**, and blue circles denote the chemical resonances of residual (NHC)AlH_3.

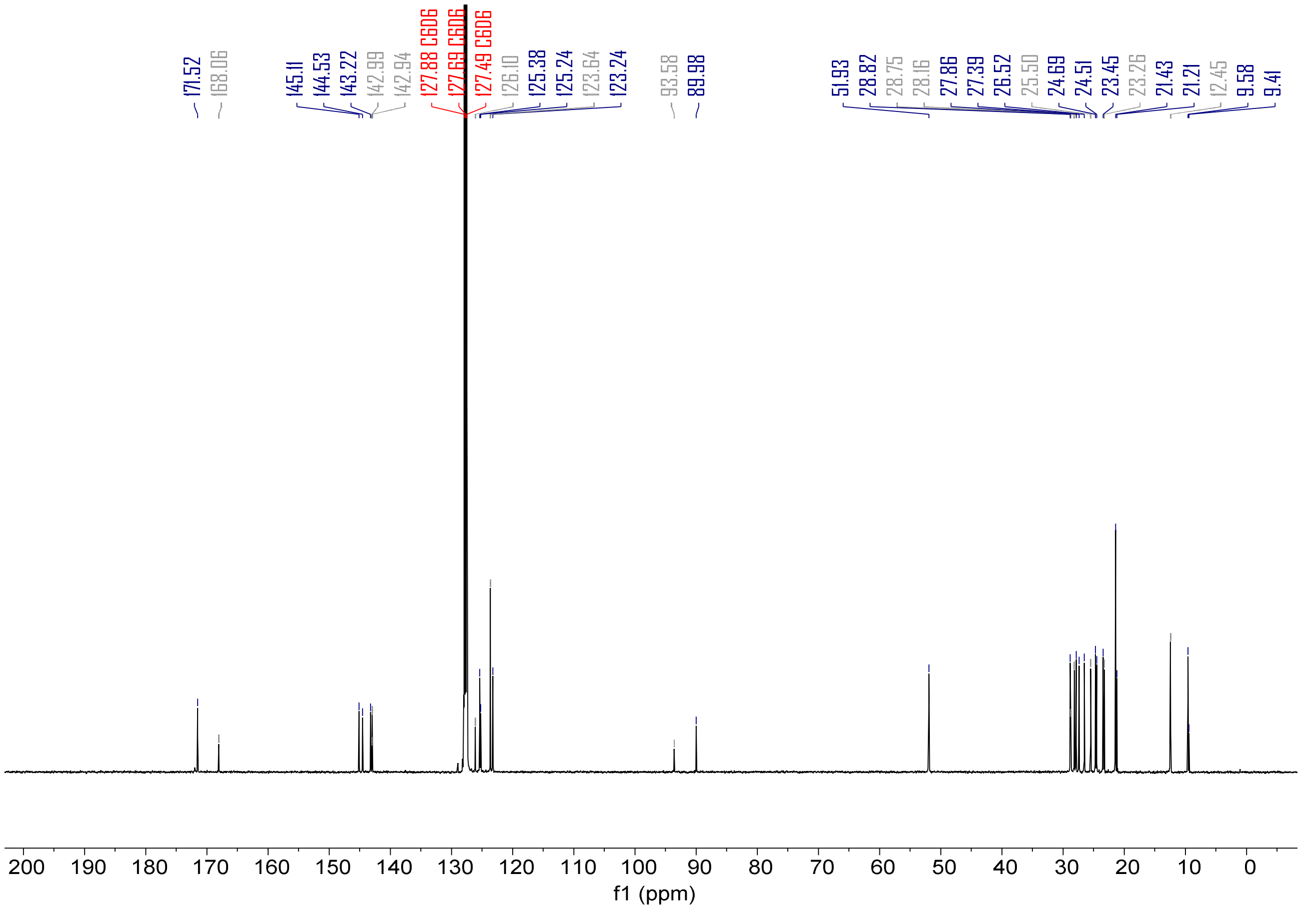

Figure S31. $^{13}C\{^{1}H\}$ NMR spectrum (125.7 MHz, C_6D_6, 298 K) of [(^{EtDip}nacnac)Ga(H)–Al(H_2)(NHC)] **8**. The grey-labelled peaks denote chemical resonances of residual [(^{EtDip}nacnacGa)] **2a**. Resonances for residual (NHC)AlH_3 are difficult to locate and mark and likely weak.

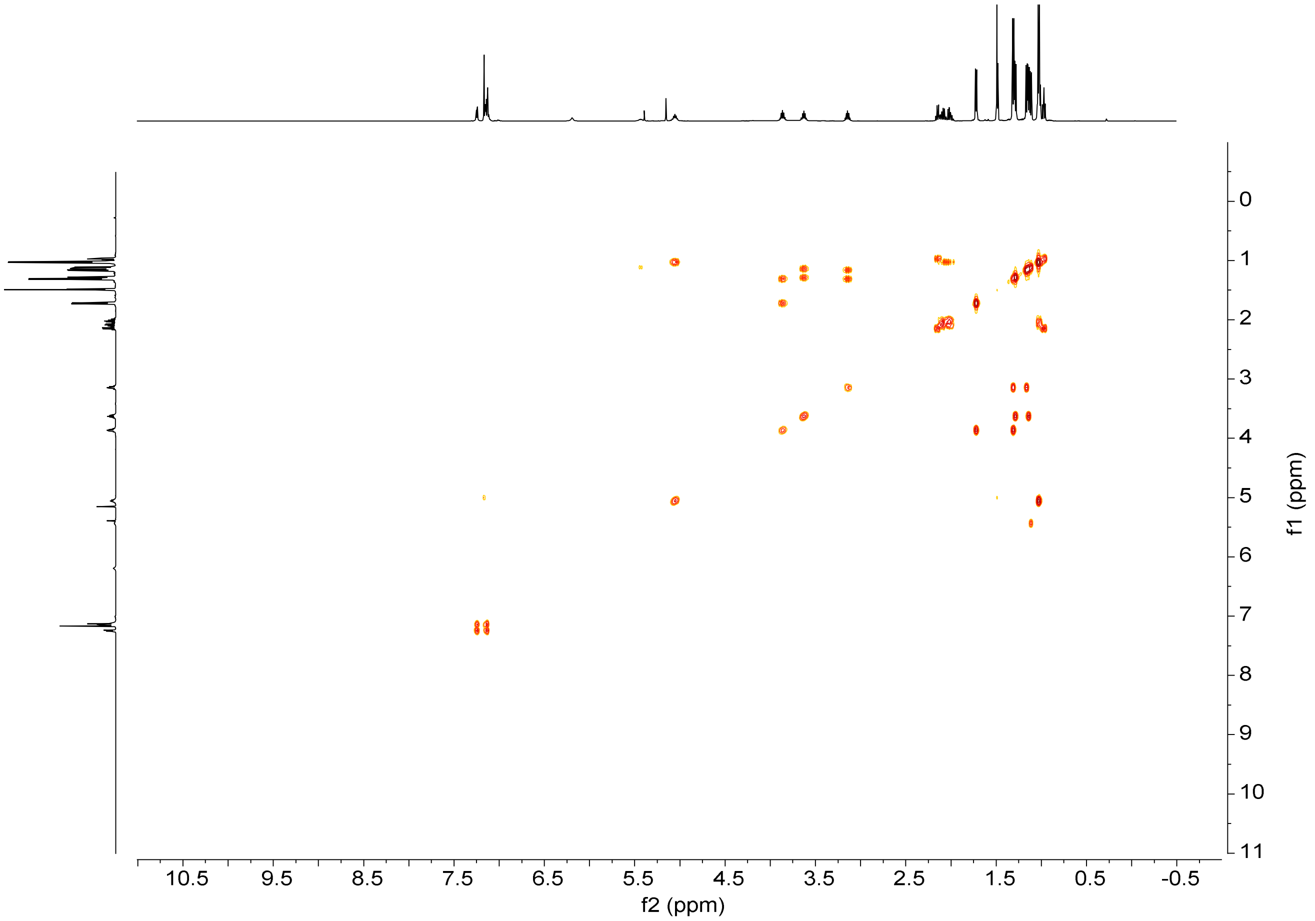

Figure S32. $^{1}H,^{1}H$ COSY NMR spectrum of [(^{EtDip}nacnac)Ga(H)–Al(H_2)(NHC)] **8**.

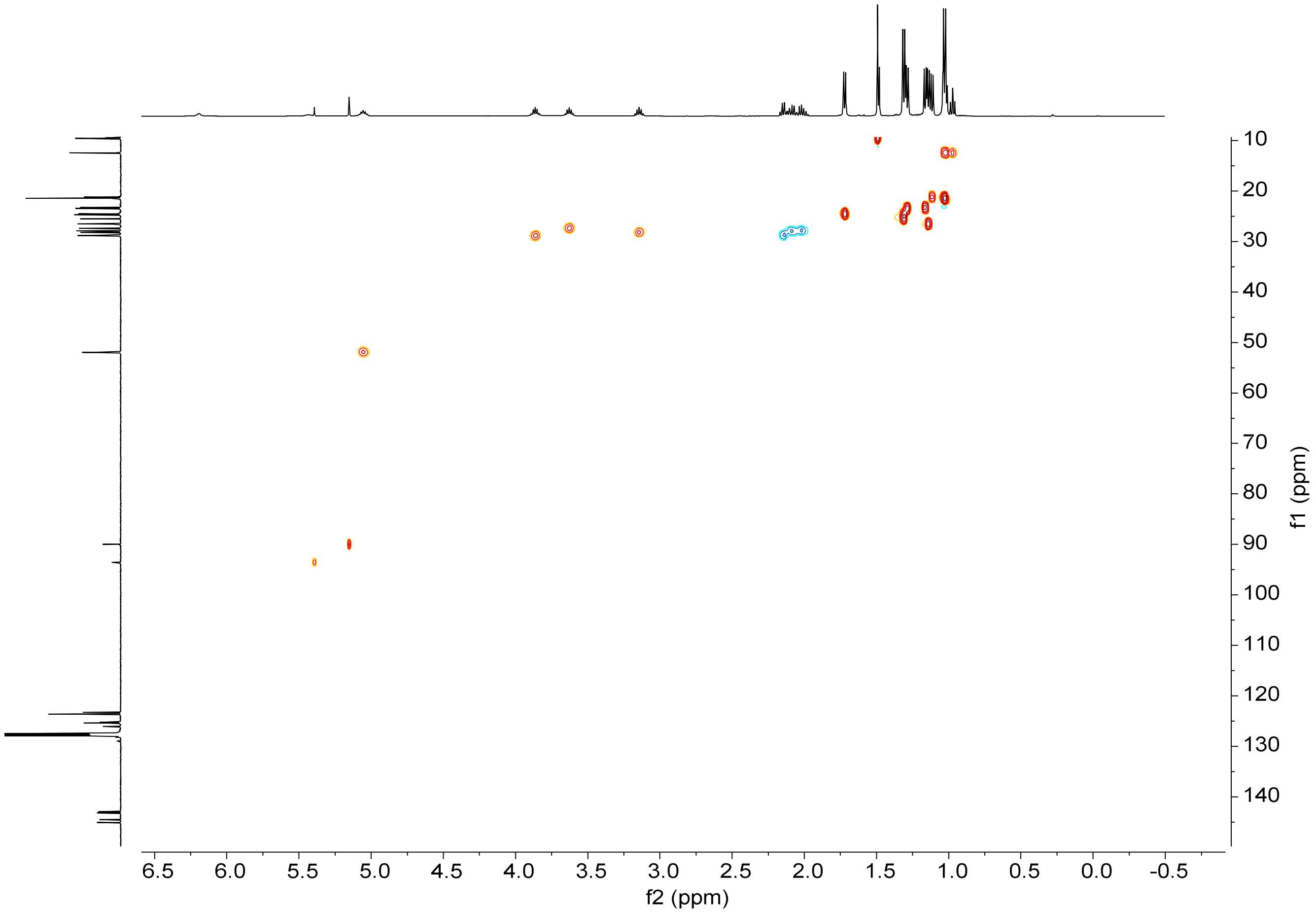

Figure S33. ^{1}H ^{13}C HSQC NMR spectrum of [(^{EtDip}nacnac)Ga(H)–Al(H_2)(NHC)] **8**.

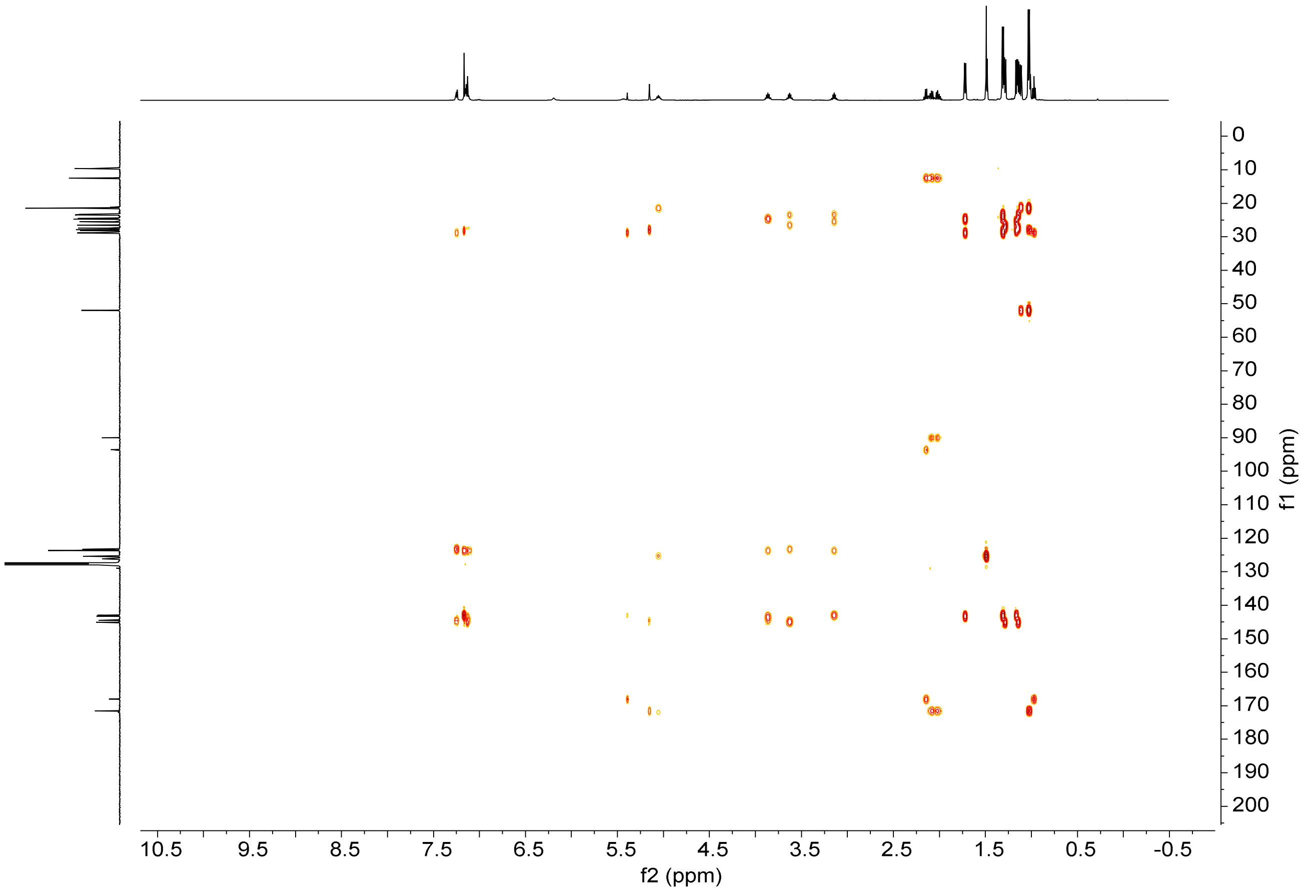

Figure S34. ^{1}H ^{13}C HMBC NMR spectrum of [(^{EtDip}nacnac)Ga(H)–Al(H_2)(NHC)] **8**.

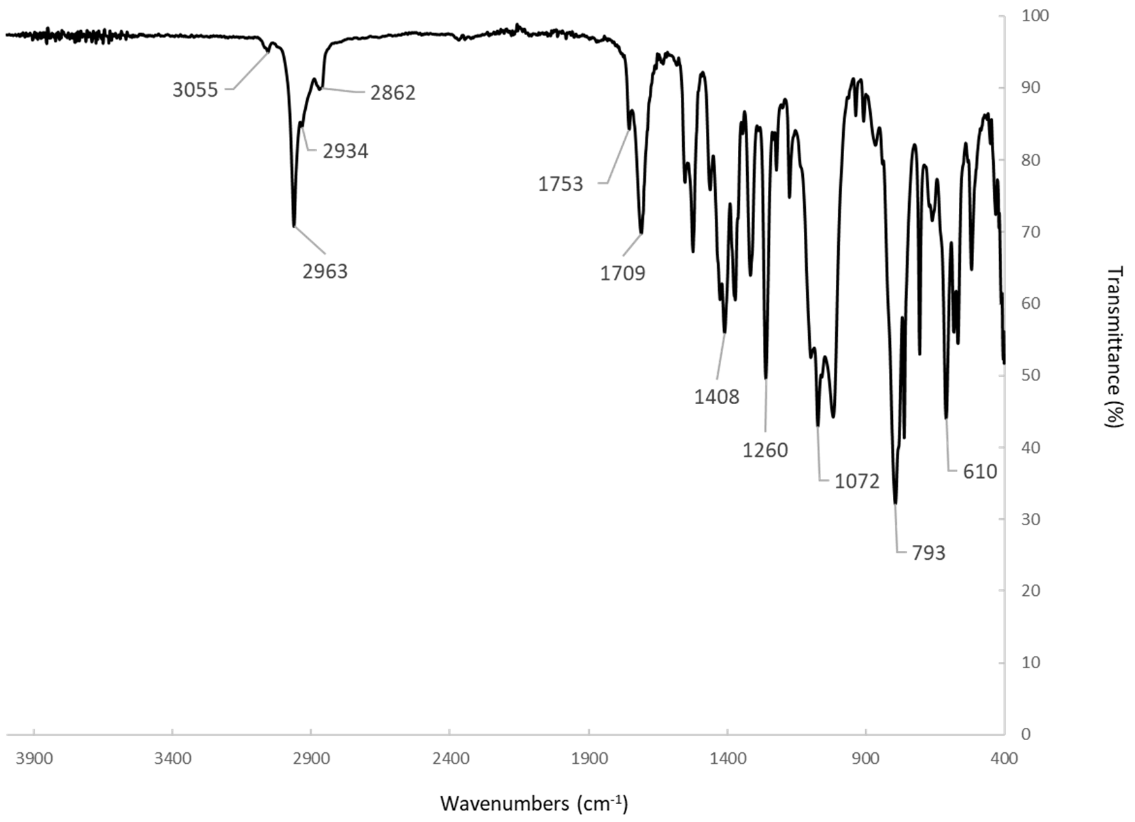

Figure S35. ATR-IR spectrum of [(^{EtDip}nacnac)Ga(H)–Al(H_2)(NHC)] **8**.

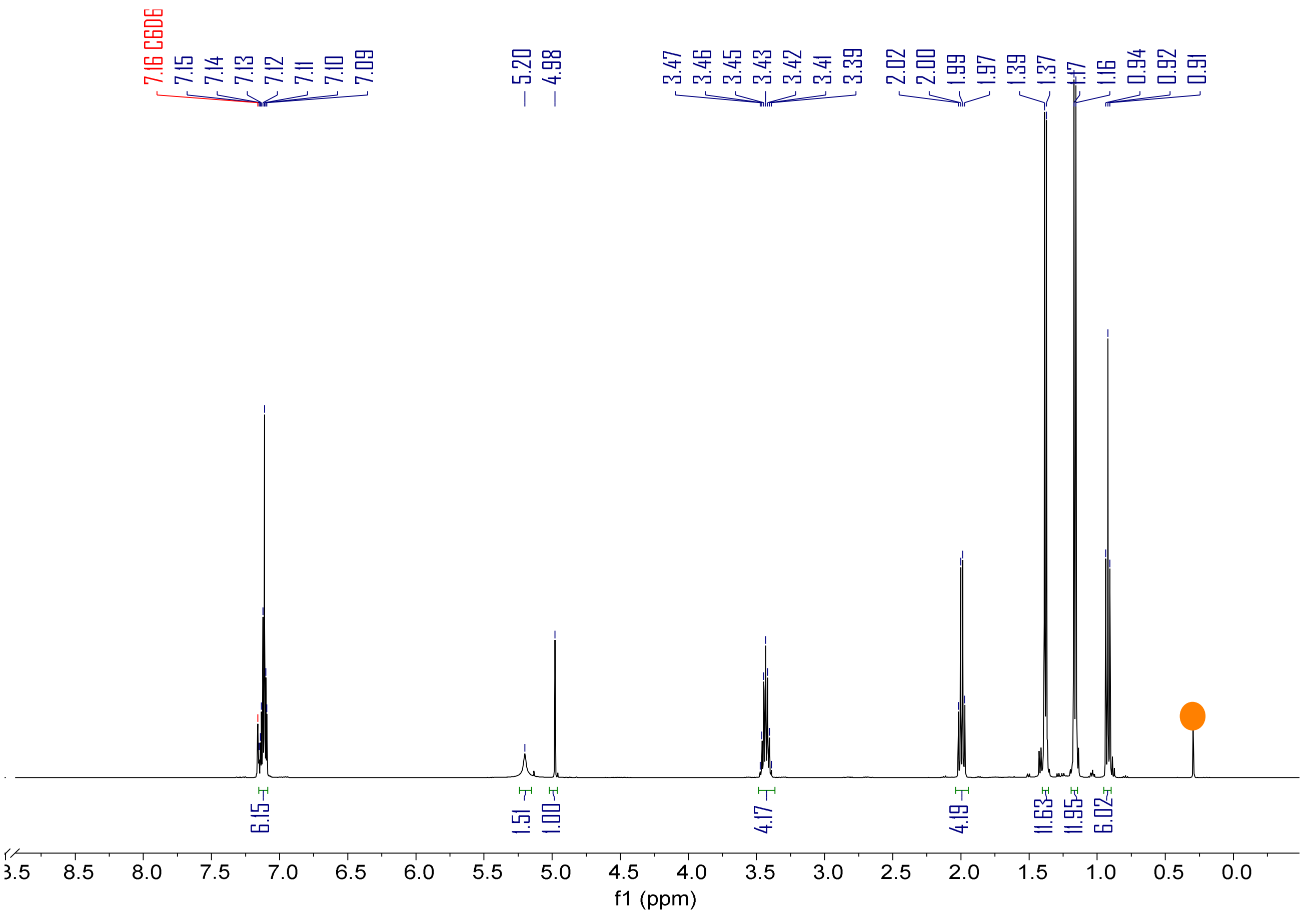

Figure S36. ^{1}H NMR spectrum (499.9 MHz, C_6D_6, 298 K) of [(^{EtDip}nacnac)GaH_2] **9**. The orange circle denotes the resonance of silicone grease.

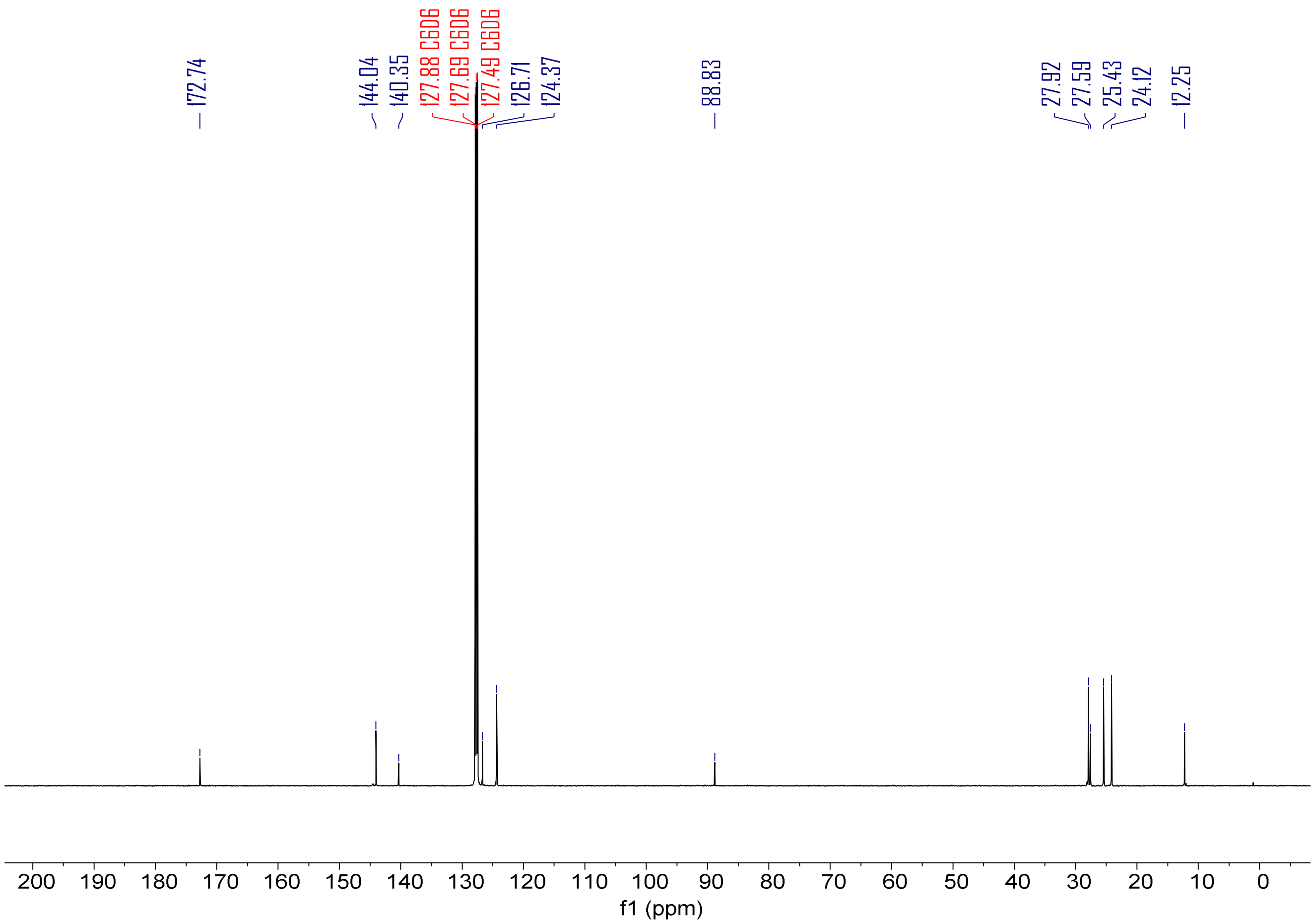

Figure S37. $^{13}C\{^{1}H\}$ NMR spectrum (125.7 MHz, C_6D_6, 298 K) of [(^{EtDip}nacnac)GaH_2] **9**.

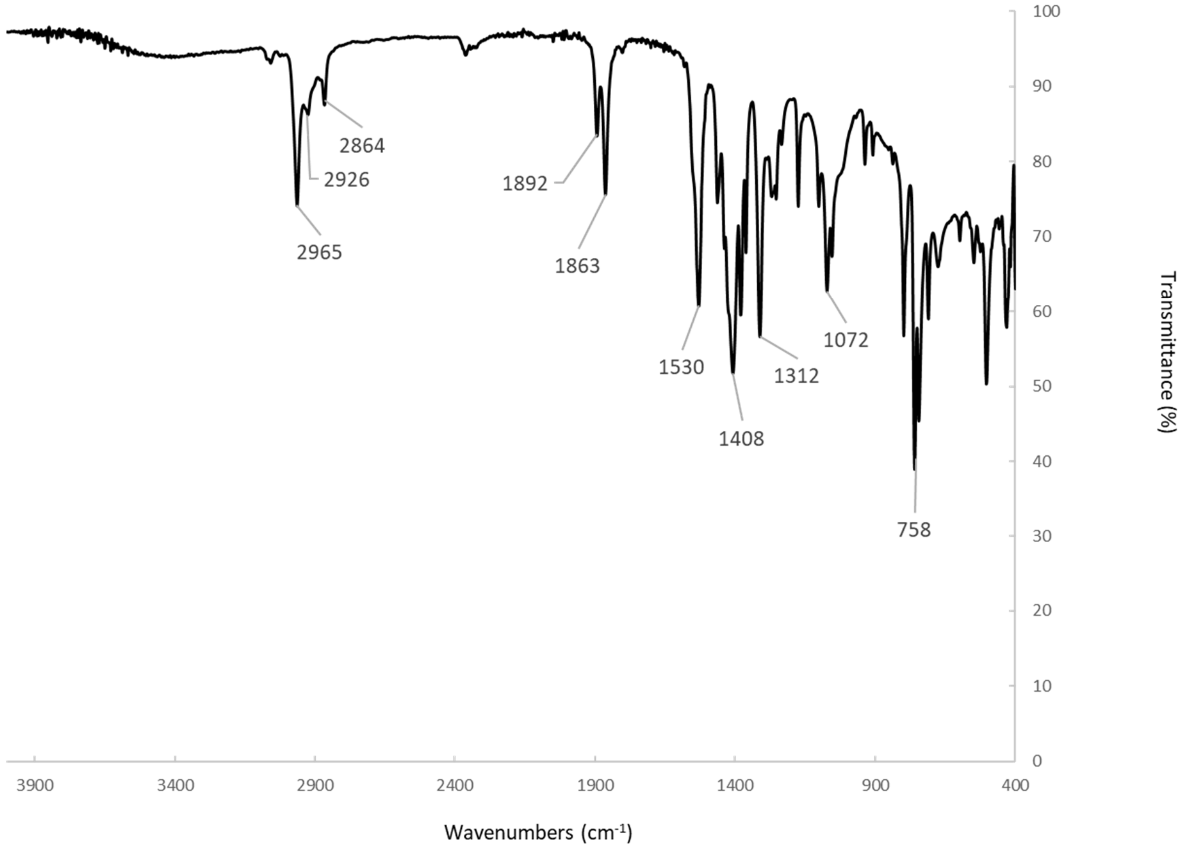

Figure S38. ATR-IR spectrum of [(^{EtDip}nacnac)GaH_2] **9**.

3 X-ray Crystallography

X-ray diffraction data for compounds **2a**, **2b**·3 C_6H_6, **3b**, **4a**, **4b**·0.5 C_6H_6, **7**·0.5 C_5H_{12}, **8**·0.5 C_6H_{14}, and **9** were collected using a Rigaku FR-X Ultrahigh Brilliance Microfocus RA generator/confocal optics with XtaLAB P200 diffractometer [Mo Kα radiation (λ = 0.71073 Å)]. Diffraction data for compounds **5** and **6**·$OSMe_2$ were collected using a Rigaku MM-007HF High Brilliance RA generator/confocal optics with XtaLAB P100 or P200 diffractometer [Cu Kα radiation (λ = 1.54187 Å)]. Data for all compounds analysed were collected and processed (including correction for Lorentz, polarization and absorption) using either CrystalClear[7] (data collected as a hemisphere of reciprocal space) or CrysAlisPro[8] (data collected using a calculated strategy). Structures were solved by dual-space methods (SHELXT[9]) and refined by full-matrix least-squares against F^2 (SHELXL-2019/3[10]). *Iso*-propyl groups in **4a** (C12-C14) and **6**·$OSMe_2$ (C81-C83) and ethyl groups in **2a** (C16-C17) and **7**·0.5 C_5H_{12} (C20, C21, C75, C76) showed disorder and each were modelled in two parts with geometric and/or thermal restraints. The gallium-coordinating carbons C43 in **4a** and C45 in **4b**·0.5 C_6H_6 were disordered and modelled in two parts with thermal motion restraints. Both dimethylsulfoxide solvates in **6**·$OSMe_2$ and the pentane solvate in **7**·0.5 C_5H_{12} showed disorder and were each modelled in two parts with geometric and/or thermal restraints. Non-hydrogen atoms were refined anisotropically, and hydrogen atoms were refined using a riding model except for the hydride hydrogen atoms on the Ga and Al atoms in **8**·0.5 C_6H_{14} and **9** which were located from the difference Fourier map and refined isotropically (in **8**·0.5 C_6H_{14}) or with U_{iso} riding on the parent atom (in **9**) without distance restraint.

Complex **2b**·3 C_6H_6 crystallised with a quarter of a main molecule in the asymmetric unit (and the lattice benzene molecules were refined using distance restraints). Complex **2a** crystallised with half a molecule in the asymmetric unit. Complexes **3b**, **4a**, **4b**·0.5 C_6H_6, **5**, and **8**·0.5 C_6H_{14} crystallised with a full main molecule in the asymmetric unit and complexes **6**·$OSMe_2$ and **7**·0.5 C_5H_{12} crystallised with two main molecules in the asymmetric unit. Complexes **9** crystallised with one full and two half independent molecules in the asymmetric unit. Images and selected bond lengths and angles for all structures can be found in the main text.

All calculations were performed using the Olex2[11] interface. Selected crystallographic data are presented in Table S1. CCDC 2523052–2523061 contains the supplementary crystallographic data for this paper. These data can be obtained free of charge from The Cambridge Crystallographic Data Centre via www.ccdc.cam.ac.uk/structures.

Table S1. Crystallographic data.

Compound reference	**2a**	**2b**·3 C_6H_6	**3b**
formula	$C_{31}H_{45}N_2Ga$	$C_{51}H_{67}N_2Ga$	$C_{33}H_{49}N_2GaI_2$
formula weight	515.41	777.78	797.26
temperature [K]	173	173	173
crystal description	Yellow prism	Colourless plate	Colourless prism
crystal size [mm^3]	0.09 × 0.08 × 0.05	0.04 × 0.03 × 0.01	0.20 × 0.05 × 0.05
space group	*Fdd*2	*Pmmn*	$P2_1/n$
a [Å]	43.0574(9)	11.1686(8)	9.7214(5)
b [Å]	15.8848(5)	24.4068(16)	17.0082(9)
c [Å]	8.42318(19)	8.1843(5)	21.7268(13)
α [°]	90	90	90
β [°]	90	90	101.0740(13)
γ [°]	90	90	90
vol [Å]^3	5761.1(2)	2231.0(3)	3525.5(3)
Z	8	2	4
ρ (calc) [g/cm^3]	1.188	1.158	1.502
μ [mm^{-1}]	0.975	0.652	2.554
$F(000)$	2208	836	1592
reflections collected	16000	28317	42141
independent reflections (R_{int})	3451 (0.0273)	2968 (0.0721)	6452 (0.0291)
parameters, restraints	180, 27	143, 9	355, 0
GoF on F^2	1.028	1.038	1.083
R_1 [$I > 2\sigma(I)$]s	0.0357	0.0479	0.0262
wR_2 (all data)	0.0833	0.1242	0.0680
largest diff. peak/hole [e/Å^3]	0.561/-0.194	0.458/-0.336	0.977/-1.133
Flack parameter	-0.022(4)	-	-
CCDC number	2523052	2523053	2523054

Table S1 continued 2. Crystallographic data.

Compound reference	**4a**	**4b**·0.5 C_6H_6	**5**
formula	$C_{43}H_{55}N_4Ga$	$C_{48}H_{62}N_4Ga$	$C_{43}H_{55}N_4Ga$
formula weight	697.63	764.73	679.63
temperature [K]	173	100	173
crystal description	Orange plate	Red prism	Yellow plate
crystal size [mm^3]	0.09 × 0.06 × 0.06	0.27 × 0.12 × 0.05	0.13 × 0.05 × 0.01
space group	$P2_1/n$	$P2_1/n$	$P\bar{1}$
a [Å]	12.7567(2)	10.3197(2)	11.6990(3)
b [Å]	17.6228(3)	20.4595(3)	12.7599(2)
c [Å]	17.6225(3)	20.1205(4)	13.0501(2)
α [°]	90	90	85.4027(15)
β [°]	103.8148(19)	97.718(2)	84.3941(17)
γ [°]	90	90	86.1444(17)
vol [Å]^3	3847.07(12)	4209.68(13)	1929.07(7)
Z	4	4	2
ρ (calc) [g/cm^3]	1.204	1.207	1.201
μ [mm^{-1}]	0.749	0.691	1.222
F(000)	1488	1636	744
reflections collected	83314	40143	34328
independent reflections (R_{int})	9462 (0.0413)	9646 (0.0312)	6776 (0.0508)
parameters, restraints	483, 22	500, 6	447, 1
GoF on F^2	1.015	1.024	1.031
R_1 [$I > 2\sigma(I)$]s	0.0367	0.0324	0.0353
wR_2 (all data)	0.0789	0.0789	0.0925
largest diff. peak/hole [e/Å^3]	0.331/-0.361	0.772/-0.385	0.794/-0.527
Flack parameter	-	-	-
CCDC number	2523060	2523055	2523057

Table S1 continued 3. Crystallographic data.

Compound reference	**6**·$OSMe_2$	**7**·0.5 C_5H_{12}	**8**·0.5 C_6H_{14}
formula	$C_{47}H_{67}N_4O_2S_2Ga$	$C_{52.50}H_{67}GaN_4O$	$C_{45}H_{75}N_4AlGa$
formula weight	853.88	839.82	768.79
temperature [K]	125	173	125
crystal description	Yellow block	Yellow plate	Yellow block
crystal size [mm^3]	0.12 × 0.06 × 0.04	0.09 × 0.09 × 0.02	0.15 × 0.07 × 0.02
space group	$P\bar{1}$	$P2_1/c$	$P\bar{1}$
a [Å]	10.55491(16)	20.6273(5)	9.8040(3)
b [Å]	19.7027(4)	21.1365(4)	11.7550(5)
c [Å]	23.5210(4)	22.1109(5)	20.7200(7)
α [°]	71.0904(17)	90	77.391(3)
β [°]	88.9870(14)	102.513(2)	87.537(2)
γ [°]	80.0646(14)	90	74782(3)
vol [Å]^3	4554.22(15)	9411.1(4)	2248.32(14)
Z	4	8	2
ρ (calc) [g/cm^3]	1.245	1.185	1.136
μ [mm^{-1}]	1.994	0.625	0.665
F(000)	1824	3592	834
reflections collected	82181	204060	48180
independent reflections (R_{int})	17110 (0.0709)	23074 (0.0640)	10543 (0.0664)
parameters, restraints	1134, 204	1159, 159	489, 0
GoF on F^2	1.026	1.018	1.142
R_1 [$I > 2\sigma(I)$]s	0.1006	0.0464	0.0655
wR_2 (all data)	0.2995	0.0955	0.1720
largest diff. peak/hole [e/Å^3]	2.025/-1.037	0.428/-0.311	2.160/-1.322
Flack parameter	-	-	-
CCDC number	2523059	2523058	2523056

Table S1 continued 4. Crystallographic data.

Compound reference	**9**
formula	$C_{31}H_{47}N_2Ga$
formula weight	517.42
temperature [K]	125
crystal description	Colourless prism
crystal size [mm^3]	0.07 × 0.06 × 0.06
space group	*I*2
a [Å]	12.9608(3)
b [Å]	16.9478(3)
c [Å]	26.9519(5)
α [°]	90
β [°]	99.0899(18)
γ [°]	90
vol [Å]^3	5848.80(18)
Z	8
ρ (calc) [g/cm^3]	1.176
μ [mm^{-1}]	0.961
F(000)	2224
reflections collected	52934
independent reflections (R_{int})	13517 (0.0585)
parameters, restraints	647, 7
GoF on F^2	1.097
R_1 [$I > 2\sigma(I)$]s	0.0648
wR_2 (all data)	0.1336
largest diff. peak/hole [e/Å^3]	0.597/-0.612
Flack parameter	0.024(8)
CCDC number	2523061

4 References

1 Bourne, C.; Dong, H.; McKain, K.; Mayer, L. C.; McKay, A. P.; Cordes, D. B.; Slawin, A. M. Z.; Stasch, A. Alkyl backbone variations in common β-diketiminate ligands and applications to N-heterocyclic silylene chemistry. *Dalton Trans.* **2024**, *53*, 9887–9895.

2 Green, M. L. H.; Mountford, P.; Smout, G. J.; Speel, R. S. New synthetic pathways into the organometallic chemistry of gallium. *Polyhedron* **1990**, *9*, 2763–2765.

3 Lochmann, L.; Trekoval, J. Lithium-potassium exchange in alkyllithium/potassium t-pentoxide systems: XIV. Interactions of alkoxides. *J. Organomet. Chem.* **1987**, *326*, 1–7.

4 Weitz, A., & Rabinovitz, M. Reduction of polycyclic hydrocarbons with potassium-graphite intercalate KC_8. *Synth. Met.* **1995**, *74*, 201–205.

5 Francis, M. D.; Hibbs, D. E.; Hursthouse, M. B.; Jones, C.; Smithies, N. A. Carbene complexes of Group 13 trihydrides: synthesis and characterisation of $[MH_3\{CN(Pr^i)C_2Me_2N(Pr^i)\}]$, M = Al, Ga or In. *J. Chem. Soc., Dalton Trans.* **1998**, 3249–3254.

6 (a) Ruff, J. K.; Hawthorne, M. F. The amine complexes of aluminum hydride. I. *J. Am. Chem. Soc.* **1960**, *82*, 2141–2144; (b) Fraser, G. W.; Greenwood, N. N.; Straughan, B. P. Aluminium Hydride Adducts of Trimethylamine: Vibrational Spectra and Structure. *J. Chem. Soc.* **1963**, 3742–3749.

7 *CrystalClear-SM Exper*t v2.1. Rigaku Americas, *The Woodlands, Texas, USA*, and Rigaku Corporation, *Tokyo, Japan*, 2015.

8 *CrysAlisPro* v1.171.41.93a, 42.53a, 42.82a, 42.93a, 42.94a, and 42.96a Rigaku Oxford Diffraction, Rigaku Corporation, *Tokyo,* Japan, 2020-2023.

9 Sheldrick, G. M. SHELXT – Integrated space-group and crystal structure determination. *Acta Crystallogr., Sect. A: Found. Adv*. **2015**, *71*, 3–8.

10 Sheldrick, G. M. Crystal structure refinement with SHELXL. *Acta Crystallogr., Sect. C: Struct. Chem.* **2015**, *71*, 3–8.

11 Dolomanov, O. V.; Bourhis, L. J.; Gildea, R. J.; Howard, J. A. K.; Puschmann, H. OLEX2: a complete structure solution, refinement and analysis program. *J. Appl. Crystallogr*. **2009**, *42*, 339–341.
